# Advancing SAR Target Recognition Through Hierarchical Self-Supervised Learning with Multi-Task Pretext Training

**DOI:** 10.3390/s26010122

**Published:** 2025-12-24

**Authors:** Md Al Siam, Dewan Fahim Noor, Mandoye Ndoye, Jesmin Farzana Khan

**Affiliations:** Electrical & Computer Engineering Department, Tuskegee University, Tuskegee, AL 36088, USA; msiam0229@tuskegee.edu (M.A.S.); mndoye@tuskegee.edu (M.N.); jkhan2@tuskegee.edu (J.F.K.)

**Keywords:** machine learning, deep learning, self-supervised learning (SSL), remote sensing, computer vision, signal processing, synthetic aperture radar (SAR), automatic target recognition (ATR)

## Abstract

Synthetic Aperture Radar (SAR) Automatic Target Recognition (ATR) systems face significant challenges due to limited labeled data availability and persistent domain gaps between synthetic and measured imagery. This paper presents a comprehensive self-supervised learning (SSL) framework that eliminates dependency on synthetic data while achieving state-of-the-art performance through multi-task pretext training and extensive downstream classifier evaluation. We systematically evaluate our SSL framework across diverse downstream classifiers spanning different computational paradigms and architectural families. Our study encompasses traditional machine learning approaches (SVM, Random Forest, XGBoost, Gradient Boosting), deep convolutional neural networks (ResNet, U-Net, MobileNet, EfficientNet), and a generative adversarial network. We conduct extensive experiments using the SAMPLE dataset with rigorous evaluation protocols. Results demonstrate that SSL significantly improves SAR ATR performance, with SVM achieving 99.63% accuracy, ResNet18 reaching 97.40% accuracy, and Random Forest demonstrating 99.26% accuracy. Our multi-task SSL framework employs nine carefully designed pretext tasks, including geometric invariance, signal robustness, and multi-scale analysis. Cross-validation experiments validate the generalizability and robustness of our findings. Rigorous comparison with SimCLR baseline validates that task-based SSL outperforms contrastive learning for SAR ATR. This work establishes a new paradigm for SAR ATR that leverages inherent radar data structure without synthetic augmentation, providing practical guidelines for deploying SSL-based SAR ATR systems and a foundation for future domain-specific self-supervised learning research in remote sensing applications.

## 1. Introduction

Synthetic Aperture Radar (SAR) systems have become indispensable tools for military surveillance, reconnaissance, and civilian monitoring applications because of their unique capability to provide high-resolution imagery regardless of weather conditions, cloud cover, or illumination. Unlike electro-optical (EO) sensors, SAR systems actively transmit electromagnetic signals and measure the backscattered energy, enabling consistent performance under diverse environmental conditions [1,2,3]. This all-weather capability makes SAR particularly valuable for modern defense systems and enables the rapid identification and classification of targets in complex operational environments [4,5].

The development of Automatic Target Recognition (ATR) systems for SAR imagery presents unique challenges that distinguish them from conventional computer vision tasks. SAR images exhibit “non-literal” characteristics where electromagnetic scattering properties dominate visual appearance, making human interpretation and traditional computer vision approaches less effective. The radar backscatter depends on complex interactions between electromagnetic waves and target geometry, material properties, and viewing angles. This results in imagery that can be fundamentally different from expectations in the optical domain [3].

A fundamental challenge in SAR ATR stems from the vast operating condition (OC) space that encompasses sensor parameters (frequency, polarization, resolution), target configurations (orientation, articulation), and environmental factors (weather, terrain, clutter) [6]. This multidimensional parameter space makes comprehensive data collection impractical, leading to the persistent problem of limited labeled training data for machine learning approaches [7]. Traditional datasets like MSTAR represent only a small fraction of the possible OC space, potentially limiting the generalizability of trained models [8].

To address data scarcity, researchers have extensively explored synthetic data generation using electromagnetic modeling and computational tools [7]. Sophisticated simulators such as RaySAR, CohRaS, and SARViz can generate realistic SAR imagery through ray tracing, full-wave electromagnetic modeling, and prediction of the center of scatter [9,10,11]. However, despite careful Computer-Aided Design (CAD) model truthing and parameter matching, persistent domain gaps between synthetic and measured imagery continue to limit ATR performance [3,12].

The SAMPLE dataset introduced by Lewis et al. represents a comprehensive effort to understand and quantify these domain gaps through carefully matched synthetic and measured SAR image pairs [3]. Their extensive experiments demonstrated that classification accuracy degrades significantly when synthetic data comprises more than 40% of the training set, even with meticulously truthed CAD models and matched collection parameters. This finding has highlighted the fundamental limitations of synthetic data approaches and motivated the exploration of alternative paradigms [3,12].

Traditional SAR ATR approaches are heavily based on supervised learning methods that require large amounts of labeled training data [13,14,15]. However, obtaining labeled SAR data presents significant challenges due to security considerations, expert annotation requirements, and the high cost of data collection campaigns. This data scarcity problem has motivated researchers to explore alternative learning paradigms, with self-supervised learning (SSL) emerging as a promising approach [15].

Self-supervised learning has emerged as a powerful paradigm for learning meaningful representations from unlabeled data across various domains. Recent advances in SSL have demonstrated remarkable success in computer vision through contrastive learning, masked autoencoding, and pretext task learning [16]. The core principle of SSL, the separation of supervision signals directly from the data structure without external labels, aligns well with the rich internal structure present in the SAR images [15,17]. Radar data contains inherent geometric relationships, electromagnetic scattering patterns, and coherent imaging properties that can be exploited for representation learning [16].

Despite the growing interest in SSL for SAR applications, there exists a significant gap in comprehensive evaluation frameworks that systematically assess the effectiveness of different SSL approaches across diverse downstream tasks and data availability scenarios. Most existing studies focus on specific model architectures or limited experimental settings, making it difficult to draw generalizable conclusions about the optimal deployment strategies for SSL-based SAR ATR systems [18].

This paper presents a comprehensive, improved self-supervised learning (SSL) framework specifically designed for synthetic aperture radar automatic target recognition (SAR ATR) applications. Our approach addresses the limitations of existing methods through several key innovations:

1. Multi-Task Pretext Learning Architecture: We develop a sophisticated SSL framework employing nine complementary pretext tasks specifically tailored to SAR imagery characteristics, including geometric transformations (rotations, flips), signal processing operations (denoising, blurring), and multi-scale analysis (zoom transformations).
2. Comprehensive Downstream Evaluation: We provide extensive comparison across diverse architectural families, including traditional machine learning approaches (SVM, XGBoost, Random Forest, Gradient Boosting) and modern deep learning architectures (ResNet18, U-Net, MobileNet variants, EfficientNet variants, and GAN-based classifiers) to demonstrate the versatility and effectiveness of our learned representations.
3. Elimination of Synthetic Data Dependency: Our framework demonstrates competitive performance using exclusively measured SAR data, directly addressing the fundamental domain gap problem that has limited previous approaches [3].
4. Operational Performance Analysis: We provide a comprehensive evaluation including timing analysis, false positive rate characterization, and computational efficiency metrics essential for operational deployment considerations.
5. Validation and Robustness: We perform a comprehensive analysis of performance across varying data availability scenarios (5% to 100% of training data), and cross-validation ensures reliable performance assessment.

The remainder of this paper is organized as follows. Section 2 provides a comprehensive review of related work in SAR ATR and self-supervised learning. Section 3 details our enhanced SSL framework, including architecture design, pretext tasks selection, and downstream evaluation methodology. Section 4 presents extensive experimental results and comparative evaluation. Section 5 discusses practical implications, limitations, and theoretical contributions as well as future research directions and broader impact considerations.

## 2. Related Work

### 2.1. Evolution of SAR Automatic Target Recognition

SAR ATR has evolved through several methodological paradigms over the past decades. Early approaches relied on template matching and correlation-based techniques, which compared measured SAR signatures with manually curated reference templates [5]. Although computationally efficient, these methods lacked robustness due to target aspect variations, articulation, and cluttered backgrounds. Subsequently, physics-informed methods introduced electromagnetic and geometric feature extraction techniques. Attributed Scattering Centers (ASC), target contour descriptors, and polarimetric signatures were employed to enhance target discrimination and interpretability [6,19]. However, these methods required extensive domain expertise and were often sensitive to sensor configurations and environmental noise.

The advent of traditional machine learning, particularly Support Vector Machines (SVM) and Random Forests, offered automated feature selection and improved generalization under moderate data availability [8]. Machine learning for SAR ATR involves two main steps: extracting discriminative features such as geometric structures, scattering characteristics, or Fourier-based transforms, and then classifying them using methods like SVM, K-NN, or neural networks [6].

Deep learning methods marked a major paradigm shift in SAR ATR. Convolutional Neural Networks (CNNs) was introduced to SAR by Morgan and Chen [20], which enabled end-to-end learning from raw SAR images. Recent advances have demonstrated that deep learning, with abundant training data, has the potential to greatly improve SAR ATR performance by establishing foundations for large-scale implementation [21]. The limited availability of high-quality SAR images has been identified as a critical bottleneck affecting the accuracy and robustness of target detection, classification, and segmentation tasks [22].

Recent studies, such as Li et al.’s SARATR-X [16], further demonstrate that foundation-level architectures tailored to radar data (e.g., including phase information, polarimetry, or multi-aspect views) outperform traditional pipelines. Hybrid models have also gained traction, combining domain-specific knowledge with learnable architectures. For instance, polarimetric CNNs leverage multi-channel SAR input to extract polarization-specific features [6]. Others incorporate synthetic aperture physics or prior target structure using attention layers or residual learning mechanisms [14]. Modern SAR ATR systems face significant deployment challenges due to the sequential arrival of training data and high retraining costs, motivating the development of incremental learning approaches [23]. Recent developments apply transformer architectures to sequence-like SAR inputs, moving beyond historic RNN/CNN designs. For instance, Li et al. propose a multi-aspect SAR target recognition transformer that mines inter-frame correlations using self-attention [24], while Zhao et al. highlight the transition from RNN/CNN to a lightweight vision transformer for SAR ATR in sequence contexts [25].

These developments underscore the importance of integrating radar phenomenology with modern learning frameworks, which is now enabling SAR ATR systems to reach operational readiness across a broader set of deployment conditions.

### 2.2. Synthetic Data Generation and Domain Gap Challenges

Synthetic SAR data generation has become a widely adopted approach to mitigate the scarcity of labeled data in SAR ATR tasks. Tools such as RaySAR, SARViz, and CohRaS facilitate the generation of ray-traced or wave-based SAR images by simulating electromagnetic backscatter under controlled conditions [9,10,11]. However, a persistent challenge lies in the domain gap between synthetic and measured SAR imagery, stemming from differences in background clutter, sensor noise, and real-world scene complexity [3,12]. Recent research has highlighted that advances in artificial intelligence have increased the demand for labeled data, often outpacing availability, while synthetic data generation provides a cost-effective alternative that still suffers from inherent domain shift problems [26].

Lewis et al. [3] introduced the SAMPLE dataset, which comprises paired synthetic and measured SAR image samples under matched parameters. Their study revealed that even with rigorous CAD truthing, classification performance degraded significantly when synthetic data accounted for more than 60% of the training set. Inkawhich et al. [12] further confirmed that models trained on synthetic data fail to generalize to real measurements, especially under domain shifts. Contemporary approaches have proposed hybrid dataset methods that combine synthetic and measured data to tackle the challenges hindering automatic target detection algorithms for ground targets in SAR images [27].

More recently, Kim et al. [7] proposed Soft Segmented Randomization (SSR) to improve domain generalization by applying controlled noise in segmentation masks. Despite these efforts, most methods cannot completely close the synthetic-to-real gap, thus motivating the need for learning frameworks that work exclusively with measured data. Novel diffusion-model-based approaches have emerged that require only single training samples to generate realistic SAR images, offering potential solutions to data scarcity challenges [22].

### 2.3. Self-Supervised Learning Paradigms

Self-supervised learning (SSL) has emerged as a promising approach to learning meaningful representations from unlabeled data [28]. In SAR, the structured nature of radar signals, including speckle statistics, aspect dependence, and coherence, provides rich signals for pretext learning tasks [14,15]. Recent work by Pei et al. [14] applied contrastive learning to SAR ATR and showed significant improvements over supervised baselines using the MSTAR and SAMPLE datasets. Li et al. [15] introduced a predictive gradient-based embedding architecture and demonstrated robust performance in low-data scenarios.

Foundation model initiatives, such as SARATR-X [16], leverage large-scale SSL to pretrain on multiple SAR modalities. Furthermore, Muzeau et al. [18] proposed SAFE, an SAR feature extractor based on masked Siamese ViTs, which achieves state-of-the-art results on several SAR benchmarks. Recent advances in adversarial self-supervised learning have introduced novel defense methods such as unsupervised adversarial contrastive learning (UACL), which explicitly suppresses vulnerability in the representation space by maximizing similarity between clean data and corresponding adversarial examples, demonstrating the potential of SSL to improve model robustness in SAR target recognition [29]. These methods highlight the potential of SSL in reducing dependency on labeled data while improving generalization.

Among contrastive learning approaches, SimCLR (Simple Framework for Contrastive Learning of Visual Representations) [30] has emerged as a foundational method in self-supervised learning. SimCLR learns representations by maximizing agreement between differently augmented views of the same image through a contrastive loss function, without requiring any task-specific labels during pretraining. The framework employs data augmentation, a learnable nonlinear projection head, and the normalized temperature-scaled cross-entropy (NT-Xent) loss to train encoders that produce invariant representations. While originally developed for natural images, SimCLR’s simplicity and effectiveness have led to its adoption across diverse imaging domains. However, its application to SAR imagery presents unique challenges due to fundamental differences in image formation physics, the scarcity of large-scale SAR datasets compared to natural image collections, and domain-specific characteristics such as speckle noise and aspect-dependent scattering [31]. Despite these challenges, SimCLR represents an important baseline for evaluating SSL approaches in SAR ATR, as it enables direct comparison between general contrastive learning methods and domain-specific pretext task approaches.

### 2.4. Multi-Task Learning and Feature Representation

In SAR applications, auxiliary tasks can potentially align with underlying properties of radar imaging, such as translation and orientation invariance. Our previous work [32] showed that multi-task pretext training with complementary transformation tasks enables robust feature learning from measured SAR data without synthetic augmentation. Our proposed SSL framework employs nine tasks ranging from geometric to signal processing transformations, designed to learn robust and transferable feature embeddings.

## 3. Materials and Methods

### 3.1. Problem Formulation and Framework Overview

Let $X\;=\;{\left\{x_{i}\right\}}_{i=1}^{N}$ represent a dataset of *N* measured SAR images, where each $x_{i}\in R^{64\times 64}$ is a single-channel image of spatial dimensions 64×64. Our goal is to learn a feature representation function $f_{\theta }:R^{64\times 64}\to R^{2048}$ parameterized by θ that maps input images to 2048-length feature vectors suitable for downstream target classification. The self-supervised learning framework consists of three main components:

1. Pretext Learning Phase: Learn feature representations $f_{\theta }$ by training on multiple pretext tasks ${\left\{T_{k}\right\}}_{k=1}^{K}$ using unlabeled SAR imagery.
2. Feature Extraction Phase: Apply the learned encoder $f_{\theta }$ to extract features from training and test data.
3. Downstream Classification Phase: Train classifiers on the extracted features for target recognition.

**Figure 1:**
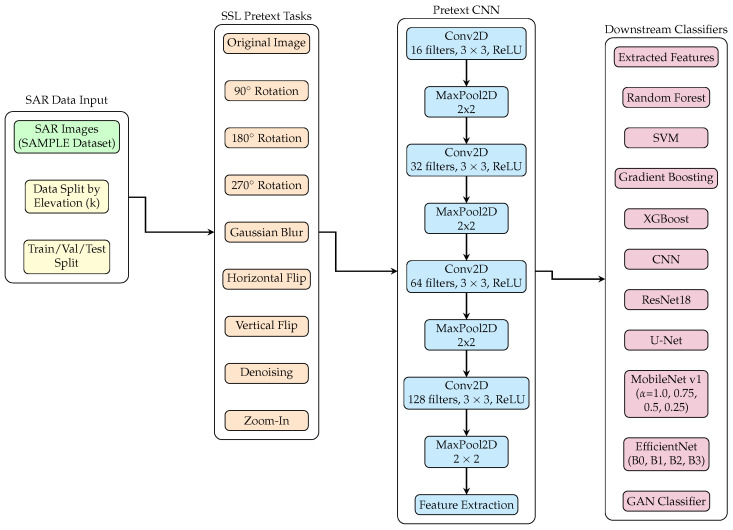
Detailed architecture of the proposed self-supervised learning framework. Main stages: (1) Input Processing: converting raw SAR images to normalized format with elevation-based data splitting, (2) Pretext Tasks: nine distinct transformation tasks for self-supervised learning, (3) CNN Architecture: specialized network with four convolutional blocks and dense layer for feature learning, and (4) Downstream Classification: multiple classifier evaluation using extracted features.

Our proposed self-supervised learning-based SAR ATR architecture is depicted in Figure 1.

### 3.2. Dataset Description and Preprocessing

Our experiments utilize the Synthetic and Measured Paired and Labeled Experiment (SAMPLE) dataset [3], a comprehensive SAR ATR benchmark designed to facilitate research on bridging the domain gap between synthetic and real-world SAR imagery. The dataset exhibits a paired structure where each measured (real) SAR image is accompanied by a corresponding synthetic image generated from high-fidelity computer-aided design (CAD) models. Our study focuses exclusively on measured imagery to evaluate SSL performance under realistic operational conditions without synthetic data dependency. The dataset contains ten military vehicle classes with both synthetic and measured SAR images for each vehicle. Synthetic data were generated using meticulously truthed CAD models closely matched to real vehicle images from the publicly available Moving and Stationary Target Acquisition and Recognition (MSTAR) dataset [33]. Images were collected at elevation angles between $14^{\circ }$ and $17^{\circ }$ and azimuth angles ranging from $10^{\circ }$ to $80^{\circ }$. The dataset was specifically created to facilitate research on bridging the gap between measured and synthetic SAR imagery for automatic target recognition applications.

We gathered the data from the publicly available GitHub repository [34], where the dataset has been made available. There are two different kinds of files in the repository: MATLAB files and PNG images. ‘decibel’ and ‘qpm’ are two distinct directories with images in PNG format that are accessible, and the images in both folders are identical. The actual measured images are available in one of the subfolders in each of these folders, while the corresponding synthetic image to each of the measured images is available in the other. Each image filename follows a standardized naming convention encoding parsable metadata: target class identifier, elevation angle ($14^{\circ }$, $15^{\circ }$, $16^{\circ }$, or $17^{\circ }$), azimuth angle ($10^{\circ }$ to $80^{\circ }$ at variable intervals), and data type (synthetic/measured). This paired structure enables one-to-one correspondence between synthetic and measured images for the same target at matching viewing geometries.

Our Study’s Data Usage Rationale: We utilize only measured (real) images from the SAMPLE dataset, totaling 1345 images across 10 classes, to evaluate SSL performance under authentic operational conditions without synthetic data augmentation. This choice is motivated by three considerations: (1) Realism: measured images contain authentic sensor noise, atmospheric effects, and target variability absent in synthetic data, (2) SSL evaluation: self-supervised learning should demonstrate effectiveness on limited real data, and (3) Operational relevance: deployed SAR ATR systems process real sensor acquisitions. Following [3], we adopt an elevation-based train-test split where all images at $17^{\circ }$ elevation (539 samples) serve as the held-out test set, while images at $14^{\circ }$, $15^{\circ }$, and $16^{\circ }$ elevations (806 samples) constitute the training pool. Table 1 reports the complete distribution. From the 1345 measured (real) SAR images across 10 military vehicle classes, we use only measured images, excluding synthetic pairs, to evaluate SSL under realistic operational conditions.

The preprocessing pipeline involves two key steps. First, intensity normalization is performed by scaling pixel values to a range of [0, 1] using min-max scaling, ensuring consistent intensity distributions across images. Second, spatial standardization is applied by center-cropping and resizing the images to 64×64 pixels, maintaining spatial consistency while preserving target details. We employ a systematic experimental protocol to ensure reproducible and reliable results. The experimental framework evaluates SSL approaches using the SAMPLE dataset with both held-out test data and rigorous cross-validation protocols to assess the performance reliability and generalizability.

Data Leakage Prevention Protocol: To ensure rigorous evaluation and prevent data leakage between training and testing phases, we implement a strict elevation-based data separation strategy. Following the SAMPLE dataset protocol [3], all images at a 17^∘^ elevation (539 test samples across 10 classes) are held out from both the SSL pretext training and downstream classifier training phases. Only images at 14^∘^, 15^∘^, and 16^∘^ elevation angles (806 training samples) are available for pretext task learning and downstream training. This elevation-based split ensures that test data remains completely unseen throughout the entire training pipeline, preventing any form of data leakage. Additionally, during downstream classifier training, we employ stratified train-validation splitting where 15% of the training data is reserved for validation, with class proportions preserved to maintain balanced representation. This approach ensures that validation performance accurately reflects generalization capability across all target classes, providing reliable early stopping criteria without introducing bias toward majority classes. This rigorous protocol combining held-out test elevation, proportionate validation, and consistent data partitioning across all *k*-value experiments substantiates that reported performance metrics represent genuine generalization to unseen data rather than memorization of training examples.

### 3.3. Multi-Task Pretext Learning Framework

#### 3.3.1. Two-Stage Hierarchical SSL Pipeline with Multi-Task Pretext Learning

Our framework implements a two-stage hierarchical SSL pipeline. Stage 1 (Pretext Training) learns general-purpose representations from unlabeled data through pretext tasks. Stage 2 (Downstream Evaluation) transfers these learned features to target classification by freezing the pretrained encoder and training only the downstream classifier. This hierarchical separation ensures that pretext task learning captures domain-invariant features without overfitting to specific downstream labels.

Within the first stage (pretext training), we employ multi-task learning where a single shared encoder is trained simultaneously on nine pretext tasks. Our multi-task pretext objective jointly optimizes all nine tasks through a unified loss function, which enables the network to learn a unified representation space that captures multiple complementary aspects of SAR imagery simultaneously. In the second stage (downstream evaluation), we freeze the pretrained encoder and extract 2048-length feature vectors for all training samples. These extracted features are then used to train various downstream classifiers for the target classification task.

We adopt multi-task learning within the pretext stage (rather than training nine separate single-task models) for some key advantages: (1) Training efficiency: requires 2–3 min for training the nine pretext tasks simultaneously for the SAMPLE dataset taken, (2) Feature complementarity: ablation studies confirm multi-task synergy yields better performance over best single-task performance through simultaneous gradient contributions from all tasks, and (3) Data efficiency: each of 806 images provides nine training signals (7254 effective examples), compensating for limited labeled data, with minimal computational overhead.

#### 3.3.2. Pretext Tasks Design and Theoretical Justification

We propose a comprehensive self-supervised learning framework comprising nine methodically constructed pretext tasks, each designed to exploit distinct structural characteristics inherent to synthetic aperture radar (SAR) imagery. Our approach is grounded in rigorous theoretical principles derived from established radar phenomenology and signal processing theory. The foundation of our methodology rests upon some key observations from the radar remote sensing literature. First, electromagnetic scattering mechanisms in SAR targets demonstrate invariance properties under specific geometric and radiometric transformations [35,36], providing a theoretical basis for transformation-based self-supervision. Second, operational SAR systems are inherently subjected to multiplicative speckle noise, atmospheric propagation effects, and system-induced artifacts [37], which require robust feature representations that can model these degradation processes. Third, SAR backscatter signatures exhibit multi-scale spatial dependencies arising from the complex interplay between target geometry and radar viewing parameters [38,39], motivating the development of hierarchical feature extraction mechanisms. Building upon these theoretical foundations, our pretext tasks design systematically addresses each aspect of SAR data complexity while maintaining computational scalability for large-scale applications. The pretext tasks can be classified into these broad categories:

Original Image Prediction ($T_{0}$): Classification of unmodified images serves as a baseline task and helps maintain original SAR signature characteristics.

Geometric Invariance Tasks ($T_{1}-T_{5}$): Based on the principle that SAR target signatures should be recognizable regardless of platform orientation or viewing geometry, these tasks force the network to learn rotation and reflection-invariant features essential for multi-aspect target recognition. These tasks include:

- 90^∘^ Rotation ($T_{1}$): Rotates the image counterclockwise by 90^∘^, leveraging viewpoint-invariant nature of SAR targets.
  (1) $$R_{90}\left(x\right)\;=\;\mathrm{rot}90\left(x,k=1\right)$$
- 180^∘^ Rotation ($T_{2}$): Rotation by 180^∘^, helping to learn orientation-invariant features.
  (2) $$R_{180}\left(x\right)\;=\;\mathrm{rot}90\left(x,k=2\right)$$
- 270^∘^ Rotation ($T_{3}$): Counterclockwise rotation by 270^∘^ (equivalent to 90^∘^ clockwise), helping rotational feature learning for multi-aspect recognition.
  (3) $$R_{270}\left(x\right)\;=\;\mathrm{rot}90\left(x,k=3\right)$$
- Horizontal Flip ($T_{4}$): Flips the image horizontally, creating a mirrored version to learn geometric invariances.
  (4) $$F_{h}\left(x\right)\;=\;\mathrm{flip}\left(x,\mathrm{axis}=\mathrm{width}\right)$$
- Vertical Flip ($T_{5}$): Flips the image vertically, producing an upside-down version, enhancing robustness to target orientation changes.
  (5) $$F_{v}\left(x\right)\;=\;\mathrm{flip}\left(x,\mathrm{axis}=\mathrm{height}\right)$$

Signal Quality Robustness Tasks ($T_{6}-T_{7}$): Motivated by operational requirements where SAR imagery may be degraded by atmospheric effects, sensor noise, or processing artifacts. These tasks ensure the learned representations are robust to signal quality variations commonly encountered in operational scenarios. These transformations include:

- Denoising ($T_{6}$): The network learns to identify images processed with Gaussian smoothing (implemented as “denoising” task). This applies a 3 × 3 Gaussian kernel with fixed standard deviation σ=0.5, which reduces noise and high-frequency details in SAR imagery.
  (6) $$S\left(x\right)\;=\;x*G_{\sigma =0.5}$$
  where $G_{\sigma =0.5}$ is a 3 × 3 Gaussian kernel with standard deviation 0.5.
- Blur Prediction ($T_{7}$): The network applies Gaussian blur transformations with a 5 × 5 kernel, simulating atmospheric effects and resolution variations. The standard deviation is chosen randomly between 0.5 and 1.0 to create varying levels of blur effect.
  (7) $$B\left(x\right)\;=\;x*G_{\sigma_{b}}$$
  where $G_{\sigma_{b}}$ is a 5 × 5 Gaussian kernel with standard deviation $\sigma_{b}∼U\left(0.5,1.0\right)$.

Multi-Scale Analysis Task ($T_{8}$): To address the multi-resolution nature of SAR phenomenology, where target features manifest at different spatial scales depending on sensor parameters and target geometry, we implement a zoom-in transformation. The network predicts zoom-in transformations that upscale the image by a factor between 1.2 and 1.5, then crops the center region back to the original size, creating a zoom effect. This corresponds to 20% to 50% zoom levels, enabling multi-scale feature learning important for variable resolution scenarios.

(8) Z(x)=CenterCrop(Resize(x,s·size(x)),size(x))

where s∼U(1.2,1.5) is the scaling factor.

**Figure 2:**
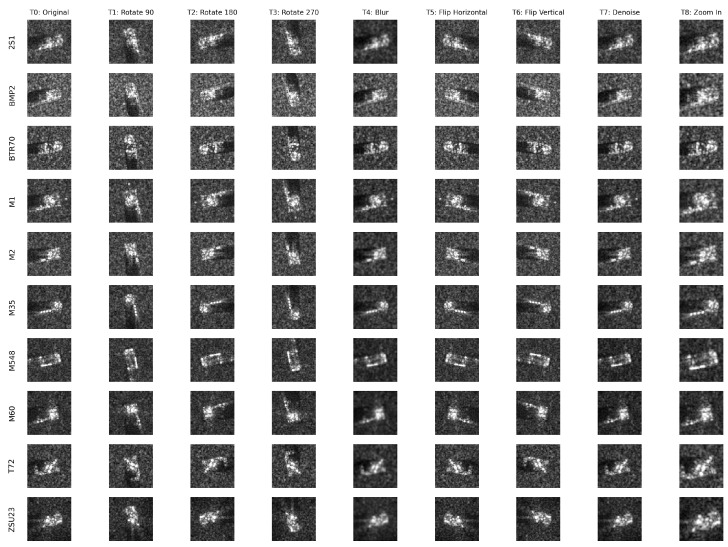
Visual demonstration of all nine pretext transformations ($T_{0}$ to $T_{8}$) applied to representative SAR images from each target class. Each row shows a different vehicle class, while each column represents a specific transformation.

Figure 2 illustrates the complete set of pretext transformations applied to representative samples from each target class in our dataset.

#### 3.3.3. Pretext Network Architecture

Our CNN architecture is specifically designed to capture the characteristics of SAR imagery, following the implementation of Lewis et al. [3] to ensure fair comparison and reproducibility. The network employs progressive channel expansion (16 → 32 → 64 → 128) to enable feature learning. Spatial resolution is systematically reduced using max-pooling operations that preserve essential spatial relationships while lowering computational complexity. The pretext classifier consists of a fully connected head (2048 → 1000 → 500 → 250 → *N*) with ReLU activations, where *N* denotes the number of pretext transformation classes. All convolutional layers use 3×3 kernels with same-padding to preserve spatial dimensions prior to pooling. Pretext training is formulated as a single-label classification problem over the *N* transformation classes and is optimized using the categorical cross-entropy loss

$$L_{\mathrm{pretext}}\;=\;L_{CE}\left(p,y\right),$$

where *p* represents the predicted probability distribution over the transformation classes and *y* is the corresponding ground-truth label. Optimization is performed using the Adam optimizer (learning rate =0.001, batch size =16) with early stopping (patience =5 epochs) to mitigate overfitting and encourage generalizable representations for downstream tasks. The architecture is shown in Table 2.

Computational Resources: Pretext training requires approximately 2–3 min on a single GPU (NVIDIA GeForce RTX 3090, Santa Clara, CA, USA ) for the complete SAMPLE dataset. Feature extraction for all downstream classifiers adds an average of 15.5 ms per 64×64 image. The framework is designed for computational efficiency, making it practical for research and operational deployment scenarios.

### 3.4. SimCLR Baseline Implementation

To establish a rigorous comparison with established SSL methods, we implement SimCLR (Simple Framework for Contrastive Learning of Visual Representations) [30] as a strong baseline, following the original paper’s methodology as closely as possible while making necessary adaptations for SAR imagery and our experimental conditions. SimCLR represents a fundamentally different SSL paradigm compared to our task-based approach, while our method employs explicit pretext tasks. SimCLR learns representations through instance discrimination, where the model learns to distinguish between different images while recognizing augmented views of the same image as equivalent.

Our implementation faithfully replicates the core architectural and algorithmic components of the original SimCLR paper [30]. We preserve the fundamental two-phase training protocol (contrastive pretraining followed by linear evaluation), the exact projection head architecture with batch normalization, the NT-Xent loss formulation with cosine similarity, and the linear evaluation protocol for representation quality assessment. We employ LARS optimizer with learning rate scaling ($\mathrm{lr}\;=\;0.3\times \frac{\mathrm{batch}\;\mathrm{size}}{256}$), linear warmup for the first 10 epochs, and cosine decay learning rate scheduling. The temperature parameter (τ=0.5), weight decay ($10^{-6}$), momentum (0.9), and LARS trust coefficient (0.001) are set exactly as recommended in the original work. This faithful implementation ensures our comparison reflects the true performance characteristics of SimCLR rather than implementation-specific variations.

The SimCLR framework consists of two main phases. During contrastive pretraining, each SAR image *x* undergoes random augmentation to generate two correlated views $\left(x_{i},x_{j}\right)$. For each image, we randomly select two different augmentations from our SAR-adapted transformation set ($T_{0}$–$T_{8}$). Each view applies exactly one randomly selected transformation, with the constraint that the two views use different transformations to ensure meaningful view diversity while preserving target-specific SAR signatures.

Both augmented views are processed through a shared ResNet-18 encoder f(·) to extract 512-dimensional feature representations. Following the original SimCLR protocol exactly, we employ a two-layer MLP projection head g(·) with the architecture specified in the original paper: Linear (512→2048) → BatchNorm → ReLU → Linear (2048→128). This produces 128-dimensional normalized embeddings z=g(f(x)) for contrastive learning. The projection head, including batch normalization between layers as specified in the original work, is crucial for effective contrastive learning but is discarded during downstream evaluation.

The contrastive loss encourages agreement between positive pairs (augmented views of the same image) while maximizing disagreement with negative pairs (views from different images). For a batch of *N* images producing 2N augmented views, we apply the normalized temperature-scaled cross-entropy (NT-Xent) loss exactly as defined in the original paper. For a positive pair $\left(z_{i},z_{j}\right)$:

(9) $$L_{i,j}\;=\;-\log\frac{\exp(\mathrm{sim}\left(z_{i},z_{j}\right)/\tau )}{\sum_{k=1}^{2N}1_{\left[k\neq i\right]}\exp\left(\mathrm{sim}\left(z_{i},z_{k}\right)/\tau \right)}$$

where $\mathrm{sim}(z_{i},z_{j})$ denotes cosine similarity between L2-normalized embeddings, τ=0.5 is the temperature parameter (original paper’s recommended value), and k≠i excludes self-comparisons. The final loss averages over all 2N positive pairs in the batch, precisely following the original implementation.

We replicate SimCLR’s training protocol with dataset-appropriate adaptations. Following the original paper’s optimizer recommendations, we use LARS (Layer-wise Adaptive Rate Scaling) with base learning rate 0.3, momentum 0.9, weight decay $10^{-6}$, and trust coefficient 0.001. The learning rate is scaled by batch size: lr=0.3×(batch size/256), maintaining the original paper’s linear scaling rule. We implement the original paper’s learning rate schedule: linear warmup for the first 10 epochs, followed by cosine decay to zero over the remaining epochs. The original SimCLR employs batch sizes of 256–8192 to provide sufficient negative samples; for our dataset constraints, we use an adaptive batch sizing strategy where batch size is set to $\min(64,\left\lfloor N_{\mathrm{train}}/2\right\rfloor )$ to ensure at least two batches per epoch while maximizing negative sample diversity. This adaptation is necessary for low *k*-values where training samples are limited, but preserves the core contrastive learning principle. We train for 200 epochs, consistent with one of the pretraining durations explored in the original SimCLR paper.

The second phase, linear evaluation, follows the standard protocol established by the original SimCLR paper. We freeze the trained ResNet-18 encoder and train only a single linear layer (512-length input to 10 target classes) using supervised labels and cross-entropy loss. This evaluation protocol, widely adopted in the SSL literature, tests whether the encoder has learned features that are linearly separable for the downstream task, providing a fair comparison between SSL methods. Following the original paper’s specifications, we train the linear classifier for 100 epochs using SGD with learning rate 0.1, momentum 0.9, weight decay 0.0, and early stopping (patience = 10 epochs) on validation loss.

We modified ResNet-18’s initial convolutional layer to accept grayscale input (1 channel instead of 3) to adapt the SAR data we used. Our adaptive batch sizing ensures stable contrastive learning even with limited training data at low *k*-values, preventing training failures while maintaining meaningful negative sampling.

### 3.5. Downstream Classification Approaches

To evaluate the quality of learned representations from our SSL framework, we conduct comprehensive downstream classification experiments using both traditional machine learning and deep learning approaches. All classifiers operate on feature vector of length 2048 that is extracted from the pre-trained SSL encoder network.

#### 3.5.1. Traditional Machine Learning Classifiers

We implement four well-established machine learning algorithms, each configured following standard practices and widely used default hyperparameter settings for high-dimensional feature classification.

Support Vector Machine (SVM): We employ an SVM with linear kernel optimized for high-dimensional features commonly encountered in deep learning representations. The implementation utilizes scikit-learn’s SVC with probability estimation enabled for comprehensive performance analysis.

XGBoost: Our XGBoost configuration implements an ensemble of 100 decision trees. The classifier is configured for multi-class soft probability prediction with 10 output classes and automatic label encoding disabled.

Random Forest: The Random Forest classifier employs 100 estimators. Bootstrap sampling and other ensemble parameters maintain default scikit-learn configurations.

Gradient Boosting: The Gradient Boosting implementation utilizes 100 estimators. The implementation follows scikit-learn’s gradient boosting approach with learning rate, maximum tree depth, and other regularization parameters configured according to the hyperparameter optimization results to prevent overfitting while maintaining classification performance.

#### 3.5.2. Deep Learning Architectures

We evaluate several state-of-the-art deep neural network architectures, each adapted for SAR imagery classification.

ResNet18: We implement a modified ResNet18 architecture that can handle both the image and the input of the feature vector. The implementation includes adaptive input processing to accommodate 2048-length feature vectors by reshaping them into spatial representations suitable for convolutional processing.

U-Net: We adapt the U-Net encoder-decoder architecture for classification tasks by incorporating global average pooling in the decoder bottleneck. The encoder extracts hierarchical features, while skip connections preserve fine-grained spatial information, making this architecture particularly suitable for SAR imagery, where spatial context is crucial for target recognition.

MobileNet variants (v1 with different width multipliers: 1.0, 0.75, 0.5, 0.25): We implement the MobileNet v1 architecture with different width multipliers to explore the trade-offs between computational efficiency and classification performance. The architecture employs depthwise separable convolutions to reduce computational cost while maintaining feature extraction capability.

EfficientNet variants (B0, B1, B2, B3): Our EfficientNet implementation leverages compound scaling to systematically balance network depth, width, and resolution. We evaluated EfficientNet-B0 through B3 variants. The balanced scaling approach of EfficientNet makes it particularly suitable for SAR applications where computational efficiency is crucial while maintaining high accuracy requirements.

GAN Classifier: We implement a discriminator-based classification approach that leverages generative adversarial network (GAN) principles for target recognition. The GAN classifier consists of a discriminator network trained to distinguish between different target classes rather than real versus synthetic data. This approach utilizes the representational power of adversarial training to learn robust feature discriminations.

CNN: Additionally, we have employed the CNN architecture that is used to train the pretext features. We have adapted the CNN architecture to process one-dimensional feature vector while keeping the architecture parameters the same. For downstream classification, we have used 1D convolutions and filters instead of 2D.

Table 3 provides the hyperparameter specifications used to implement the downstream classifiers.

### 3.6. Experimental Design and Evaluation Methodology

#### 3.6.1. Evaluation Metrics

- Accuracy: $A\;=\;\frac{TP+TN}{TP+TN+FP+FN}$, representing the overall percentage of correctly classified samples and providing a general measure of classifier effectiveness.
- Precision: $P\;=\;\frac{TP}{TP+FP}$, macro-averaged across all classes to account for potential class imbalance effects.
- Recall: $R\;=\;\frac{TP}{TP+FN}$, macro-averaged to ensure equal consideration of all target classes regardless of frequency.
- F1-Score: $F1\;=\;\frac{2PR}{P+R}$, representing the harmonic mean of precision and recall to provide a balanced performance measure.
- Area Under the Curve (AUC): $AUC\;=\;\int_{0}^{1}TPR\left(FPR^{-1}\left(t\right)\right)\;dt$, providing a threshold-independent measure of classifier discriminative ability across all possible decision boundaries.
- True Positive Rate at Fixed False Positive Rates: $TPR\;=\;\frac{TP}{TP+FN}$ evaluated at strategically chosen FPR thresholds of 1%, 5%, 10%, 15%, and 20% to assess performance under varying operational constraints typical in SAR target recognition applications.
- Pretext Training Time: Total wall-clock time required for SSL pretext tasks training, measured across multiple epochs until convergence.
- Downstream Classifier Training Time: Training duration for each downstream classifier using extracted feature representations.

#### 3.6.2. Data Availability Scenarios and *k*-Value Selection

Our experimental framework evaluates performance across varying fractions of measured training data, denoted as *k* ranging from 0.05 to 1.00, where *k* represents the percentage of available measured (real) training data used for both pretext and downstream classifier training. The *k* parameter constitutes a critical experimental design element that enables systematic assessment of SSL framework effectiveness under different data availability constraints commonly encountered in operational SAR ATR scenarios. The significance of *k*-value analysis extends beyond academic evaluation, providing essential guidance for operational deployment decisions where data acquisition costs, labeling requirements, and rapid deployment constraints directly impact system viability.

While our framework supports comprehensive evaluation across all *k*-values for traditional machine learning and most deep learning architectures, generative adversarial network (GAN) based classifiers require special consideration at extremely low *k*-values. At *k* = 0.05 (5% of training data), the available sample size (43 training samples from the SAMPLE dataset) falls below the minimum requirements for stable GAN training. GANs require adequate sample diversity for stable adversarial training to ensure numerical stability and avoid training failures. GANs exhibit several critical issues, including numerical instability due to insufficient batch statistics for BatchNorm layers, gradient instability leading to mode collapse and vanishing gradients, and training convergence failure. These technical limitations align with established findings in GAN literature [40,41], where minimum dataset requirements are necessary for stable training. Consequently, our evaluation presents GAN-based classifier results for k≥0.10 (at least 85 training samples).

The *k*-value methodology provides valuable insights into the relationship between data availability and model performance across diverse architectural families. This systematic approach enables identification of operational thresholds where specific architectures become viable, quantification of performance degradation under data constraints, and optimization of resource allocation for data collection efforts. For operational SAR ATR systems, *k*-value analysis directly informs critical deployment decisions, including minimum data collection requirements, expected performance under constrained scenarios, and cost-benefit analysis of additional data acquisition investments. The distribution of data for each *k* (fraction of real data used) is shown in Table 4.

#### 3.6.3. Cross-Validation Protocol

To ensure robust and generalizable results, we also implement a rigorous cross-validation protocol. The cross-validation experiments validate our findings using independent data splits and provide confidence intervals for performance estimates.

Addressing the inherent data scarcity challenges in SAR ATR applications, our cross-validation methodology employs a conservative approach with reduced data usage to better reflect real-world operational constraints. We implement a 5-fold stratified cross-validation protocol using exclusively real SAR imagery, selecting the first 92 images per class lexicographically to ensure reproducible dataset composition and eliminate potential selection bias. This results in a total dataset of 920 images (92×10 classes).

The cross-validation architecture differs fundamentally from our *k*-value experimental framework in several key aspects: (1) Data Volume: Cross-validation uses 920 real images compared to the full dataset that includes the original train and the test set of 1345 images, representing the evaluation under data-scarce conditions; (2) Pretext Training: All 920 images are used for self-supervised pretext training without fold splits, ensuring the SSL feature extractor learns from the complete available dataset; (3) Downstream Evaluation: The same 920 images are subjected to 5-fold cross-validation where each fold uses approximately 736 images for training and 184 for testing, with 25% of training data reserved for validation; (4) Statistical Rigor: This protocol provides confidence intervals and standard deviations across folds, offering more reliable performance estimates than single train-test splits used in the main *k*-value experiments.

## 4. Experimental Results and Analysis

### 4.1. Overall Performance Analysis

Our comprehensive experimental evaluation encompasses 16 diverse downstream classifier architectures systematically evaluated across four primary computational paradigms: traditional machine learning (SVM, Random Forest, XGBoost, Gradient Boosting), convolutional neural networks (CNN, ResNet18, U-Net), efficient architectures (MobileNet variants with width multipliers 1.0, 0.75, 0.5, 0.25; EfficientNet variants B0–B3), and generative adversarial networks (GAN classifier). This systematic evaluation across values of *k* (from 0.05 to 1.0) provides comprehensive insights into SSL framework performance under diverse data availability constraints, generating 320 experimental configurations that thoroughly validate our approach across operational scenarios typical of real-world SAR ATR deployments.

The experimental results reveal striking performance patterns that fundamentally challenge conventional assumptions about deep learning superiority in computer vision tasks. Traditional machine learning approaches consistently demonstrate exceptional performance, with SVM achieving a remarkable 99.63% accuracy, 99.63% precision, and 99.66% recall at full training data availability (*k* = 1.0), accompanied by near-perfect ROC AUC scores of 1.0000 for all target classes. Random Forest maintains robust second-best performance with 99.26% accuracy, 99.30% precision, and 99.22% recall, achieving ROC AUC values between 0.9990 and 1.0000 across all target classes. XGBoost delivers competitive 93.88% accuracy with consistently high per-class discrimination above 0.9930. These results establish traditional ML methods as the optimal choice for SSL-based SAR ATR applications, contradicting the common bias toward deep neural architectures in computer vision research.

Among deep learning architectures, ResNet18 emerges as the top performer with 97.40% accuracy at *k* = 1.0, achieving exceptional per-class ROC AUC values between 0.9960 and 1.0000 across classes, demonstrating robust discrimination across all target types. CNN provides competitive baseline performance at 94.25% accuracy, while U-Net achieves 84.04% accuracy despite higher computational requirements. The efficient architecture family demonstrates compelling performance-efficiency trade-offs, with EfficientNet B3 reaching peak efficient architecture performance at 95.92% accuracy and per-class ROC AUC values between 0.9733 and 1.0000, EfficientNet B2 achieving 89.42%, EfficientNet B1 attaining 95.18%, and EfficientNet B0 delivering baseline 92.95% accuracy.

MobileNet variants showcase scalability across computational constraints with remarkable performance. Standard MobileNet achieves an exceptional 98.70% accuracy with per-class ROC AUC values ranging from 0.9995 to 1.0000. MobileNet 0.75 reaches 97.77%, MobileNet 0.5 attains 95.73%, and MobileNet 0.25 delivers 92.21% acccuracy. GAN-based classifiers demonstrate promising generative learning capabilities with 96.85% accuracy and consistently high per-class discrimination above 0.9785, validating the framework’s applicability to adversarial learning paradigms while maintaining competitive performance across diverse target classes.

### 4.2. Data Availability Impact and k-Value Analysis

The *k*-value experimental design provides critical insights into SSL framework robustness under varying data constraints, systematically evaluating performance degradation and recovery patterns across data availability scenarios from extreme scarcity (*k* = 0.05, 5% of training data with only 43 total samples) to full availability (*k* = 1.0, 100% with 806 samples). Our systematic evaluation reveals dramatic performance variations that directly inform operational deployment strategies across diverse resource-constrained environments typical of real-world SAR ATR applications.

At extreme data scarcity (*k* = 0.05), traditional machine learning approaches demonstrate remarkable resilience that far exceeds random chance performance. SVM achieves 51.95% accuracy under these severe constraints with per-class ROC AUC values ranging from 0.7015 to 0.9970, while Random Forest maintains 46.01% accuracy with consistent discrimination above 0.5385 across all target classes. These performance levels remain operationally relevant for preliminary target screening applications, demonstrating the framework’s capability to extract meaningful features even from minimal training data. In contrast, deep learning architectures exhibit significant performance degradation, with ResNet18 achieving only 24.86% accuracy and CNN reaching 27.27% accuracy at *k* = 0.05.

**Figure 3:**
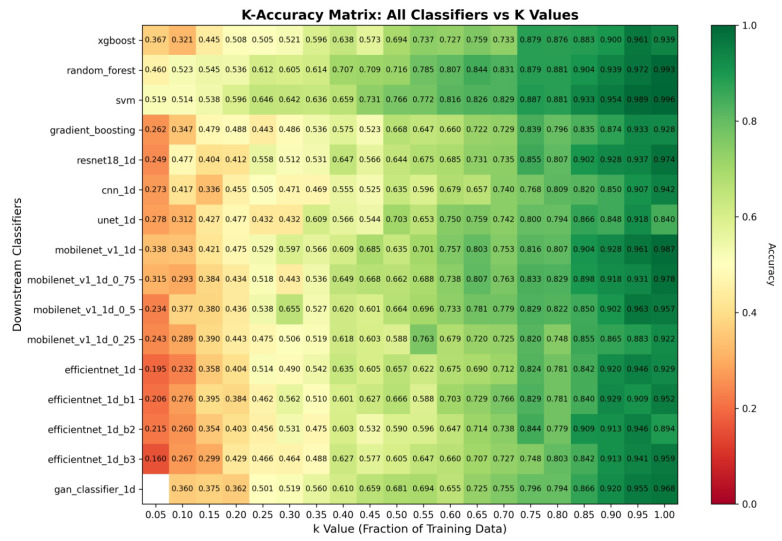
Comprehensive performance evaluation of different downstream classifiers in varying data availability scenarios.

**Figure 4:**
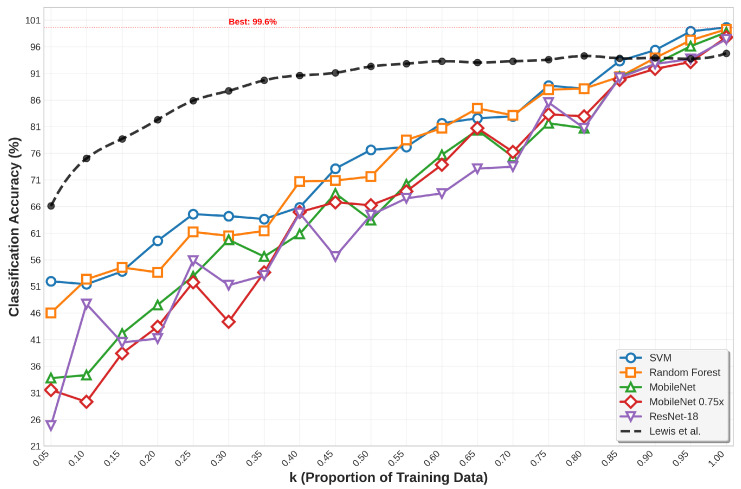
Learning curves showing accuracy evolution of top 5 classifiers across different training data fractions (*k* = 0.05–1.00). Each colored line represents a classifier’s performance trajectory.

The performance trajectory from *k* = 0.05 to *k* = 1.0 reveals distinct architectural families’ sensitivity to data availability. Traditional ML methods exhibit graceful degradation under data constraints while maintaining consistent relative performance across all *k*-values, with SVM improving from 51.95% to 99.63% accuracy and Random Forest advancing from 46.01% to 99.26%. Deep learning architectures show higher variance at low *k*-values but demonstrate rapid improvement as data availability increases: ResNet18 advancing from 24.86% accuracy at *k* = 0.05 to 97.40% at *k* = 1.0, and CNN improving from 27.27% to 94.25%. EfficientNet variants display particularly interesting scaling behavior, with B3 improving from 15.96% at *k* = 0.05 to 95.92% at *k* = 1.0, while B0 advances from 19.48% to 92.95%, indicating architectural depth’s relationship to data requirements. The accuracy on the full test set in varying training data availability scenarios is shown in Figure 3, while the accuracy curves with different *k* values for top downstream classifiers are depicted in Figure 4.

### 4.3. Computational Efficiency and Operational Metrics

The timing analysis reveals highly favorable computational characteristics essential for operational deployment across diverse hardware configurations and real-time processing requirements. SSL feature extraction from the trained pretext model took a computational cost of 15.35 ms per image. Inference times with the extracted feature vary dramatically across architectural families, enabling precise performance-efficiency optimization. Traditional ML methods achieve exceptional computational efficiency: Gradient Boosting requires only 0.01 ms per image, XGBoost needs 0.02 ms per image. Random Forest demands 0.05 ms per image, and SVM requires 0.08 ms per image, despite its superior accuracy. Deep learning architectures require 0.05–0.37 ms per image, with ResNet18 achieving a competitive 0.13 ms per image, CNN requiring 0.05–0.06 ms per image, and U-Net demanding 0.35–0.37 ms per image due to its complex encoder-decoder architecture.

Efficient architectures maintain competitive timing profiles optimized for resource-constrained deployments. EfficientNet variants require 0.16–0.28 ms per image across B0–B3 configurations, with B0 achieving 0.16 ms per image and B3 reaching 0.27–0.28 ms per image, demonstrating excellent scalability. MobileNet variants need 0.05–0.11 ms per image depending on the width multiplier, with the 0.25 width multiplier variant achieving a fast 0.09 ms per image while maintaining 92.21% accuracy. GAN classifiers provide balanced performance at 0.08–0.09 ms per image, making them suitable for specialized applications requiring generative model capabilities.

Total processing times range from 15.36 ms (Gradient Boosting: 15.35 ms feature extraction and 0.01 ms inference) to 15.72 ms (U-Net: 15.35 ms feature extraction and 0.37 ms inference), enabling real-time processing capabilities for operational SAR ATR systems. The minimal variance in total processing time (±0.36 ms) across architectural families confirms that feature extraction dominates computational cost, making downstream classifier selection primarily a performance consideration rather than a computational constraint. This characteristic enables deployment flexibility where high-accuracy classifiers like SVM (99.63% accuracy, 15.43 ms total) can be deployed when computational resources permit, while fast alternatives like Gradient Boosting (92.76% accuracy, 15.36 ms total) provide viable options for extreme real-time constraints or battery-powered platforms.

### 4.4. Per-Class Performance Analysis

Class-wise ROC AUC analysis reveals exceptional discrimination capability across all target types, with particularly strong performance for specific vehicle classes that demonstrate distinct feature characteristics in the SSL representation space. The per-class analysis provides critical insights into target-specific recognition capabilities and potential operational deployment considerations for diverse mission requirements.

SVM achieves near-perfect per-class discrimination with ROC AUC values demonstrating exceptional consistency. This performance demonstrates the SSL framework’s ability to learn discriminative features for diverse target types, from tracked tanks to wheeled vehicles. Random Forest maintains robust per-class performance with ROC AUC values ranging from 0.9990 to 1.0000 across all target classes. The minimal performance variation indicates consistent feature quality across vehicle types. XGBoost demonstrates reliable per-class discrimination with ROC AUC values consistently above 0.9941. ResNet18 exhibits competitive per-class discrimination with ROC AUC values between 0.9974 and 1.0000, achieving perfect discrimination for Classes 5 (M35) and 6 (M548).

Class-specific insights reveal interesting patterns in target discrimination. Class 6 (M548 tracked carrier) exhibits perfect discrimination (ROC AUC = 1.0000) across all top-performing classifiers (SVM, Random Forest, XGBoost), indicating highly distinctive SSL-learned features that separate this tracked utility vehicle from combat platforms. Classes 3 (M1 tank) and 5 (M35 truck) consistently achieve perfect or near-perfect discrimination, suggesting that main battle tanks and wheeled logistics vehicles possess easily distinguishable radar signatures in the learned feature space. The minimal interclass performance variation (typically within 0.001–0.002 ROC AUC) across top classifiers demonstrates the SSL framework’s balanced feature learning capability for diverse military vehicle types encountered in operational SAR ATR scenarios.

### 4.5. Operational Performance Thresholds

The analysis of True Positive Rate (TPR) at fixed False Positive Rate (FPR) thresholds provides operationally critical performance metrics that directly inform deployment decisions across diverse mission requirements and operational constraints. These metrics represent real-world performance under varying tolerance levels for false alarms, allowing precise system configuration for specific operational scenarios from high-precision reconnaissance to rapid threat assessment.

At the stringent 1% FPR threshold, representing high-precision applications where false alarms carry significant operational costs, SVM achieves an exceptional 99.89% TPR, demonstrating near-perfect target detection with minimal false alarm rates suitable for critical decision-making scenarios. Random Forest attains 99.65% TPR at this threshold, while XGBoost reaches 96.10% TPR, and ResNet18 achieves 98.70% TPR. These performance levels indicate excellent discrimination capability suitable for high-stakes applications such as threat identification in densely populated areas or precision targeting scenarios where misclassification consequences are severe.

At the moderate 5% FPR threshold, providing operational flexibility for scenarios tolerating moderately higher false alarm rates in exchange for enhanced target detection, perfect 100% TPR is achieved by SVM, Random Forest (100%), XGBoost (100%), and ResNet18 (99.26%). MobileNet standard achieves perfect 100% TPR, while EfficientNet B3 reaches 99.44% and Gradient Boosting attains 97.40%. This threshold represents optimal operating points for many operational scenarios where some false alarms are acceptable to ensure comprehensive target detection.

The 10% FPR threshold demonstrates robust detection performance under permissive operational constraints. Perfect 100% TPR is achieved by SVM, XGBoost, ResNet18, MobileNet standard, and multiple EfficientNet variants. CNN reaches 98.33%, U-Net achieves 97.96%, and even challenging scenarios maintain TPR above 94%. The consistent achievement of 100% TPR at this threshold across top-tier classifiers demonstrates robust detection performance suitable for surveillance applications where comprehensive area monitoring is prioritized over precision.

At higher FPR thresholds (15% and 20%), all classifiers achieve TPR values above 98%, with many reaching perfect 100% detection. SVM maintains perfect 100% TPR at both 15% and 20% FPR thresholds, as do Random Forest, XGBoost, and most deep learning architectures. This performance consistency across varying operational constraints validates the SSL framework’s robustness and provides confidence for deployment across diverse mission requirements.

Operational deployment guidelines emerge from threshold analysis. For high-precision applications requiring minimal false alarms (≤1% FPR), SVM provides optimal performance, making it ideal for critical identification tasks, precision targeting, or operations in sensitive areas where false positives have severe consequences. For balanced operational requirements (5% FPR), multiple classifiers achieve perfect or near-perfect TPR, enabling selection based on computational constraints, with SVM and Random Forest providing optimal accuracy while Gradient Boosting offers fast processing for time-critical applications. For surveillance and area monitoring scenarios tolerating higher false alarm rates (≥10% FPR), the framework provides exceptional flexibility with multiple high-performance options enabling deployment optimization based on hardware limitations, power constraints, or specific mission requirements rather than accuracy considerations.

### 4.6. Cross-Validation Results and Statistical Validation

Our rigorous cross-validation methodology employs a conservative 5-fold class-proportionate approach using exclusively real SAR imagery. To ensure reproducible dataset composition and eliminate potential selection bias, the first 92 images per class are selected lexicographically. This results in a total dataset of 920 images (92×10 classes), representing a constrained data regime characteristic of operational SAR ATR scenarios, where labeled training data are inherently limited and costly to acquire. Unlike the main *k*-value experiment, this cross-validation setup uses the full 920-image dataset for SSL pretext training while subjecting the same data to a rigorous 5-fold class-proportionate partitioning for downstream classification evaluation.

The cross-validation results demonstrate exceptional consistency and statistical significance across all evaluated classifiers, providing robust validation of our main experimental findings. SVM maintains the best and most consistent performance with 99.13% mean accuracy cross folds, indicating exceptional stability across different data partitions and confirming its position as the optimal classifier for SSL-based SAR ATR. The narrow confidence interval demonstrates statistical significance of performance differences between methods and validates the reliability of SVM’s superiority across diverse data configurations.

Random Forest achieves a robust 96.96% mean accuracy, while ResNet18 delivers a competitive 96.52% mean performance among deep learning approaches. The cross-validation results for additional architectures demonstrate consistent performance patterns: XGBoost reaches 95.65% accuracy, Gradient Boosting achieves 94.13%, and efficient architectures maintain their relative performance hierarchies observed in the experiments. CNN attains 90.22% accuracy, U-Net reaches 87.93%, and MobileNet variants achieve 93.37–95.54% accuracy depending on width multiplier configuration.

Key findings from cross-validation analysis provide compelling evidence for framework robustness. First, consistent high performance across multiple classifiers (99.13% for SVM, 96.96% for Random Forest, 96.52% for ResNet18) indicates results are not attributable to classifier-specific optimization but reflect genuine feature quality from our SSL framework. Second, low standard deviations across all experiments demonstrate reproducibility and reliability, confirming that performance variations are minimal across different data partitions. Third, traditional machine learning approaches exhibit exceptional stability compared to deep learning models while maintaining competitive mean performance, reinforcing their suitability for operational deployment where consistency is crucial. Key findings from cross-validation analysis provide compelling evidence for framework robustness. First, consistent high performance across multiple classifiers (99.13% for SVM, 96.96% for Random Forest, 96.52% for ResNet18) indicates results are not attributable to classifier-specific optimization but reflect genuine feature quality from our SSL framework. Second, low standard deviations across all experiments demonstrate reproducibility and reliability, confirming that performance variations are minimal across different data partitions. Third, traditional machine learning approaches exhibit exceptional stability compared to deep learning models while maintaining competitive mean performance, reinforcing their suitability for operational deployment where consistency is crucial.

The cross-validation framework validates that results generalize reliably across different data splits, providing confidence in deployment scenarios where data composition may vary from training conditions. The consistent performance patterns between main *k*-value experiments and cross-validation results demonstrate framework robustness and validate our SSL approach’s effectiveness across diverse experimental protocols, supporting the reliability of our findings for real-world SAR ATR applications where data availability and composition constraints are common operational challenges.

### 4.7. Comprehensive Architecture Performance Analysis

The systematic evaluation across 16 downstream classifier architectures reveals distinct performance patterns that inform optimal deployment strategies. Traditional ML classifiers demonstrate remarkable consistency: SVM achieves 99.63% accuracy with exceptional precision (99.63%) and recall (99.66%), Random Forest maintains 99.26% accuracy with balanced precision (99.30%) and recall (99.22%), XGBoost delivers competitive 93.88% accuracy, and Gradient Boosting provides rapid inference time of 0.01 ms with 92.76% accuracy.

Deep learning architectures exhibit varied performance characteristics. CNN achieves 94.25% accuracy with moderate computational requirements (0.06 ms inference on extracted features), ResNet18 delivers top deep learning performance at 97.40% accuracy (0.13 ms inference on extracted features), and U-Net provides specialized capabilities with 84.04% accuracy despite higher computational cost (0.37 ms inference on extracted features). The efficient architecture family demonstrates compelling performance-efficiency trade-offs: EfficientNet B3 reaches peak efficient performance at 95.92% accuracy, EfficientNet B2 achieves 89.42%, EfficientNet B1 attains 95.18%, and EfficientNet B0 delivers baseline 92.95% performance.

MobileNet variants showcase scalability across computational constraints: standard MobileNet achieves exceptional 98.70% accuracy, MobileNet 0.75 reaches 97.77%, MobileNet 0.5 attains 95.73%, and MobileNet 0.25 delivers 92.21%. GAN-based classifiers demonstrate promising generative learning capabilities with 96.85% accuracy, validating the framework’s applicability to adversarial learning paradigms.

### 4.8. Contrastive Learning Baseline: SimCLR Performance Analysis

To establish a rigorous comparison with established contrastive learning methods, we evaluated SimCLR under identical experimental conditions with the same dataset, and *k*-value range 0.05–1.00). SimCLR represents a fundamentally different SSL paradigm: while our multi-task approach learns through explicit transformation classification, SimCLR employs instance discrimination with contrastive loss, making this comparison particularly informative for understanding optimal SSL strategies for SAR ATR.

The SimCLR baseline achieves 92.02% accuracy at full training data availability (*k* = 1.00), demonstrating that contrastive learning produces viable representations for SAR target recognition. However, this performance falls substantially short of our multi-task SSL framework, which achieves 99.63% accuracy (SVM), 99.26% (Random Forest), and 97.40% (ResNet18) under identical conditions. The performance gap persists across all *k*-values, with SimCLR reaching only 15.77% accuracy at *k* = 0.05 compared to 51.95% for our approach. Comprehensive performance metrics, including accuracy, precision, recall, and F1-score across all *k*-values are provided in Appendix A Table A5 (SimCLR Performance Metrics). Results reveal that SimCLR requires approximately k≥0.40 (327 training samples) to achieve performance comparable to our method at *k* = 0.25 (203 samples), indicating superior data efficiency for task-based SSL in the SAR domain.

The performance differential can be attributed to several domain-specific factors. First, SAR imagery exhibits structured transformations (rotations, flips) that are semantically meaningful for target recognition, making explicit task-based learning more effective than generic instance discrimination. Second, contrastive learning relies heavily on negative sampling diversity, requiring large batch sizes for optimal performance [42]; our SimCLR adaptation uses batch size 64 due to dataset constraints, potentially limiting contrastive signal strength. Third, the limited visual variability within SAR target classes (compared to natural images) may reduce the effectiveness of view-based contrastive learning, as augmented views may not provide sufficient discrimination signal.

### 4.9. Task Ablation Study and Pretext Task Analysis

To address fundamental questions about task contribution and framework complexity, we conducted comprehensive ablation studies systematically evaluating 15 task combinations across all 16 downstream classifiers at full data availability (*k* = 1.00) for our best-performing classifier (SVM). The nine pretext tasks were organized into four semantic groups: **T0_Original** (identity transformation), **T1–T5_Geometric** (rotations: 90^∘^, 180^∘^, 270^∘^; flips: horizontal, vertical), **T6–T7_SignalQuality** (denoise, blur), and **T8_MultiScale** (zoom). We evaluated individual groups, two-group combinations, and three-group combinations to identify optimal task selection strategies and quantify individual task contributions. The findings of the ablation studies are mentioned in Table 5.

The ablation study reveals that geometric transformation tasks (T1–T5) are unequivocally the most critical component, achieving 96.22% average accuracy across all downstream classifiers independently, substantially outperforming all other task groups. Individual task group performance demonstrates a clear hierarchy: T1–T5_Geometric (96.22%), T0_Original (89.46%), T8_MultiScale (88.71%), and T6–T7_SignalQuality (59.80%). The geometric tasks’ superiority stems from SAR domain-specific characteristics: radar target recognition inherently requires aspect-angle invariance, and rotation/flip transformations directly address arbitrary viewing angles in overhead imaging while preserving electromagnetic scattering properties and target structure.

The ablation study identifies tasks contributing minimally: T6–T7_SignalQuality alone achieves only 59.80% accuracy, indicating poor standalone discrimination. However, when combined with geometric tasks, signal quality tasks contribute positively (T1–T5 + T6–T7 achieves the best performance), suggesting complementary robustness rather than primary discriminative features. Similarly, T8_MultiScale (88.71% alone) provides moderate independent contribution but enhances specific architectures when combined with geometric tasks.

**Figure 5:**
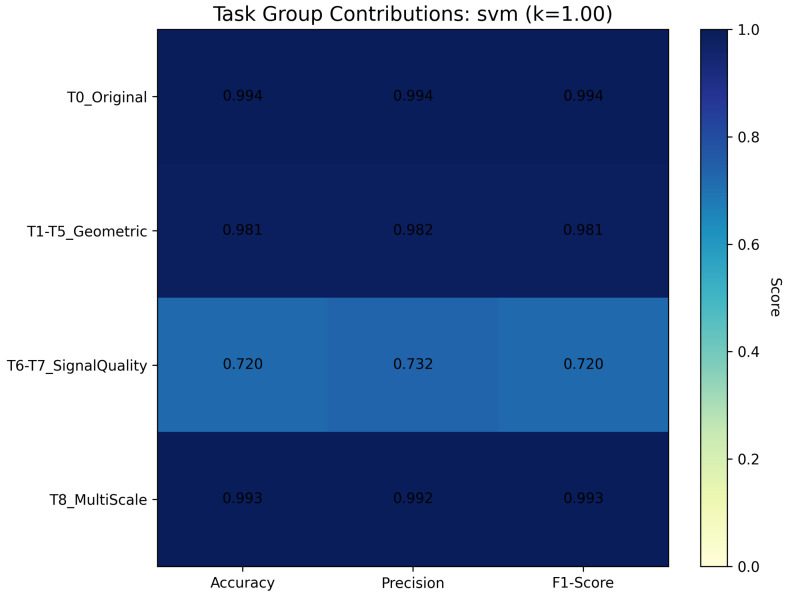
Task group contribution analysis at full data availability (*k* = 1.00) using SVM classifier. The heatmap shows performance metrics (accuracy, precision, F1-score) for individual task groups: T0_Original (identity transformation), T1–T5_Geometric (rotation and flip transformations), T6–T7_SignalQuality (denoise and blur), and T8_MultiScale (zoom).

The task contribution analysis for the task groups is depicted in Figure 5. As shown in the heatmap, T0_Original, T1–T5_Geometric, and T8_MultiScale maintain consistently high scores (0.981–0.994) across accuracy, precision, and F1-score, demonstrating robust discriminative capacity. In stark contrast, T6–T7_SignalQuality exhibits substantially degraded performance (0.720–0.732), confirming its limited standalone contribution while validating its role as a complementary robustness enhancer when combined with geometric transformations. The near-uniform performance of geometric tasks across all metrics (0.981–0.982) reinforces their position as the primary feature learning mechanism, while the marginal performance between T8_MultiScale (0.992–0.993) and geometric tasks suggests scale invariance provides incremental rather than fundamental improvements to the learned representations. Overall, achieving 99.63% accuracy using all the tasks shows that all tasks contribute in gaining performance.

### 4.10. Baseline Comparison and State-of-the-Art Performance

Our SSL framework demonstrates significant improvements over synthetic data approaches reported by Lewis et al. [3], achieving superior performance using exclusively measured data compared to their hybrid synthetic-measured approaches across all data availability scenarios. The elimination of synthetic data dependency while maintaining exceptional performance (99.63% accuracy for SVM) represents a fundamental advancement over previous methods that struggled with domain gap limitations.

The comparative analysis reveals our approach’s superiority across multiple dimensions: (1) elimination of synthetic data requirements removes domain gap constraints that limited previous approaches; (2) exclusive use of real SAR imagery ensures operational relevance and eliminates modeling assumptions inherent in synthetic generation; (3) superior performance metrics across diverse architectural families demonstrate framework generalizability; (4) robust performance under extreme data constraints (51.95% accuracy at *k* = 0.05) provides viable solutions for rapid deployment scenarios.

Our SSL framework substantially outperforms prior work across all evaluation metrics. While Lewis et al. [3] achieved 94.5% accuracy using CNN-based models on full measured datasets, our approach delivers superior performance across diverse architectural families. Table 6 presents comprehensive results for all classifiers achieving ≥94.50% accuracy at k = 1.00, demonstrating the framework’s effectiveness across traditional machine learning (SVM: 99.63%, Random Forest: 99.26%), efficient deep architectures (MobileNet variants: 95.73–98.70%, EfficientNet: 95.18–95.92%), standard deep networks (ResNet18: 97.40%), and generative models (GAN Classifier: 96.85%).

The results establish clear performance hierarchies: traditional ML approaches achieve exceptional accuracy when leveraging SSL-extracted features, with SVM demonstrating state-of-the-art performance (99.63%) that substantially exceeds previous benchmarks. Deep learning architectures maintain competitive performance, with ResNet18 leading at 97.40% accuracy, while efficient architectures provide compelling accuracy-efficiency trade-offs suitable for resource-constrained deployment scenarios.

**Figure 6:**
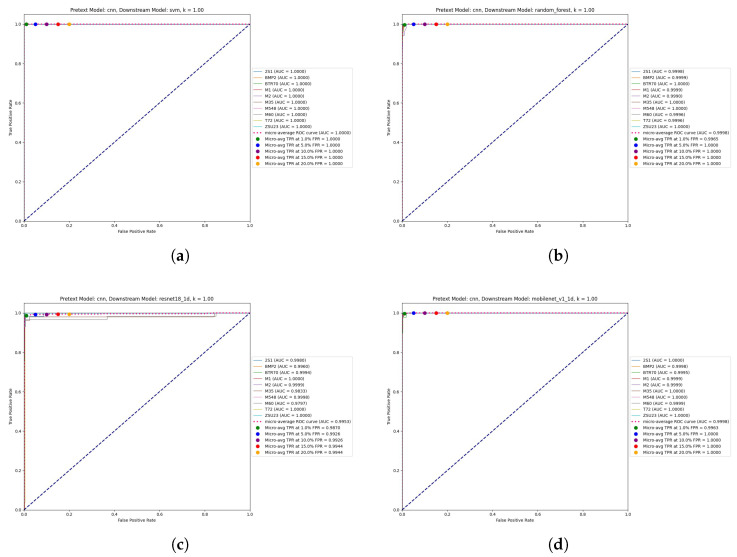
ROC curves for top-performing classifiers at *k* = 1.00, demonstrating exceptional discrimination capabilities across all SAR target classes. The near-perfect curves validate the superior quality of SSL-extracted features and confirm the framework’s effectiveness for operational SAR ATR applications. (**a**) SVM (99.63% accuracy); per-class AUC ≥ 0.9999. (**b**) Random Forest (99.26% accuracy); AUC 0.9996–1.0000. (**c**) ResNet18 (97.40% accuracy); AUC 0.9994–1.0000. (**d**) MobileNet (98.70% accuracy); efficient and near-perfect AUC.

Figure 6 presents ROC curves for the highest-performing classifiers at *k* = 1.00, demonstrating exceptional discrimination capabilities across all target classes. The visualization reveals near-perfect performance characteristics with AUC values approaching 1.0000 for SVM, Random Forest, ResNet18, and MobileNet variants, validating the superior quality of SSL-extracted features for SAR target recognition.

**Figure 7:**
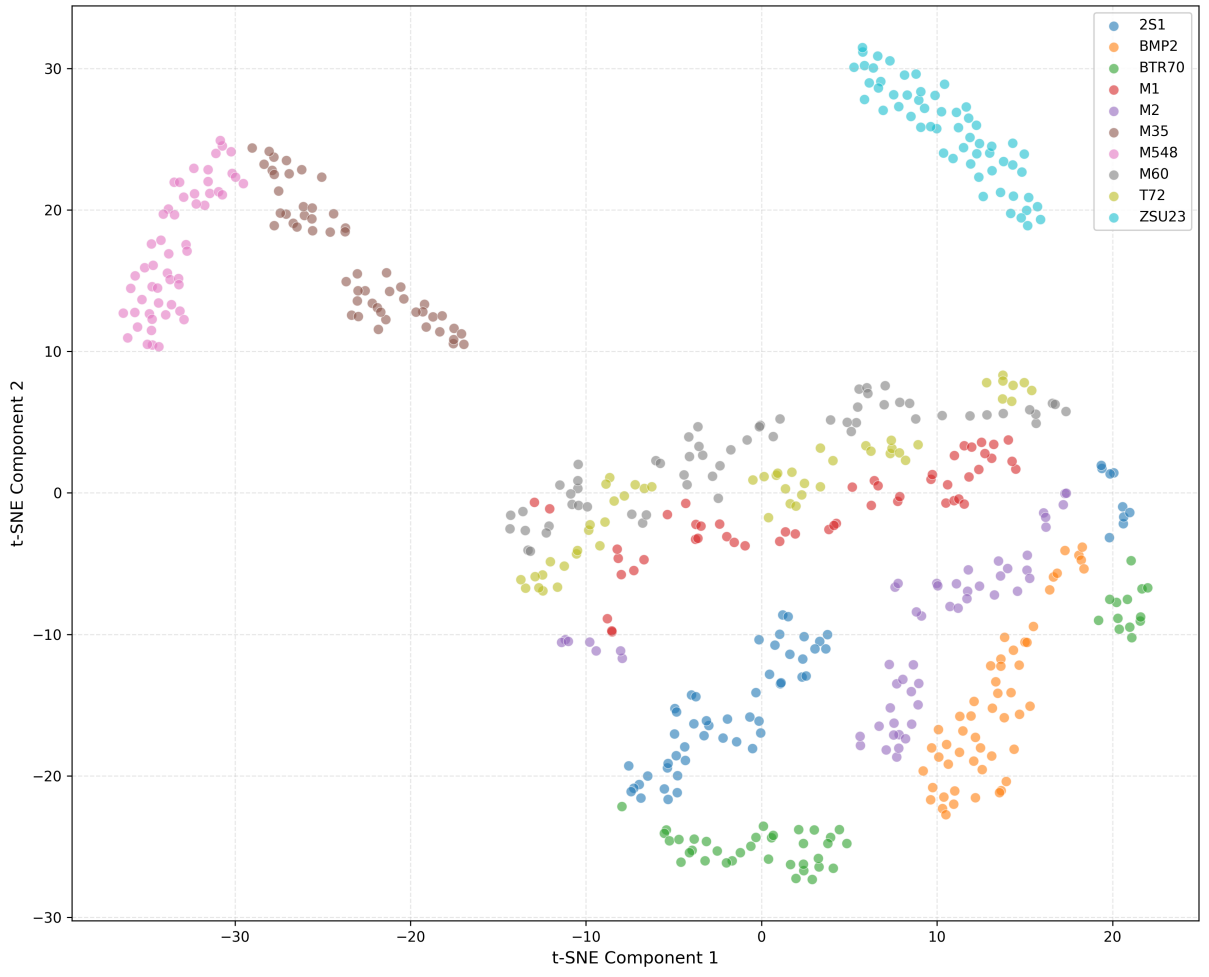
t-SNE visualization of 2048-length SSL features for the test set.

To visualize the quality of learned representations, we employ t-SNE (t-distributed Stochastic Neighbor Embedding) dimensionality reduction to project the 2048-length SSL-extracted features into 2D space. Figure 7 presents the t-SNE visualization for the test set, revealing distinct class clusters with minimal overlap. The clear separation between target classes demonstrates that our multi-task SSL framework learns semantically meaningful features that naturally group similar targets while maintaining discriminative boundaries between classes. SVM performs very well because the 2048-length SSL embeddings are close to linearly separable; a linear SVM maximizes inter-class margins in high dimensions with strong implicit regularization, which is advantageous in low-sample measured SAR. In contrast, tree ensembles and deep heads exhibit higher variance and tend to overfit speckle/idiosyncratic artifacts on limited data, while SVM leverages the global, geometry-aligned cues captured by the pretext encoder.

Comparison with SAR-Specific SSL Methods: Pei et al. [14] achieved 90.71% (using 10% of MSTAR data) and 99.34% (using 30% of MSTAR data) using SimCLR-style contrastive learning with ResNet50. Li et al. [15] introduced SAR-JEPA with predictive gradient-based embeddings, demonstrating robust low-data performance on MSTAR. SARATR-X [16] employs Vision Transformer pretraining on 180K samples from 14 datasets, while SAFE [18] uses masked Siamese ViT with self-distillation. These foundation models require millions of parameters, extensive multi-dataset curation, and substantial computational resources. Our lightweight CNN (97 K parameters, 2–3 min single-GPU training) achieves 99.63% accuracy with only 806 samples using SAMPLE dataset enhanced from MSTAR, demonstrating that physics-informed task design provides exceptional performance with dramatically lower cost and complexity compared to large-scale foundation approaches.

### 4.11. Detailed Results

Comprehensive experimental results are provided in the appendix tables. Appendix A Table A1 (Model Performance Metrics and Timing Results for *k*-value Experiment) presents detailed performance metrics and timing results for all 16 downstream classifiers across 20 different *k*-values (0.05–1.00), including accuracy, precision, recall, F1-score, ROC AUC, TPR at various FPR thresholds, training time, and inference time. Appendix A Table A2 (Class-wise ROC AUC Results for *k*-value Experiment) provides class-wise ROC AUC results for the *k*-value experiments, showing per-class discrimination performance for all 10 target classes. Appendix A Table A3 (Model Performance Metrics and Timing Results for Cross Validation Experiment) contains cross-validation performance metrics as well as the timing results (average of the fold metrics). Appendix A Table A4 (Class-wise ROC AUC Results for Cross Validation Experiment) presents class-wise ROC AUC results from the cross-validation experiments (average of the fold metrics). Appendix A Table A5 (SimCLR Performance Metrics) provides complete SimCLR baseline performance metrics across all *k*-values for comparison with our multi-task SSL approach.

## 5. Conclusions and Future Work

This work presents a comprehensive self-supervised learning framework for SAR automatic target recognition that fundamentally advances the state-of-the-art through systematic multi-task pretext training and extensive architectural evaluation. Our approach successfully eliminates dependency on synthetic data while achieving exceptional performance across diverse computational paradigms, providing critical insights for operational SAR ATR deployment through rigorous experimental validation on the SAMPLE dataset with 16 downstream classifiers spanning traditional machine learning, deep neural networks, efficient architectures, and generative models.

### 5.1. Key Findings and Contributions

The experimental results reveal fundamental insights that challenge conventional assumptions about deep learning superiority in SAR applications. Traditional machine learning approaches demonstrate exceptional effectiveness when combined with SSL-extracted features, with SVM achieving remarkable 99.63% accuracy, 99.63% precision, and 99.66% recall alongside near-perfect per-class ROC AUC values (1.0000 for six target classes, ≥0.9999 for four classes). Random Forest maintains the second-best performance with 99.26% accuracy and consistent per-class discrimination between 0.9960–1.0000, while XGBoost achieves competitive 93.88% accuracy with per-class ROC AUC above 0.9930. Among deep learning architectures, ResNet18 emerges as the top performer with 97.40% accuracy and exceptional per-class ROC AUC values between 0.9974–1.0000, demonstrating that convolutional approaches remain highly effective for SSL-based SAR feature classification.

The *k*-value experimental design provides insights into framework robustness under varying data constraints, systematically evaluating performance from extreme scarcity (*k* = 0.05, 43 samples) to full availability (*k* = 1.0, 806 samples). Traditional ML methods exhibit remarkable resilience at extreme data scarcity, with SVM achieving 51.95% accuracy and Random Forest reaching 46.01% accuracy at *k* = 0.05, far exceeding random chance performance (10%) and maintaining operationally relevant capabilities for preliminary target screening. Deep learning architectures show higher sensitivity to data availability but demonstrate rapid improvement as data increases, with ResNet18 advancing from 24.86% accuracy at *k* = 0.05 to 97.40% at *k* = 1.0, indicating architectural depth’s relationship to data requirements.

Computational efficiency analysis reveals highly favorable operational characteristics essential for real-world deployment. SSL feature extraction dominates processing time at 15.35 ms per image consistently across all classifiers, while inference times range from 0.01 ms (Gradient Boosting) to 0.37 ms (U-Net), enabling total processing times of 15.36–15.72 ms/image and supporting real-time operation at 58–65 Hz frame rates. Operational performance metrics demonstrate exceptional discrimination capability with SVM achieving 99.89% TPR at 1% FPR and perfect 100% TPR at 5% FPR, providing flexible deployment options across diverse mission requirements from high-precision reconnaissance to rapid threat assessment scenarios.

### 5.2. Theoretical and Practical Implications

The superior performance of traditional ML methods on SSL-extracted features suggests that the learned representations exhibit favorable linear separability characteristics, indicating that complex non-linear transformations may be unnecessary for effective SAR target discrimination when appropriate feature representations are available. This finding has significant practical implications for operational deployment, where traditional ML approaches offer computational efficiency advantages, interpretability benefits, and reduced training complexity compared to deep neural networks while maintaining state-of-the-art accuracy.

The framework’s elimination of synthetic data dependency addresses a critical limitation in SAR ATR research, where domain gap challenges between synthetic and measured imagery have consistently hindered practical deployment. By achieving exceptional performance using exclusively measured SAR imagery, our approach provides a viable path toward operational systems that can be deployed without extensive synthetic data generation infrastructure or domain adaptation techniques. The framework’s elimination of synthetic data dependency addresses a critical limitation in SAR ATR research, where domain gap challenges between synthetic and measured imagery have consistently hindered practical deployment. By achieving exceptional performance using exclusively measured SAR imagery, our approach provides a viable path toward operational systems that can be deployed without extensive synthetic data generation infrastructure or domain adaptation techniques.

Cross-validation experiments using a conservative 5-fold protocol with 920 real images validate framework robustness, with SVM achieving 99.13% mean accuracy and Random Forest maintaining 96.96% accuracy. The narrow confidence intervals demonstrate statistical significance and confirm that performance differences between methods reflect genuine capabilities rather than dataset-specific artifacts, providing confidence for deployment across varied operational scenarios.

### 5.3. Limitations and Methodological Considerations

Several limitations demand acknowledgment for comprehensive evaluation. Our experimental validation is constrained to the SAMPLE dataset (14–$17^{\circ }$ elevation angles, 10–$80^{\circ }$ azimuth range) with 1345 images, representing a specific operational scenario that is yet to be tested in diverse SAR sensor configurations, environmental conditions, or significantly larger target inventories encountered in comprehensive military applications. The framework’s performance across different radar frequencies, polarizations, and operational environments requires systematic investigation to establish generalizability bounds.

The *k*-value experimental design necessarily focuses on data availability scenarios where k≥0.10 for GAN-based classifiers due to minimum sample requirements for stable adversarial training. While this methodological choice ensures reliable results and prevents training artifacts, it limits insights into extremely low-data scenarios (k<0.10) for generative approaches. Additionally, while our computational analysis indicates promising timing characteristics, real-time deployment considerations, including hardware optimization, system integration overhead, and edge computing constraints require empirical validation across diverse operational platforms.

### 5.4. Future Research Directions

Several promising research directions emerge from this foundation that could significantly extend framework capabilities and operational applicability. Multi-modal integration incorporating multi-polarization, multi-frequency, and multi-temporal SAR data represents a natural extension that could enhance recognition capabilities while maintaining the synthetic data independence that characterizes our approach. SSL-based domain adaptation techniques could address operational condition variations and sensor differences without requiring extensive paired datasets.

Developing incremental learning frameworks that adapt to new target classes without full retraining would improve operational flexibility in dynamic threat environments where new vehicle types or configurations emerge continuously. Advanced pretext tasks design incorporating radar phenomenology principles, such as polarimetric signatures, coherence patterns, or aspect-dependent scattering behaviors, could further improve feature representation quality while maintaining computational efficiency.

Federated learning approaches could enable collaborative model development across multiple operational sites while preserving data privacy and security requirements critical for defense applications. Extension to sequential SAR data and video SAR analysis could leverage temporal information for enhanced recognition performance, particularly for moving targets or complex scenarios requiring temporal context.

Investigation of foundation model architectures specifically designed for radar data could establish large-scale pretraining frameworks that leverage diverse SAR datasets while maintaining the measured data focus that characterizes our current approach. Integration with modern transformer architectures and attention mechanisms could enable more sophisticated feature learning while preserving computational efficiency requirements for operational deployment.

As SAR technology evolves with advanced sensor capabilities, the principles established in this work provide a framework for developing adaptive, efficient automatic target recognition systems. The elimination of synthetic data dependency, combined with exceptional performance characteristics and computational efficiency, positions this approach as a foundation for next-generation SAR ATR applications, contributing to advances in remote sensing, computer vision, and artificial intelligence that extend beyond defense applications to scientific remote sensing, environmental monitoring, and civilian surveillance systems.

## Figures and Tables

**Table 1:** SAMPLE Dataset Distribution of Measured Images by Class and Elevation-Based Train/Test Split.

Class	Type	Wheeled?	Training Images	Test Images	Total
2S1	Tank	Tracked	116	58	174
BMP2	Tank	Tracked	55	52	107
BTR70	Tank	Wheeled	43	49	92
M1	Tank	Tracked	78	51	129
M2	Tank	Tracked	75	53	128
M35	Truck	Wheeled	76	53	129
M548	Truck	Tracked	75	53	128
M60	Tank	Tracked	116	60	176
T72	Tank	Tracked	56	52	108
ZSU23	Tank	Tracked	116	58	174
Total	–	–	806	539	1345

**Table 2:** CNN Architecture for Pretext Learning.

Layer	Output Shape	Parameters	Activation/Action
Input	(64, 64, 1)	–	Grayscale SAR image
Conv2D-1	(64, 64, 16)	3×3×1×16	ReLU
MaxPool-1	(32, 32, 16)	2×2	Spatial reduction
Conv2D-2	(32, 32, 32)	3×3×16×32	ReLU
MaxPool-2	(16, 16, 32)	2×2	Spatial reduction
Conv2D-3	(16, 16, 64)	3×3×32×64	ReLU
MaxPool-3	(8, 8, 64)	2×2	Spatial reduction
Conv2D-4	(8, 8, 128)	3×3×64×128	ReLU
MaxPool-4	(4, 4, 128)	2×2	Spatial reduction
Dense-1	(1000)	2048×1000	ReLU
Dense-2	(500)	1000×500	ReLU
Dense-3	(250)	500×250	ReLU
Output	(9)	250×9	Softmax

**Table 3:** Downstream Classifier Hyperparameters.

Model/Group	Key Hyperparameters
All Models	Feature scaling: StandardScaler (z-score); Feature dimension: 2048
Traditional Machine Learning Models	
SVM	Kernel: Linear; Probability output: Enabled; Random state: 42
Random Forest	Estimators: 100; Random state: 42; Parallel jobs: All cores
Gradient Boosting	Estimators: 100; Learning rate: 0.1; Random state: 42
XGBoost	Estimators: 100; Tree method: gpu_hist; Objective: multi:softprob; Random state: 42
Deep Learning Models (All: LR = 0.001, Epochs = 30, Batch = 8, Adam, Val = 0.15, Patience = 5)	
ResNet18	Input features: 2048
CNN	4 convolution layers
U-Net	Encoder depth: 4 levels
MobileNet v1	Width multipliers: 1.0, 0.75, 0.5, 0.25
EfficientNet	Variants: B0, B1, B2, B3
GAN Classifier	Generator LR: 0.0005; Discriminator LR: 0.001; Adversarial weight: 0.5

**Table 4:** Number of Training Samples Selected per Class for Different *k* Values.

* k *	Class										Total
	2S1	BMP2	BTR70	M1	M2	M35	M548	M60	T72	ZSU23	
	0	1	2	3	4	5	6	7	8	9	
0.05	6	3	3	4	4	4	4	6	3	6	43
0.10	12	6	5	8	8	8	8	12	6	12	85
0.15	18	9	7	12	12	12	12	18	9	18	127
0.20	24	11	9	16	15	16	15	24	12	24	166
0.25	29	14	11	20	19	19	19	29	14	29	203
0.30	35	17	13	24	23	23	23	35	17	35	245
0.35	41	20	16	28	27	27	27	41	20	41	288
0.40	47	22	18	32	30	31	30	47	23	47	327
0.45	53	25	20	36	34	35	34	53	26	53	369
0.50	58	28	22	39	38	38	38	58	28	58	405
0.55	64	31	24	43	42	42	42	64	31	64	447
0.60	70	34	26	47	46	46	46	70	34	70	489
0.65	76	36	28	51	49	50	49	76	37	76	528
0.70	82	39	31	55	53	54	53	82	40	82	571
0.75	88	42	33	59	57	58	57	88	43	88	613
0.80	93	44	35	63	60	61	60	93	45	93	647
0.85	99	47	37	67	64	65	64	99	48	99	689
0.90	105	50	39	71	68	69	68	105	51	105	731
0.95	111	53	41	75	72	73	72	111	54	111	773
1.00	116	55	43	78	75	76	75	116	56	116	806
Test	58	52	49	51	53	53	53	60	52	58	539

This table shows the number of training samples selected for each class at different *k*-values, where k represents the fraction of available training data used. The last row shows the test data distribution across all classes.

**Table 5:** Task Ablation Study Findings.

Rank	Task Combination	# Tasks	Avg Accuracy (%)
1	T1–T5_Geometric + T6–T7_SignalQuality	7	96.34
2	T1–T5_Geometric	5	96.22
3	T0_Original + T1–T5_Geometric	6	96.22
4	T1–T5_Geometric + T8_MultiScale	6	96.01
5	T1–T5 + T6–T7 + T8	8	95.03
6	T0 + T1–T5 + T8	7	94.58
7	All_Tasks (Full Framework)	9	94.18
8	T0 + T1–T5 + T6–T7	8	92.56
9	T6–T7 + T8	3	91.91
10	T0_Original	1	89.46
11	T8_MultiScale	1	88.71
12	T0 + T8	2	87.78
13	T0 + T6–T7	3	72.99
14	T0 + T6–T7 + T8	4	60.32
15	T6–T7_SignalQuality	2	59.80

**Table 6:** Accuracy (%) for Top Performing Classifiers (Those with ≥ 94.50% at *k* = 1.00).

*k*-Value	EfficientNet B1	EfficientNet B3	GAN	MobileNet v1	MobileNet v1 0.5	MobileNet v1 0.75	Random Forest	ResNet18	SVM	XGBoost
0.05	20.59	15.96	N/A	33.77	23.38	31.54	46.01	24.86	51.95	36.73
0.10	27.64	26.72	35.99	34.32	37.66	29.31	52.32	47.68	51.39	32.10
0.15	39.52	29.87	37.48	42.12	38.03	38.40	54.55	40.45	53.80	44.53
0.20	38.40	42.86	36.18	47.50	43.60	43.41	53.62	41.19	59.55	50.83
0.25	46.20	46.57	50.09	52.88	53.80	51.76	61.22	55.84	64.56	50.46
0.30	56.22	46.38	51.95	59.74	65.49	44.34	60.48	51.21	64.19	52.13
0.35	51.02	48.79	56.03	56.59	52.69	53.62	61.41	53.06	63.64	59.55
0.40	60.11	62.71	61.04	60.85	61.97	64.94	70.69	64.75	65.86	63.82
0.45	62.71	57.70	65.86	68.46	60.11	66.79	70.87	56.59	73.10	57.33
0.50	66.60	60.48	68.09	63.45	66.42	66.23	71.61	64.38	76.62	69.39
0.55	58.81	64.75	69.39	70.13	69.57	68.83	78.48	67.53	77.18	73.65
0.60	70.32	66.05	65.49	75.70	73.28	73.84	80.71	68.46	81.63	72.73
0.65	72.91	70.69	72.54	80.33	78.11	80.71	84.42	73.10	82.56	75.88
0.70	76.62	72.73	75.51	75.32	77.92	76.25	83.12	73.47	82.93	73.28
0.75	82.93	74.77	79.59	81.63	82.93	83.30	87.94	85.53	88.68	87.94
0.80	78.11	80.33	79.41	80.71	82.19	82.93	88.13	80.71	88.13	87.57
0.85	84.04	84.23	86.64	90.35	84.97	89.80	90.35	90.17	93.32	88.31
0.90	92.95	91.28	92.02	92.76	90.17	91.84	93.88	92.76	**95.36**	89.98
0.95	90.91	94.06	**95.55**	**96.10**	**96.29**	93.14	**97.22**	93.69	**98.89**	**96.10**
1.00	**95.18**	**95.92**	**96.85**	**98.70**	**95.73**	**97.77**	**99.26**	**97.40**	**99.63**	93.88

**Bold values** denote classifier accuracies meeting or exceeding the 94.50% threshold used to define top-performing models.

## Data Availability

The SAMPLE dataset used in this research is publicly available at https://github.com/benjaminlewis-afrl/SAMPLE_dataset_public (accessed on 8 December 2025). The implementation code and experimental configurations are available at https://github.com/MdAlSiam/ssl-sar-atr-2-v2/ (accessed on 8 December 2025).

## Abbreviations

The following abbreviations are used in this manuscript:

SAR	Synthetic Aperture Radar
ATR	Automatic Target Recognition
SSL	Self-Supervised Learning
CNN	Convolutional Neural Network
SVM	Support Vector Machine
RF	Random Forest
XGBoost	Extreme Gradient Boosting
GB	Gradient Boosting
ROC	Receiver Operating Characteristic
AUC	Area Under the Curve
TP	True Positive
TN	True Negative
FP	False Positive
FN	False Negative
TPR	True Positive Rate
FPR	False Positive Rate
MSTAR	Moving and Stationary Target Acquisition and Recognition
SAMPLE	Synthetic and Measured Paired and Labeled Experiment
GAN	Generative Adversarial Network
ViT	Vision Transformer

## Appendix A

**Table A1:** Model Performance Metrics and Timing Results (*k*-value experiment).

Pretext Model	Downstream Model	*k*	Accuracy	Precision	Recall	F1 Score	ROC AUC	TPR@FPR = 0.01	TPR@FPR = 0.05	TPR@FPR = 0.10	TPR@FPR = 0.15	Train Time (s)	Test Feat. Ext. (ms/img)	Inference Time (ms/img)
CNN	CNN	0.05	0.2727	0.4642	0.2604	0.2452	0.6210	0.0209	0.1047	0.3173	0.3544	0.43	15.35	0.06
CNN	CNN	0.10	0.4174	0.5700	0.4111	0.3868	0.7218	0.1096	0.3766	0.4675	0.5399	0.78	15.34	0.05
CNN	CNN	0.15	0.3358	0.4292	0.3304	0.2828	0.6236	0.0300	0.2263	0.3933	0.4416	1.17	15.34	0.05
CNN	CNN	0.20	0.4545	0.5782	0.4559	0.4539	0.7083	0.0590	0.4212	0.5399	0.5677	1.58	15.45	0.05
CNN	CNN	0.25	0.5046	0.6618	0.4998	0.5037	0.7849	0.0327	0.4787	0.6234	0.6790	1.91	15.36	0.05
CNN	CNN	0.30	0.4712	0.6030	0.4659	0.4706	0.7166	0.0483	0.4341	0.5399	0.5770	2.31	15.38	0.05
CNN	CNN	0.35	0.4694	0.6795	0.4717	0.4760	0.6893	0.0336	0.4100	0.5547	0.5696	2.60	15.31	0.05
CNN	CNN	0.40	0.5547	0.5812	0.5474	0.5383	0.8266	0.1828	0.5510	0.6456	0.7069	3.03	15.36	0.05
CNN	CNN	0.45	0.5250	0.5631	0.5182	0.5125	0.7441	0.1030	0.5139	0.5974	0.6289	3.49	15.39	0.05
CNN	CNN	0.50	0.6345	0.7154	0.6254	0.6273	0.8424	0.2157	0.6642	0.7254	0.7588	3.68	15.34	0.06
CNN	CNN	0.55	0.5955	0.6854	0.5935	0.5967	0.7872	0.0597	0.6197	0.6809	0.7032	4.12	15.28	0.05
CNN	CNN	0.60	0.6790	0.7443	0.6707	0.6663	0.8813	0.3265	0.7291	0.7829	0.8089	4.60	15.29	0.05
CNN	CNN	0.65	0.6568	0.7309	0.6488	0.6509	0.8492	0.2282	0.7013	0.7551	0.7755	4.96	15.35	0.05
CNN	CNN	0.70	0.7403	0.7683	0.7372	0.7358	0.9042	0.3803	0.8071	0.8423	0.8571	5.30	15.36	0.05
CNN	CNN	0.75	0.7681	0.8067	0.7629	0.7599	0.9415	0.6456	0.8367	0.8776	0.8998	5.75	15.37	0.05
CNN	CNN	0.80	0.8089	0.8300	0.8071	0.8075	0.9613	0.6698	0.8850	0.9109	0.9258	6.01	15.32	0.05
CNN	CNN	0.85	0.8200	0.8295	0.8150	0.8153	0.9501	0.7143	0.9017	0.9295	0.9351	6.17	15.31	0.05
CNN	CNN	0.90	0.8497	0.8528	0.8473	0.8477	0.9532	0.7495	0.9109	0.9332	0.9406	6.64	15.40	0.05
CNN	CNN	0.95	0.9072	0.9086	0.9042	0.9046	0.9816	0.9035	0.9462	0.9647	0.9740	7.07	15.37	0.05
CNN	CNN	1.00	0.9425	0.9431	0.9419	0.9417	0.9869	0.9592	0.9814	0.9833	0.9852	7.37	15.35	0.06
CNN	EfficientNet B0	0.05	0.1948	0.3997	0.1961	0.1720	0.5199	0.0034	0.0501	0.2393	0.3043	1.43	15.35	0.29
CNN	EfficientNet B0	0.10	0.2319	0.4550	0.2327	0.2124	0.5852	0.0000	0.0983	0.2876	0.3395	2.52	15.34	0.26
CNN	EfficientNet B0	0.15	0.3581	0.6299	0.3564	0.3718	0.6643	0.0148	0.2690	0.4119	0.4675	3.85	15.34	0.27
CNN	EfficientNet B0	0.20	0.4045	0.5523	0.4036	0.4144	0.8084	0.1874	0.3729	0.4694	0.5584	5.05	15.45	0.27
CNN	EfficientNet B0	0.25	0.5139	0.6031	0.5121	0.5350	0.8790	0.2672	0.5176	0.6327	0.7161	6.19	15.36	0.27
CNN	EfficientNet B0	0.30	0.4898	0.5721	0.4904	0.4806	0.8282	0.2672	0.4712	0.6067	0.6735	7.60	15.38	0.27
CNN	EfficientNet B0	0.35	0.5417	0.6234	0.5403	0.5447	0.8547	0.3618	0.5436	0.7050	0.7737	8.60	15.31	0.27
CNN	EfficientNet B0	0.40	0.6345	0.6689	0.6277	0.6198	0.9017	0.3915	0.6605	0.7384	0.7941	9.81	15.36	0.28
CNN	EfficientNet B0	0.45	0.6048	0.6447	0.6004	0.6096	0.8749	0.4082	0.6289	0.6939	0.7458	11.19	15.39	0.28
CNN	EfficientNet B0	0.50	0.6568	0.7485	0.6518	0.6524	0.9280	0.4972	0.6865	0.7774	0.8275	12.15	15.34	0.26
CNN	EfficientNet B0	0.55	0.6215	0.7044	0.6158	0.6196	0.8979	0.4267	0.6531	0.7365	0.7699	13.65	15.28	0.27
CNN	EfficientNet B0	0.60	0.6753	0.7473	0.6704	0.6765	0.9436	0.4768	0.7291	0.8163	0.8609	15.11	15.29	0.27
CNN	EfficientNet B0	0.65	0.6902	0.7165	0.6861	0.6862	0.9365	0.5325	0.7644	0.8553	0.8831	15.87	15.35	0.28
CNN	EfficientNet B0	0.70	0.7124	0.7499	0.7105	0.7081	0.9252	0.4694	0.7681	0.8404	0.8646	17.00	15.36	0.27
CNN	EfficientNet B0	0.75	0.8237	0.8242	0.8207	0.8191	0.9769	0.7848	0.8794	0.9406	0.9629	18.56	15.37	0.28
CNN	EfficientNet B0	0.80	0.7811	0.7903	0.7804	0.7706	0.9759	0.6735	0.8757	0.9314	0.9629	19.57	15.32	0.27
CNN	EfficientNet B0	0.85	0.8423	0.8794	0.8425	0.8470	0.9804	0.7922	0.9314	0.9518	0.9647	20.62	15.31	0.27
CNN	EfficientNet B0	0.90	0.9202	0.9231	0.9181	0.9173	0.9963	0.9351	0.9833	0.9944	0.9944	22.20	15.40	0.26
CNN	EfficientNet B0	0.95	0.9462	0.9472	0.9460	0.9455	0.9985	0.9573	0.9963	1.0000	1.0000	23.24	15.37	0.26
CNN	EfficientNet B0	1.00	0.9295	0.9325	0.9310	0.9300	0.9952	0.9499	0.9907	0.9944	0.9944	24.33	15.35	0.27
CNN	EfficientNet B1	0.05	0.2059	0.3984	0.2081	0.1958	0.5440	0.0000	0.0464	0.2430	0.3191	1.39	15.35	0.27
CNN	EfficientNet B1	0.10	0.2764	0.3961	0.2718	0.2120	0.6031	0.0148	0.2059	0.3098	0.3599	2.55	15.34	0.26
CNN	EfficientNet B1	0.15	0.3952	0.4918	0.3987	0.3944	0.7346	0.1187	0.3451	0.4917	0.5974	3.91	15.34	0.27
CNN	EfficientNet B1	0.20	0.3840	0.5716	0.3833	0.3660	0.7041	0.2226	0.3673	0.4193	0.4824	5.08	15.45	0.28
CNN	EfficientNet B1	0.25	0.4620	0.5625	0.4604	0.4600	0.8229	0.2245	0.4490	0.5603	0.6197	6.19	15.36	0.27
CNN	EfficientNet B1	0.30	0.5622	0.5976	0.5610	0.5628	0.8818	0.3210	0.5566	0.6753	0.7532	7.54	15.38	0.27
CNN	EfficientNet B1	0.35	0.5102	0.6263	0.5024	0.5002	0.8333	0.3432	0.5065	0.6122	0.6772	8.64	15.31	0.28
CNN	EfficientNet B1	0.40	0.6011	0.6270	0.5963	0.5939	0.8928	0.4119	0.6345	0.7328	0.7774	9.81	15.36	0.27
CNN	EfficientNet B1	0.45	0.6271	0.6750	0.6206	0.6133	0.8983	0.4082	0.6512	0.7458	0.8126	11.14	15.39	0.27
CNN	EfficientNet B1	0.50	0.6660	0.7256	0.6568	0.6511	0.9229	0.5121	0.7124	0.8015	0.8423	12.03	15.34	0.27
CNN	EfficientNet B1	0.55	0.5881	0.6811	0.5817	0.5867	0.8486	0.3135	0.6048	0.6976	0.7310	13.64	15.28	0.27
CNN	EfficientNet B1	0.60	0.7032	0.7294	0.6971	0.6952	0.9587	0.5900	0.7755	0.8646	0.9128	15.13	15.29	0.27
CNN	EfficientNet B1	0.65	0.7291	0.7609	0.7284	0.7317	0.9422	0.6197	0.7941	0.8534	0.8850	15.99	15.35	0.27
CNN	EfficientNet B1	0.70	0.7662	0.7874	0.7627	0.7562	0.9529	0.6605	0.8367	0.8757	0.9184	17.13	15.36	0.28
CNN	EfficientNet B1	0.75	0.8293	0.8375	0.8279	0.8284	0.9826	0.7551	0.9109	0.9647	0.9722	18.59	15.37	0.27
CNN	EfficientNet B1	0.80	0.7811	0.8072	0.7791	0.7737	0.9768	0.6494	0.8664	0.9443	0.9740	19.44	15.32	0.27
CNN	EfficientNet B1	0.85	0.8404	0.8569	0.8385	0.8394	0.9809	0.7978	0.9184	0.9592	0.9740	20.48	15.31	0.27
CNN	EfficientNet B1	0.90	0.9295	0.9311	0.9286	0.9286	0.9966	0.9406	0.9870	0.9944	0.9963	22.28	15.40	0.27
CNN	EfficientNet B1	0.95	0.9091	0.9129	0.9084	0.9071	0.9967	0.9128	0.9852	0.9981	1.0000	23.30	15.37	0.27
CNN	EfficientNet B1	1.00	0.9518	0.9528	0.9528	0.9514	0.9930	0.9629	0.9870	0.9907	0.9907	24.08	15.35	0.27
CNN	EfficientNet B2	0.05	0.2152	0.3481	0.2188	0.1460	0.5491	0.0148	0.1391	0.2468	0.3321	1.40	15.35	0.27
CNN	EfficientNet B2	0.10	0.2597	0.4622	0.2565	0.2265	0.5779	0.0093	0.1503	0.2950	0.3284	2.56	15.34	0.26
CNN	EfficientNet B2	0.15	0.3544	0.6693	0.3554	0.3765	0.6633	0.0000	0.2560	0.4267	0.5009	3.92	15.34	0.27
CNN	EfficientNet B2	0.20	0.4026	0.5796	0.3941	0.3926	0.7173	0.1373	0.3525	0.4824	0.5325	4.98	15.45	0.27
CNN	EfficientNet B2	0.25	0.4564	0.5329	0.4498	0.4348	0.8238	0.2523	0.4471	0.5566	0.6215	6.13	15.36	0.27
CNN	EfficientNet B2	0.30	0.5306	0.6103	0.5273	0.5207	0.8117	0.2468	0.5232	0.6122	0.6790	7.54	15.38	0.27
CNN	EfficientNet B2	0.35	0.4750	0.6345	0.4688	0.4737	0.7557	0.1985	0.4620	0.5473	0.6234	8.77	15.31	0.27
CNN	EfficientNet B2	0.40	0.6030	0.6251	0.5995	0.5921	0.8753	0.4212	0.6382	0.7199	0.7699	9.78	15.36	0.27
CNN	EfficientNet B2	0.45	0.5325	0.6323	0.5242	0.5264	0.8487	0.3748	0.5362	0.6197	0.7013	11.17	15.39	0.27
CNN	EfficientNet B2	0.50	0.5900	0.5998	0.5926	0.5694	0.9138	0.3952	0.6308	0.7403	0.8182	12.03	15.34	0.27
CNN	EfficientNet B2	0.55	0.5955	0.6929	0.5914	0.5939	0.8536	0.4286	0.6011	0.6994	0.7551	13.54	15.28	0.27
CNN	EfficientNet B2	0.60	0.6475	0.7081	0.6452	0.6519	0.9296	0.5065	0.6865	0.8089	0.8534	15.00	15.29	0.27
CNN	EfficientNet B2	0.65	0.7143	0.7515	0.7075	0.7024	0.9275	0.5696	0.7495	0.8126	0.8590	16.13	15.35	0.27
CNN	EfficientNet B2	0.70	0.7384	0.7823	0.7332	0.7317	0.9287	0.5844	0.7922	0.8516	0.8776	17.37	15.36	0.27
CNN	EfficientNet B2	0.75	0.8442	0.8575	0.8411	0.8437	0.9856	0.7941	0.9184	0.9629	0.9870	18.41	15.37	0.27
CNN	EfficientNet B2	0.80	0.7792	0.8161	0.7779	0.7786	0.9679	0.6586	0.8497	0.8924	0.9388	19.38	15.32	0.27
CNN	EfficientNet B2	0.85	0.9091	0.9133	0.9081	0.9078	0.9956	0.9128	0.9759	0.9944	0.9981	20.48	15.31	0.27
CNN	EfficientNet B2	0.90	0.9128	0.9163	0.9121	0.9112	0.9960	0.9147	0.9777	0.9926	0.9981	22.01	15.40	0.27
CNN	EfficientNet B2	0.95	0.9462	0.9479	0.9457	0.9451	0.9968	0.9666	0.9907	0.9907	0.9963	23.16	15.37	0.27
CNN	EfficientNet B2	1.00	0.8942	0.8999	0.8914	0.8918	0.9914	0.8794	0.9759	0.9852	0.9907	24.33	15.35	0.27
CNN	EfficientNet B3	0.05	0.1596	0.4854	0.1623	0.1478	0.5148	0.0000	0.0204	0.1874	0.3080	1.39	15.35	0.26
CNN	EfficientNet B3	0.10	0.2672	0.4860	0.2678	0.2291	0.6116	0.0482	0.1800	0.2968	0.3711	2.56	15.34	0.27
CNN	EfficientNet B3	0.15	0.2987	0.5224	0.2891	0.2523	0.6077	0.0114	0.1929	0.3395	0.3952	3.97	15.34	0.27
CNN	EfficientNet B3	0.20	0.4286	0.5469	0.4230	0.4120	0.7409	0.1521	0.3840	0.4935	0.5622	4.96	15.45	0.27
CNN	EfficientNet B3	0.25	0.4657	0.5952	0.4614	0.4682	0.8078	0.1911	0.4453	0.5436	0.6122	6.16	15.36	0.27
CNN	EfficientNet B3	0.30	0.4638	0.6367	0.4628	0.4568	0.7918	0.2078	0.4416	0.5510	0.5993	7.53	15.38	0.27
CNN	EfficientNet B3	0.35	0.4879	0.5670	0.4834	0.4748	0.7825	0.2579	0.4694	0.5584	0.6234	8.68	15.31	0.27
CNN	EfficientNet B3	0.40	0.6271	0.6569	0.6213	0.6082	0.9042	0.3989	0.6568	0.7328	0.7774	9.85	15.36	0.27
CNN	EfficientNet B3	0.45	0.5770	0.6732	0.5712	0.5667	0.8643	0.4304	0.5881	0.6531	0.6994	11.17	15.39	0.27
CNN	EfficientNet B3	0.50	0.6048	0.6804	0.6016	0.6125	0.9050	0.3284	0.6289	0.7291	0.7885	11.98	15.34	0.28
CNN	EfficientNet B3	0.55	0.6475	0.6948	0.6463	0.6487	0.8745	0.3711	0.6920	0.7718	0.8163	13.46	15.28	0.27
CNN	EfficientNet B3	0.60	0.6605	0.7469	0.6551	0.6527	0.9174	0.5121	0.7050	0.7718	0.8145	14.82	15.29	0.27
CNN	EfficientNet B3	0.65	0.7069	0.7412	0.7023	0.6971	0.9260	0.5510	0.7570	0.8293	0.8646	16.10	15.35	0.27
CNN	EfficientNet B3	0.70	0.7273	0.7666	0.7241	0.7242	0.9436	0.5510	0.7792	0.8497	0.8887	17.28	15.36	0.27
CNN	EfficientNet B3	0.75	0.7477	0.7721	0.7426	0.7376	0.9625	0.6438	0.8312	0.8942	0.9202	18.55	15.37	0.27
CNN	EfficientNet B3	0.80	0.8033	0.8229	0.8013	0.7953	0.9728	0.7050	0.8757	0.9221	0.9443	19.47	15.32	0.27
CNN	EfficientNet B3	0.85	0.8423	0.8556	0.8383	0.8383	0.9821	0.8126	0.9258	0.9536	0.9685	20.70	15.31	0.27
CNN	EfficientNet B3	0.90	0.9128	0.9192	0.9115	0.9137	0.9932	0.9091	0.9610	0.9814	0.9870	22.10	15.40	0.27
CNN	EfficientNet B3	0.95	0.9406	0.9413	0.9393	0.9395	0.9988	0.9685	0.9981	1.0000	1.0000	23.03	15.37	0.27
CNN	EfficientNet B3	1.00	0.9592	0.9589	0.9599	0.9592	0.9958	0.9814	0.9944	0.9944	0.9944	24.38	15.35	0.28
CNN	GAN Classifier	0.10	0.3599	0.4363	0.3464	0.2948	0.7647	0.1521	0.3265	0.4508	0.5436	0.75	15.34	0.09
CNN	GAN Classifier	0.15	0.3748	0.6377	0.3710	0.4010	0.6660	0.0047	0.3006	0.4286	0.4917	1.14	15.34	0.08
CNN	GAN Classifier	0.20	0.3618	0.5218	0.3622	0.3515	0.7119	0.0315	0.2801	0.4323	0.5250	1.35	15.45	0.08
CNN	GAN Classifier	0.25	0.5009	0.6768	0.4966	0.5060	0.8207	0.2709	0.4824	0.5881	0.6939	1.45	15.36	0.08
CNN	GAN Classifier	0.30	0.5195	0.6872	0.5174	0.5297	0.7928	0.0241	0.4991	0.6178	0.6642	1.77	15.38	0.08
CNN	GAN Classifier	0.35	0.5603	0.7556	0.5546	0.5745	0.7896	0.2078	0.5622	0.6642	0.7050	2.39	15.31	0.08
CNN	GAN Classifier	0.40	0.6104	0.6443	0.6076	0.5875	0.8904	0.4119	0.6401	0.7403	0.7922	2.73	15.36	0.08
CNN	GAN Classifier	0.45	0.6586	0.7402	0.6578	0.6617	0.8841	0.4416	0.6957	0.7811	0.8145	3.32	15.39	0.08
CNN	GAN Classifier	0.50	0.6809	0.7557	0.6731	0.6639	0.9209	0.4972	0.7180	0.8293	0.8646	3.56	15.34	0.08
CNN	GAN Classifier	0.55	0.6939	0.7763	0.6903	0.6952	0.8716	0.2044	0.7440	0.7978	0.8163	4.24	15.28	0.08
CNN	GAN Classifier	0.60	0.6549	0.7318	0.6502	0.6487	0.9268	0.4805	0.6883	0.7774	0.8367	4.14	15.29	0.09
CNN	GAN Classifier	0.65	0.7254	0.7521	0.7230	0.7201	0.9208	0.5121	0.7866	0.8497	0.8701	4.86	15.35	0.08
CNN	GAN Classifier	0.70	0.7551	0.8066	0.7494	0.7488	0.9270	0.5269	0.8126	0.8590	0.8794	5.16	15.36	0.08
CNN	GAN Classifier	0.75	0.7959	0.8315	0.7935	0.7905	0.9751	0.7347	0.8664	0.9314	0.9573	5.53	15.37	0.08
CNN	GAN Classifier	0.80	0.7941	0.8258	0.7930	0.7941	0.9747	0.6642	0.8887	0.9406	0.9647	5.48	15.32	0.08
CNN	GAN Classifier	0.85	0.8664	0.8769	0.8650	0.8650	0.9780	0.7978	0.9314	0.9666	0.9796	6.38	15.31	0.09
CNN	GAN Classifier	0.90	0.9202	0.9250	0.9194	0.9189	0.9944	0.9332	0.9796	0.9907	0.9944	6.75	15.40	0.08
CNN	GAN Classifier	0.95	0.9555	0.9575	0.9552	0.9550	0.9984	0.9685	0.9944	0.9981	1.0000	7.36	15.37	0.08
CNN	GAN Classifier	1.00	0.9685	0.9688	0.9679	0.9679	0.9946	0.9907	0.9926	0.9944	0.9944	6.88	15.35	0.08
CNN	Gradient Boosting	0.05	0.2616	0.4856	0.2595	0.2562	0.6416	0.0816	0.1707	0.3098	0.3859	4.58	15.35	0.01
CNN	Gradient Boosting	0.10	0.3469	0.5991	0.3361	0.3354	0.7043	0.1113	0.2653	0.4174	0.4712	13.39	15.34	0.01
CNN	Gradient Boosting	0.15	0.4787	0.5916	0.4787	0.4812	0.8224	0.2950	0.4638	0.5793	0.6549	18.17	15.34	0.01
CNN	Gradient Boosting	0.20	0.4879	0.5870	0.4876	0.4946	0.8396	0.3098	0.4527	0.5788	0.6456	26.25	15.45	0.01
CNN	Gradient Boosting	0.25	0.4434	0.5764	0.4379	0.4467	0.8080	0.3043	0.4193	0.4991	0.5492	28.91	15.36	0.01
CNN	Gradient Boosting	0.30	0.4861	0.5733	0.4803	0.4775	0.8426	0.3228	0.4638	0.5640	0.6327	34.86	15.38	0.01
CNN	Gradient Boosting	0.35	0.5362	0.6589	0.5284	0.5312	0.8399	0.3098	0.5250	0.5881	0.6327	53.46	15.31	0.01
CNN	Gradient Boosting	0.40	0.5751	0.6073	0.5691	0.5714	0.8781	0.3859	0.5863	0.6698	0.7328	40.68	15.36	0.01
CNN	Gradient Boosting	0.45	0.5232	0.6409	0.5164	0.5052	0.8217	0.3395	0.4972	0.5844	0.6549	58.62	15.39	0.01
CNN	Gradient Boosting	0.50	0.6679	0.7282	0.6675	0.6744	0.9289	0.5436	0.7069	0.7681	0.8293	61.47	15.34	0.01
CNN	Gradient Boosting	0.55	0.6475	0.7080	0.6423	0.6453	0.9263	0.5306	0.6679	0.7737	0.8312	43.10	15.28	0.01
CNN	Gradient Boosting	0.60	0.6605	0.7655	0.6573	0.6581	0.9053	0.5176	0.6920	0.7477	0.7718	60.45	15.29	0.01
CNN	Gradient Boosting	0.65	0.7217	0.7385	0.7134	0.7026	0.9388	0.6085	0.7681	0.8089	0.8442	60.62	15.35	0.01
CNN	Gradient Boosting	0.70	0.7291	0.7503	0.7226	0.7236	0.9136	0.5622	0.7848	0.8312	0.8571	109.09	15.36	0.01
CNN	Gradient Boosting	0.75	0.8386	0.8541	0.8369	0.8380	0.9802	0.7811	0.9202	0.9518	0.9666	84.51	15.37	0.01
CNN	Gradient Boosting	0.80	0.7959	0.8161	0.7948	0.7966	0.9718	0.7310	0.8646	0.9128	0.9425	90.14	15.32	0.01
CNN	Gradient Boosting	0.85	0.8349	0.8484	0.8317	0.8333	0.9744	0.7941	0.8942	0.9109	0.9332	100.24	15.31	0.01
CNN	Gradient Boosting	0.90	0.8738	0.8853	0.8703	0.8731	0.9910	0.8442	0.9499	0.9759	0.9852	101.46	15.40	0.01
CNN	Gradient Boosting	0.95	0.9332	0.9362	0.9307	0.9315	0.9965	0.9443	0.9907	0.9926	0.9944	83.16	15.37	0.01
CNN	Gradient Boosting	1.00	0.9276	0.9302	0.9279	0.9275	0.9951	0.9443	0.9740	0.9796	0.9944	91.64	15.35	0.01
CNN	MobileNet v1	0.05	0.3377	0.5454	0.3298	0.3129	0.7071	0.1224	0.3080	0.3952	0.4805	0.51	15.35	0.11
CNN	MobileNet v1	0.10	0.3432	0.6423	0.3431	0.3287	0.7613	0.2134	0.3080	0.4026	0.4991	0.85	15.34	0.10
CNN	MobileNet v1	0.15	0.4212	0.6155	0.4162	0.3812	0.7888	0.2597	0.3952	0.4935	0.5380	1.29	15.34	0.10
CNN	MobileNet v1	0.20	0.4750	0.6881	0.4728	0.4695	0.8260	0.3265	0.4360	0.5547	0.6401	1.68	15.45	0.11
CNN	MobileNet v1	0.25	0.5288	0.6570	0.5244	0.5239	0.8676	0.3432	0.5362	0.6475	0.7180	2.11	15.36	0.10
CNN	MobileNet v1	0.30	0.5974	0.7276	0.5952	0.5858	0.8962	0.4620	0.6104	0.7161	0.7737	2.48	15.38	0.11
CNN	MobileNet v1	0.35	0.5659	0.7073	0.5627	0.5426	0.8619	0.3822	0.5659	0.6345	0.6753	2.92	15.31	0.10
CNN	MobileNet v1	0.40	0.6085	0.7362	0.6039	0.6038	0.9003	0.4583	0.6327	0.7161	0.7737	3.23	15.36	0.10
CNN	MobileNet v1	0.45	0.6846	0.7863	0.6779	0.6758	0.9428	0.6030	0.7384	0.8219	0.8776	3.77	15.39	0.10
CNN	MobileNet v1	0.50	0.6345	0.8017	0.6311	0.6450	0.9085	0.3636	0.6716	0.7866	0.8386	4.09	15.34	0.10
CNN	MobileNet v1	0.55	0.7013	0.7815	0.6962	0.6975	0.9546	0.5844	0.7607	0.8590	0.8980	4.48	15.28	0.10
CNN	MobileNet v1	0.60	0.7570	0.8275	0.7511	0.7499	0.9508	0.6623	0.8256	0.8794	0.8961	4.88	15.29	0.10
CNN	MobileNet v1	0.65	0.8033	0.8312	0.7988	0.7948	0.9732	0.7384	0.8627	0.9017	0.9221	5.31	15.35	0.10
CNN	MobileNet v1	0.70	0.7532	0.8070	0.7476	0.7472	0.9634	0.6568	0.8367	0.9054	0.9499	5.77	15.36	0.10
CNN	MobileNet v1	0.75	0.8163	0.8350	0.8121	0.8091	0.9836	0.7829	0.9035	0.9425	0.9647	6.20	15.37	0.10
CNN	MobileNet v1	0.80	0.8071	0.8427	0.8042	0.7973	0.9778	0.7328	0.8776	0.9443	0.9629	6.40	15.32	0.10
CNN	MobileNet v1	0.85	0.9035	0.9113	0.9019	0.9006	0.9948	0.9165	0.9722	0.9852	0.9889	6.98	15.31	0.10
CNN	MobileNet v1	0.90	0.9276	0.9333	0.9268	0.9261	0.9964	0.9443	0.9777	0.9889	0.9981	7.40	15.40	0.10
CNN	MobileNet v1	0.95	0.9610	0.9618	0.9598	0.9602	0.9990	0.9814	0.9944	0.9963	1.0000	7.84	15.37	0.10
CNN	MobileNet v1	1.00	0.9870	0.9873	0.9867	0.9868	0.9998	0.9963	1.0000	1.0000	1.0000	8.02	15.35	0.10
CNN	MobileNet v1 0.25	0.05	0.2430	0.3416	0.2350	0.1535	0.6048	0.0612	0.1725	0.2393	0.2876	0.46	15.35	0.11
CNN	MobileNet v1 0.25	0.10	0.2894	0.4839	0.2807	0.2569	0.6742	0.0891	0.2078	0.4304	0.4954	0.81	15.34	0.09
CNN	MobileNet v1 0.25	0.15	0.3896	0.5768	0.3828	0.3523	0.7884	0.2078	0.3655	0.4917	0.5603	1.24	15.34	0.09
CNN	MobileNet v1 0.25	0.20	0.4434	0.5506	0.4346	0.4201	0.8629	0.2690	0.4490	0.5844	0.6827	1.65	15.45	0.09
CNN	MobileNet v1 0.25	0.25	0.4750	0.6339	0.4699	0.4545	0.7989	0.3024	0.4545	0.5455	0.6252	2.02	15.36	0.09
CNN	MobileNet v1 0.25	0.30	0.5065	0.6568	0.4985	0.4949	0.8440	0.2597	0.4972	0.6141	0.6939	2.51	15.38	0.10
CNN	MobileNet v1 0.25	0.35	0.5195	0.7129	0.5173	0.5222	0.8373	0.2931	0.5102	0.6327	0.7050	2.77	15.31	0.09
CNN	MobileNet v1 0.25	0.40	0.6178	0.6587	0.6131	0.5954	0.9281	0.4378	0.6494	0.7551	0.8312	3.24	15.36	0.09
CNN	MobileNet v1 0.25	0.45	0.6030	0.6734	0.5998	0.5976	0.9158	0.4564	0.6438	0.7570	0.8293	3.71	15.39	0.10
CNN	MobileNet v1 0.25	0.50	0.5881	0.6363	0.5809	0.5667	0.9046	0.4453	0.6234	0.7477	0.8108	4.01	15.34	0.09
CNN	MobileNet v1 0.25	0.55	0.7625	0.7902	0.7546	0.7517	0.9655	0.6419	0.8200	0.8868	0.9276	4.28	15.28	0.09
CNN	MobileNet v1 0.25	0.60	0.6790	0.7531	0.6713	0.6703	0.9497	0.5751	0.7403	0.8219	0.8905	4.93	15.29	0.09
CNN	MobileNet v1 0.25	0.65	0.7199	0.7767	0.7160	0.7200	0.9498	0.5195	0.7941	0.8683	0.8998	5.17	15.35	0.09
CNN	MobileNet v1 0.25	0.70	0.7254	0.8008	0.7205	0.7264	0.9486	0.5677	0.8052	0.8720	0.9035	5.43	15.36	0.09
CNN	MobileNet v1 0.25	0.75	0.8200	0.8277	0.8167	0.8170	0.9827	0.7440	0.9165	0.9573	0.9722	6.00	15.37	0.09
CNN	MobileNet v1 0.25	0.80	0.7477	0.7862	0.7444	0.7428	0.9488	0.6104	0.8200	0.8813	0.9091	6.15	15.32	0.09
CNN	MobileNet v1 0.25	0.85	0.8553	0.8663	0.8523	0.8520	0.9885	0.8163	0.9388	0.9685	0.9870	6.80	15.31	0.09
CNN	MobileNet v1 0.25	0.90	0.8646	0.8687	0.8630	0.8618	0.9892	0.8312	0.9481	0.9703	0.9796	6.94	15.40	0.09
CNN	MobileNet v1 0.5	0.60	0.7328	0.8209	0.7282	0.7353	0.9479	0.5807	0.7811	0.8571	0.8905	4.87	15.29	0.10
CNN	MobileNet v1 0.5	0.65	0.7811	0.8168	0.7792	0.7767	0.9663	0.6475	0.8553	0.8998	0.9276	5.12	15.35	0.10
CNN	MobileNet v1 0.5	0.70	0.7792	0.8056	0.7769	0.7739	0.9657	0.7161	0.8404	0.8887	0.9221	5.41	15.36	0.10
CNN	MobileNet v1 0.5	0.75	0.8293	0.8664	0.8269	0.8281	0.9780	0.7699	0.8998	0.9443	0.9573	6.08	15.37	0.09
CNN	MobileNet v1 0.5	0.80	0.8219	0.8368	0.8223	0.8162	0.9825	0.7403	0.9072	0.9536	0.9685	6.20	15.32	0.10
CNN	MobileNet v1 0.5	0.85	0.8497	0.8702	0.8457	0.8448	0.9844	0.8312	0.9109	0.9462	0.9685	6.85	15.31	0.10
CNN	MobileNet v1 0.5	0.90	0.9017	0.9120	0.8991	0.9011	0.9959	0.9091	0.9852	0.9963	0.9981	7.10	15.40	0.09
CNN	MobileNet v1 0.5	0.95	0.9629	0.9633	0.9626	0.9624	0.9990	0.9833	0.9944	1.0000	1.0000	7.66	15.37	0.09
CNN	MobileNet v1 0.5	1.00	0.9573	0.9572	0.9577	0.9569	0.9992	0.9740	0.9981	1.0000	1.0000	8.01	15.35	0.09
CNN	MobileNet v1 0.75	0.05	0.3154	0.4264	0.3120	0.2958	0.6829	0.0724	0.2393	0.3803	0.4638	0.46	15.35	0.10
CNN	MobileNet v1 0.75	0.10	0.2931	0.5609	0.2896	0.2724	0.6815	0.1447	0.2468	0.3469	0.4471	0.79	15.34	0.09
CNN	MobileNet v1 0.75	0.15	0.3840	0.6555	0.3832	0.3712	0.7509	0.2208	0.3395	0.4508	0.5417	1.23	15.34	0.09
CNN	MobileNet v1 0.75	0.20	0.4341	0.6001	0.4259	0.4202	0.7821	0.2041	0.4212	0.5399	0.6030	1.58	15.45	0.09
CNN	MobileNet v1 0.75	0.25	0.5176	0.7160	0.5059	0.5056	0.8138	0.2412	0.5083	0.5900	0.6438	2.02	15.36	0.09
CNN	MobileNet v1 0.75	0.30	0.4434	0.6759	0.4306	0.4012	0.7875	0.1911	0.4286	0.5269	0.5770	2.35	15.38	0.09
CNN	MobileNet v1 0.75	0.35	0.5362	0.6925	0.5346	0.5343	0.8521	0.3395	0.5380	0.6308	0.6957	2.86	15.31	0.09
CNN	MobileNet v1 0.75	0.40	0.6494	0.7882	0.6413	0.6348	0.9248	0.4620	0.6865	0.7755	0.8275	3.05	15.36	0.09
CNN	MobileNet v1 0.75	0.45	0.6679	0.7150	0.6646	0.6489	0.9217	0.5232	0.7087	0.7848	0.8293	3.60	15.39	0.09
CNN	MobileNet v1 0.75	0.50	0.6623	0.7339	0.6593	0.6483	0.9310	0.5603	0.6957	0.7978	0.8442	3.99	15.34	0.09
CNN	MobileNet v1 0.75	0.55	0.6883	0.7742	0.6820	0.6740	0.9340	0.4991	0.7532	0.8182	0.8646	4.38	15.28	0.09
CNN	MobileNet v1 0.75	0.60	0.7384	0.8034	0.7367	0.7474	0.9614	0.6067	0.8126	0.8924	0.9258	4.66	15.29	0.09
CNN	MobileNet v1 0.75	0.65	0.8071	0.8401	0.8018	0.7994	0.9757	0.7310	0.8757	0.9147	0.9481	5.17	15.35	0.10
CNN	MobileNet v1 0.75	0.70	0.7625	0.8233	0.7564	0.7527	0.9467	0.6494	0.8237	0.8683	0.8942	5.32	15.36	0.09
CNN	MobileNet v1 0.75	0.75	0.8330	0.8670	0.8294	0.8306	0.9790	0.7551	0.8924	0.9425	0.9592	6.07	15.37	0.09
CNN	MobileNet v1 0.75	0.80	0.8293	0.8611	0.8262	0.8237	0.9811	0.7477	0.8961	0.9518	0.9647	6.22	15.32	0.09
CNN	MobileNet v1 0.75	0.85	0.8980	0.9134	0.8961	0.8943	0.9932	0.8961	0.9666	0.9814	0.9889	6.61	15.31	0.09
CNN	MobileNet v1 0.75	0.90	0.9184	0.9207	0.9174	0.9171	0.9952	0.9314	0.9833	0.9907	0.9963	6.92	15.40	0.09
CNN	MobileNet v1 0.75	0.95	0.9314	0.9368	0.9306	0.9313	0.9978	0.9425	0.9944	0.9981	0.9981	7.57	15.37	0.09
CNN	MobileNet v1 0.75	1.00	0.9777	0.9780	0.9771	0.9771	0.9997	0.9944	1.0000	1.0000	1.0000	7.48	15.35	0.09
CNN	Random Forest	0.05	0.4601	0.5840	0.4445	0.4029	0.8301	0.2892	0.4695	0.5489	0.6177	0.09	15.35	0.03
CNN	Random Forest	0.10	0.5232	0.7121	0.5136	0.4985	0.8829	0.3206	0.5343	0.6418	0.7141	0.09	15.34	0.04
CNN	Random Forest	0.15	0.5455	0.7296	0.5410	0.5241	0.8923	0.3938	0.5489	0.6628	0.7370	0.08	15.34	0.05
CNN	Random Forest	0.20	0.5362	0.7023	0.5331	0.5323	0.8712	0.4428	0.5312	0.6424	0.7066	0.08	15.45	0.04
CNN	Random Forest	0.25	0.6122	0.7195	0.6099	0.6020	0.9356	0.5013	0.6663	0.7604	0.8586	0.09	15.36	0.05
CNN	Random Forest	0.30	0.6048	0.7365	0.5992	0.6006	0.9359	0.5127	0.6291	0.7546	0.8374	0.08	15.38	0.04
CNN	Random Forest	0.35	0.6141	0.7544	0.6085	0.6076	0.9204	0.4659	0.6460	0.7566	0.8192	0.09	15.31	0.04
CNN	Random Forest	0.40	0.7069	0.7705	0.6994	0.6880	0.9463	0.6069	0.7449	0.8182	0.8633	0.10	15.36	0.05
CNN	Random Forest	0.45	0.7087	0.8153	0.7033	0.6962	0.9447	0.5864	0.7253	0.8095	0.8702	0.09	15.39	0.05
CNN	Random Forest	0.50	0.7161	0.7657	0.7083	0.7015	0.9659	0.6076	0.7891	0.8746	0.9353	0.09	15.34	0.05
CNN	Random Forest	0.55	0.7848	0.8303	0.7770	0.7744	0.9766	0.7067	0.8488	0.9307	0.9585	0.09	15.28	0.05
CNN	Random Forest	0.60	0.8071	0.8412	0.8006	0.7968	0.9747	0.7405	0.8646	0.9204	0.9506	0.09	15.29	0.05
CNN	Random Forest	0.65	0.8442	0.8656	0.8394	0.8402	0.9839	0.7676	0.8989	0.9509	0.9778	0.09	15.35	0.05
CNN	Random Forest	0.70	0.8312	0.8543	0.8259	0.8262	0.9844	0.7906	0.9063	0.9391	0.9751	0.10	15.36	0.04
CNN	Random Forest	0.75	0.8794	0.8900	0.8761	0.8747	0.9888	0.8255	0.9377	0.9742	0.9833	0.10	15.37	0.04
CNN	Random Forest	0.80	0.8813	0.8948	0.8782	0.8780	0.9881	0.8286	0.9335	0.9691	0.9809	0.11	15.32	0.05
CNN	Random Forest	0.85	0.9035	0.9077	0.9011	0.9003	0.9927	0.8949	0.9493	0.9825	0.9899	0.10	15.31	0.05
CNN	Random Forest	0.90	0.9388	0.9463	0.9373	0.9385	0.9960	0.9342	0.9870	0.9926	0.9944	0.11	15.40	0.04
CNN	Random Forest	0.95	0.9722	0.9731	0.9709	0.9716	0.9993	0.9837	0.9981	1.0000	1.0000	0.10	15.37	0.05
CNN	Random Forest	1.00	0.9926	0.9930	0.9922	0.9924	0.9998	0.9965	1.0000	1.0000	1.0000	0.10	15.35	0.05
CNN	ResNet18	0.05	0.2486	0.4883	0.2466	0.2021	0.5780	0.0445	0.1967	0.2764	0.3395	0.69	15.35	0.15
CNN	ResNet18	0.10	0.4768	0.7477	0.4747	0.4427	0.7279	0.2356	0.4601	0.5288	0.5584	1.13	15.34	0.13
CNN	ResNet18	0.15	0.4045	0.6644	0.4035	0.3709	0.7427	0.2375	0.3748	0.4360	0.5083	1.74	15.34	0.13
CNN	ResNet18	0.20	0.4119	0.6128	0.4131	0.4034	0.7502	0.0482	0.3729	0.5269	0.5770	2.22	15.45	0.13
CNN	ResNet18	0.25	0.5584	0.7627	0.5551	0.5530	0.8593	0.3024	0.5566	0.6883	0.7365	2.72	15.36	0.13
CNN	ResNet18	0.30	0.5121	0.7305	0.5097	0.5210	0.7715	0.1002	0.5028	0.6104	0.6605	3.28	15.38	0.13
CNN	ResNet18	0.35	0.5306	0.7097	0.5309	0.5375	0.7885	0.1725	0.5473	0.6197	0.6846	3.79	15.31	0.13
CNN	ResNet18	0.40	0.6475	0.7838	0.6462	0.6392	0.8838	0.4304	0.6846	0.7458	0.7737	4.27	15.36	0.13
CNN	ResNet18	0.45	0.5659	0.7095	0.5596	0.5653	0.8522	0.3711	0.5788	0.6920	0.7514	4.88	15.39	0.13
CNN	ResNet18	0.50	0.6438	0.7389	0.6382	0.6295	0.8663	0.4286	0.6939	0.7570	0.8126	5.21	15.34	0.13
CNN	ResNet18	0.55	0.6753	0.7442	0.6680	0.6708	0.8981	0.5269	0.7087	0.7662	0.8071	5.80	15.28	0.13
CNN	ResNet18	0.60	0.6846	0.8173	0.6804	0.6899	0.9267	0.5547	0.7440	0.8182	0.8627	6.49	15.29	0.13
CNN	ResNet18	0.65	0.7310	0.8101	0.7256	0.7321	0.9346	0.5733	0.7978	0.8534	0.8757	6.81	15.35	0.13
CNN	ResNet18	0.70	0.7347	0.8045	0.7299	0.7281	0.8778	0.6197	0.7904	0.8367	0.8497	7.34	15.36	0.13
CNN	ResNet18	0.75	0.8553	0.8610	0.8524	0.8486	0.9788	0.8275	0.9295	0.9481	0.9647	8.06	15.37	0.13
CNN	ResNet18	0.80	0.8071	0.8172	0.8045	0.8016	0.9737	0.7495	0.8924	0.9369	0.9536	8.36	15.32	0.13
CNN	ResNet18	0.85	0.9017	0.9096	0.8991	0.8993	0.9807	0.8998	0.9443	0.9647	0.9777	8.81	15.31	0.13
CNN	ResNet18	0.90	0.9276	0.9359	0.9261	0.9262	0.9946	0.9332	0.9814	0.9889	0.9944	9.51	15.40	0.13
CNN	ResNet18	0.95	0.9369	0.9425	0.9368	0.9372	0.9977	0.9481	0.9926	0.9981	0.9981	10.00	15.37	0.13
CNN	ResNet18	1.00	0.9740	0.9741	0.9751	0.9741	0.9953	0.9870	0.9926	0.9926	0.9944	10.42	15.35	0.13
CNN	SVM	0.05	0.5195	0.7169	0.5133	0.4838	0.8550	0.2375	0.4842	0.6122	0.6698	0.00	15.35	0.01
CNN	SVM	0.10	0.5139	0.6975	0.5146	0.5153	0.8899	0.2690	0.5770	0.7032	0.7495	0.01	15.34	0.02
CNN	SVM	0.15	0.5380	0.6920	0.5380	0.5258	0.9026	0.3562	0.6048	0.7032	0.7625	0.01	15.34	0.02
CNN	SVM	0.20	0.5955	0.6932	0.5939	0.5882	0.9186	0.3822	0.6642	0.7551	0.7978	0.02	15.45	0.03
CNN	SVM	0.25	0.6456	0.7652	0.6429	0.6466	0.9392	0.5158	0.6716	0.7885	0.8683	0.02	15.36	0.04
CNN	SVM	0.30	0.6419	0.7670	0.6375	0.6474	0.9278	0.4675	0.6883	0.7737	0.8516	0.04	15.38	0.04
CNN	SVM	0.35	0.6364	0.7935	0.6307	0.6417	0.9430	0.4917	0.7161	0.7978	0.8794	0.03	15.31	0.04
CNN	SVM	0.40	0.6586	0.7495	0.6533	0.6531	0.9465	0.5436	0.7161	0.8349	0.8924	0.05	15.36	0.05
CNN	SVM	0.45	0.7310	0.8283	0.7250	0.7260	0.9599	0.6605	0.7755	0.8442	0.9035	0.08	15.39	0.05
CNN	SVM	0.50	0.7662	0.8512	0.7603	0.7616	0.9671	0.6494	0.8404	0.8998	0.9332	0.06	15.34	0.05
CNN	SVM	0.55	0.7718	0.8297	0.7675	0.7668	0.9797	0.7013	0.8757	0.9499	0.9759	0.08	15.28	0.05
CNN	SVM	0.60	0.8163	0.8642	0.8136	0.8158	0.9828	0.7551	0.9017	0.9555	0.9759	0.13	15.29	0.06
CNN	SVM	0.65	0.8256	0.8661	0.8198	0.8201	0.9791	0.7718	0.8850	0.9388	0.9610	0.12	15.35	0.07
CNN	SVM	0.70	0.8293	0.8767	0.8241	0.8259	0.9815	0.7904	0.9109	0.9499	0.9703	0.12	15.36	0.07
CNN	SVM	0.75	0.8868	0.8992	0.8842	0.8830	0.9917	0.8738	0.9555	0.9722	0.9814	0.18	15.37	0.07
CNN	SVM	0.80	0.8813	0.9022	0.8787	0.8784	0.9915	0.8701	0.9481	0.9777	0.9926	0.16	15.32	0.09
CNN	SVM	0.85	0.9332	0.9408	0.9314	0.9314	0.9971	0.9425	0.9777	0.9944	1.0000	0.15	15.31	0.08
CNN	SVM	0.90	0.9536	0.9554	0.9528	0.9529	0.9992	0.9722	0.9981	1.0000	1.0000	0.23	15.40	0.08
CNN	SVM	0.95	0.9889	0.9888	0.9890	0.9889	1.0000	1.0000	1.0000	1.0000	1.0000	0.23	15.37	0.09
CNN	SVM	1.00	0.9963	0.9963	0.9966	0.9964	1.0000	1.0000	1.0000	1.0000	1.0000	0.21	15.35	0.08
CNN	U-Net	0.05	0.2783	0.6098	0.2689	0.2403	0.6347	0.0649	0.1577	0.3618	0.4304	1.64	15.35	0.39
CNN	U-Net	0.10	0.3117	0.6361	0.3035	0.2839	0.7050	0.1429	0.2727	0.3822	0.4508	3.05	15.34	0.37
CNN	U-Net	0.15	0.4267	0.6452	0.4258	0.4023	0.7622	0.1651	0.4137	0.5046	0.5603	4.64	15.34	0.37
CNN	U-Net	0.20	0.4768	0.5695	0.4754	0.4415	0.8321	0.2672	0.4991	0.6234	0.6865	6.03	15.45	0.37
CNN	U-Net	0.25	0.4323	0.4564	0.4231	0.4008	0.8347	0.2579	0.4212	0.5250	0.6549	7.35	15.36	0.37
CNN	U-Net	0.30	0.4323	0.5781	0.4250	0.3851	0.7902	0.2560	0.4137	0.5492	0.6252	8.90	15.38	0.37
CNN	U-Net	0.35	0.6085	0.7284	0.5994	0.5873	0.8860	0.3915	0.6531	0.7254	0.7978	10.41	15.31	0.37
CNN	U-Net	0.40	0.5659	0.7267	0.5583	0.5495	0.8585	0.3024	0.5733	0.6605	0.7180	11.75	15.36	0.37
CNN	U-Net	0.45	0.5436	0.6260	0.5357	0.5184	0.8795	0.4360	0.5714	0.6939	0.7811	13.33	15.39	0.37
CNN	U-Net	0.50	0.7032	0.7533	0.7012	0.6953	0.9449	0.5455	0.7477	0.8200	0.8757	14.46	15.34	0.37
CNN	U-Net	0.55	0.6531	0.7006	0.6515	0.6456	0.9311	0.5195	0.6939	0.8015	0.8776	16.11	15.28	0.37
CNN	U-Net	0.60	0.7495	0.7794	0.7457	0.7388	0.9716	0.6698	0.8479	0.9165	0.9406	17.65	15.29	0.37
CNN	U-Net	0.65	0.7588	0.7811	0.7534	0.7494	0.9682	0.6197	0.8553	0.9109	0.9406	19.08	15.35	0.37
CNN	U-Net	0.70	0.7421	0.7934	0.7370	0.7327	0.9421	0.6048	0.7959	0.8497	0.8757	20.48	15.36	0.37
CNN	U-Net	0.75	0.7996	0.8125	0.7961	0.7876	0.9674	0.7310	0.8701	0.8980	0.9221	22.05	15.37	0.37
CNN	U-Net	0.80	0.7941	0.8120	0.7905	0.7872	0.9789	0.6846	0.8813	0.9462	0.9666	23.27	15.32	0.37
CNN	U-Net	0.85	0.8664	0.8775	0.8637	0.8604	0.9879	0.8497	0.9406	0.9685	0.9814	24.65	15.31	0.37
CNN	U-Net	0.90	0.8479	0.8675	0.8436	0.8469	0.9816	0.7458	0.9239	0.9518	0.9703	26.21	15.40	0.37
CNN	U-Net	0.95	0.9184	0.9223	0.9163	0.9163	0.9970	0.9295	0.9889	0.9981	0.9981	27.69	15.37	0.37
CNN	U-Net	1.00	0.8404	0.8636	0.8383	0.8373	0.9874	0.7718	0.9425	0.9796	0.9907	28.98	15.35	0.37
CNN	XGBoost	0.05	0.3673	0.4855	0.3533	0.3295	0.6944	0.1558	0.3098	0.3581	0.4249	0.28	15.35	0.04
CNN	XGBoost	0.10	0.3210	0.5714	0.3084	0.3010	0.7239	0.0983	0.2690	0.3803	0.5232	0.32	15.34	0.02
CNN	XGBoost	0.15	0.4453	0.5313	0.4380	0.4100	0.7923	0.2393	0.4286	0.5269	0.5993	0.36	15.34	0.02
CNN	XGBoost	0.20	0.5083	0.6166	0.5071	0.4895	0.8546	0.3284	0.5213	0.6104	0.6735	0.41	15.45	0.02
CNN	XGBoost	0.25	0.5046	0.5653	0.4982	0.4915	0.8673	0.3711	0.5213	0.6456	0.7310	0.47	15.36	0.02
CNN	XGBoost	0.30	0.5213	0.6166	0.5143	0.5095	0.8836	0.3525	0.5380	0.6568	0.7495	0.52	15.38	0.02
CNN	XGBoost	0.35	0.5955	0.6787	0.5898	0.5792	0.8961	0.3933	0.6160	0.7291	0.7755	0.64	15.31	0.02
CNN	XGBoost	0.40	0.6382	0.6837	0.6326	0.6207	0.9300	0.5102	0.6809	0.7941	0.8609	0.60	15.36	0.02
CNN	XGBoost	0.45	0.5733	0.7111	0.5667	0.5698	0.8576	0.3358	0.6104	0.6883	0.7273	0.65	15.39	0.02
CNN	XGBoost	0.50	0.6939	0.7200	0.6907	0.6932	0.9636	0.5900	0.7755	0.8757	0.9351	0.73	15.34	0.02
CNN	XGBoost	0.55	0.7365	0.7844	0.7294	0.7297	0.9690	0.6419	0.8312	0.9184	0.9462	0.63	15.28	0.02
CNN	XGBoost	0.60	0.7273	0.8107	0.7222	0.7231	0.9590	0.6494	0.7904	0.8794	0.9184	0.74	15.29	0.02
CNN	XGBoost	0.65	0.7588	0.7712	0.7531	0.7513	0.9607	0.6512	0.8163	0.8776	0.9202	0.75	15.35	0.02
CNN	XGBoost	0.70	0.7328	0.7550	0.7263	0.7222	0.9606	0.6289	0.8108	0.8998	0.9221	0.96	15.36	0.02
CNN	XGBoost	0.75	0.8794	0.8916	0.8768	0.8780	0.9889	0.8534	0.9555	0.9796	0.9833	0.91	15.37	0.02
CNN	XGBoost	0.80	0.8757	0.8954	0.8738	0.8746	0.9853	0.8330	0.9314	0.9499	0.9666	0.95	15.32	0.02
CNN	XGBoost	0.85	0.8831	0.8905	0.8804	0.8813	0.9914	0.8720	0.9573	0.9759	0.9889	0.92	15.31	0.02
CNN	XGBoost	0.90	0.8998	0.9055	0.8980	0.8974	0.9943	0.8942	0.9703	0.9870	0.9926	0.99	15.40	0.02
CNN	XGBoost	0.95	0.9610	0.9631	0.9595	0.9600	0.9990	0.9703	1.0000	1.0000	1.0000	0.94	15.37	0.02
CNN	XGBoost	1.00	0.9388	0.9408	0.9387	0.9390	0.9979	0.9610	0.9889	0.9963	0.9981	0.97	15.35	0.02

**Table A2:** Class-wise ROC AUC Results.

Pretext Model	Downstream Model	*k*	ROC AUC Class 0	ROC AUC Class 1	ROC AUC Class 2	ROC AUC Class 3	ROC AUC Class 4	ROC AUC Class 5	ROC AUC Class 6	ROC AUC Class 7	ROC AUC Class 8	ROC AUC Class 9
CNN	CNN	0.05	0.5568	0.7209	0.5219	0.4380	0.6457	0.7334	0.7919	0.6927	0.4587	0.7500
CNN	CNN	0.10	0.7390	0.8567	0.5920	0.7426	0.8580	0.3955	0.8208	0.8817	0.7599	0.8620
CNN	CNN	0.15	0.7413	0.7178	0.5119	0.6032	0.5930	0.2652	0.7382	0.5903	0.6354	0.9373
CNN	CNN	0.20	0.7674	0.8638	0.6368	0.5936	0.5579	0.7122	0.9944	0.5998	0.6374	0.7939
CNN	CNN	0.25	0.7984	0.9121	0.6135	0.6763	0.5776	0.9331	0.9940	0.6906	0.7286	0.9559
CNN	CNN	0.30	0.8041	0.7265	0.5957	0.6430	0.5632	0.7720	0.7124	0.6803	0.7229	0.9490
CNN	CNN	0.35	0.7636	0.9306	0.6713	0.4892	0.5066	0.6423	0.8176	0.5022	0.7995	0.8855
CNN	CNN	0.40	0.8419	0.7903	0.6702	0.8461	0.7251	0.7800	0.8889	0.8962	0.7914	0.9722
CNN	CNN	0.45	0.7527	0.6353	0.6523	0.8581	0.7487	0.6378	0.7648	0.8750	0.8128	0.7460
CNN	CNN	0.50	0.9615	0.8719	0.7120	0.6624	0.7222	0.9026	0.9357	0.8968	0.8392	0.9835
CNN	CNN	0.55	0.8395	0.8092	0.7108	0.7896	0.6909	0.5993	0.9503	0.8256	0.8453	0.8172
CNN	CNN	0.60	0.9653	0.9339	0.8867	0.9202	0.8470	0.7131	0.6224	0.9415	0.9289	0.9944
CNN	CNN	0.65	0.9259	0.9036	0.8015	0.7302	0.7924	0.9077	0.6826	0.9665	0.9155	0.8765
CNN	CNN	0.70	0.8832	0.9748	0.8903	0.8994	0.8695	0.9111	0.7348	0.8954	0.9402	0.9991
CNN	CNN	0.75	0.9564	0.9245	0.9459	0.8746	0.8925	0.9730	0.9882	0.9491	0.9758	0.9634
CNN	CNN	0.80	0.9810	0.9282	0.9748	0.9134	0.9328	0.9959	0.9741	0.9673	0.9720	0.9769
CNN	CNN	0.85	0.9920	0.8566	0.9258	0.9127	0.9265	0.9776	0.9342	0.9637	0.9869	0.9921
CNN	CNN	0.90	0.9810	0.9669	0.9022	0.9524	0.8673	0.9637	0.9557	0.9828	0.9457	0.9935
CNN	CNN	0.95	0.9754	0.9620	0.9752	0.9903	0.9643	0.9800	1.0000	0.9950	0.9813	0.9929
CNN	CNN	1.00	0.9989	0.9985	0.9900	0.9808	0.9949	0.9677	0.9962	0.9490	0.9952	1.0000
CNN	EfficientNet B0	0.05	0.4932	0.6559	0.4841	0.4464	0.4314	0.5132	0.5381	0.4369	0.5265	0.6345
CNN	EfficientNet B0	0.10	0.7095	0.8147	0.1334	0.6790	0.6787	0.3839	0.4974	0.5951	0.6924	0.8371
CNN	EfficientNet B0	0.15	0.8558	0.7545	0.3730	0.6349	0.6438	0.5806	0.6745	0.5923	0.7538	0.8760
CNN	EfficientNet B0	0.20	0.7122	0.9366	0.9440	0.5616	0.8063	0.7635	0.7969	0.7337	0.8197	0.9639
CNN	EfficientNet B0	0.25	0.9355	0.9574	0.9725	0.7361	0.6659	0.7761	0.9619	0.8864	0.8216	0.9889
CNN	EfficientNet B0	0.30	0.8688	0.8132	0.8677	0.8318	0.6778	0.8534	0.7690	0.8232	0.8743	0.9783
CNN	EfficientNet B0	0.35	0.6965	0.9921	0.7626	0.8726	0.9347	0.8010	0.7752	0.8396	0.9247	0.9184
CNN	EfficientNet B0	0.40	0.9670	0.9212	0.9354	0.7887	0.6692	0.8088	0.9810	0.9709	0.9570	0.9996
CNN	EfficientNet B0	0.45	0.8457	0.7836	0.9511	0.8927	0.8980	0.8557	0.9029	0.8657	0.8644	0.9108
CNN	EfficientNet B0	0.50	0.9810	0.9797	0.9618	0.9167	0.8664	0.9977	0.9838	0.9078	0.9200	0.9670
CNN	EfficientNet B0	0.55	0.9390	0.9362	0.8504	0.8789	0.8826	0.9024	0.9378	0.9907	0.8854	0.8636
CNN	EfficientNet B0	0.60	0.9606	0.9776	0.9621	0.8785	0.9043	0.8644	0.9987	0.9730	0.9138	0.9922
CNN	EfficientNet B0	0.65	0.9554	0.9721	0.9655	0.8953	0.9591	0.9764	0.8396	0.9665	0.8835	0.9846
CNN	EfficientNet B0	0.70	0.9247	0.9944	0.9741	0.8758	0.9128	0.9239	0.8614	0.9573	0.9254	0.9942
CNN	EfficientNet B0	0.75	0.9720	0.9675	0.9928	0.9521	0.9429	0.9686	0.9995	0.9712	0.9852	0.9998
CNN	EfficientNet B0	0.80	0.9863	0.9879	0.9886	0.9495	0.9125	0.9659	0.9971	0.9784	0.9874	1.0000
CNN	EfficientNet B0	0.85	0.9977	0.9987	0.9950	0.9882	0.9583	0.9483	0.9882	0.9821	0.9805	0.9999
CNN	EfficientNet B0	0.90	0.9908	0.9953	0.9943	0.9952	0.9868	0.9999	0.9993	0.9991	0.9988	1.0000
CNN	EfficientNet B0	0.95	0.9995	0.9994	0.9997	0.9977	0.9975	0.9998	1.0000	0.9975	0.9917	1.0000
CNN	EfficientNet B0	1.00	0.9945	0.9993	0.9993	0.9802	0.9974	0.9975	1.0000	0.9962	0.9996	0.9959
CNN	EfficientNet B1	0.05	0.5123	0.4814	0.4072	0.4926	0.6544	0.5223	0.6182	0.4733	0.5364	0.6883
CNN	EfficientNet B1	0.10	0.6943	0.8577	0.1535	0.7190	0.7823	0.3261	0.4823	0.7443	0.7419	0.6872
CNN	EfficientNet B1	0.15	0.7478	0.8197	0.8953	0.4795	0.5056	0.9079	0.7924	0.5631	0.8795	0.9035
CNN	EfficientNet B1	0.20	0.7210	0.9099	0.4593	0.7162	0.6811	0.5622	0.9205	0.5711	0.7989	0.8131
CNN	EfficientNet B1	0.25	0.8807	0.9033	0.8719	0.7966	0.7762	0.8269	0.7524	0.7874	0.8995	0.9333
CNN	EfficientNet B1	0.30	0.9049	0.9086	0.8107	0.8252	0.8795	0.9129	0.9086	0.8965	0.8469	0.9191
CNN	EfficientNet B1	0.35	0.8690	0.9515	0.7898	0.6801	0.7599	0.9612	0.7831	0.9001	0.9110	0.9385
CNN	EfficientNet B1	0.40	0.9409	0.9412	0.8219	0.8275	0.8329	0.8422	0.9482	0.8985	0.9081	0.9849
CNN	EfficientNet B1	0.45	0.8401	0.9415	0.8252	0.8720	0.8814	0.9519	0.9432	0.8705	0.9424	0.9889
CNN	EfficientNet B1	0.50	0.9798	0.9566	0.8965	0.7691	0.8682	0.9729	0.9915	0.9736	0.9423	0.9866
CNN	EfficientNet B1	0.55	0.9013	0.9800	0.8391	0.8777	0.8921	0.9122	0.5611	0.9368	0.8111	0.8843
CNN	EfficientNet B1	0.60	0.9675	0.9776	0.9017	0.9324	0.9375	0.9418	0.9848	0.9814	0.9647	0.9997
CNN	EfficientNet B1	0.65	0.9151	0.9795	0.9509	0.8438	0.9511	0.9865	0.9111	0.9532	0.9707	0.9700
CNN	EfficientNet B1	0.70	0.9886	0.9964	0.9799	0.9546	0.9323	0.9761	0.8595	0.9590	0.9527	0.9999
CNN	EfficientNet B1	0.75	0.9836	0.9934	0.9659	0.9643	0.9778	0.9889	0.9877	0.9837	0.9858	0.9982
CNN	EfficientNet B1	0.80	0.9863	0.9886	0.9866	0.9472	0.9517	0.9769	0.9948	0.9743	0.9724	0.9828
CNN	EfficientNet B1	0.85	0.9986	0.9333	0.9901	0.9863	0.9781	0.9811	0.9936	0.9849	0.9862	0.9996
CNN	EfficientNet B1	0.90	0.9984	0.9977	0.9954	0.9932	0.9891	0.9999	1.0000	0.9970	0.9962	1.0000
CNN	EfficientNet B1	0.95	0.9992	0.9989	0.9982	0.9931	0.9884	0.9982	0.9998	0.9952	0.9919	1.0000
CNN	EfficientNet B1	1.00	0.9991	0.9935	0.9950	0.9937	0.9992	0.9937	0.9996	0.9640	0.9976	1.0000
CNN	EfficientNet B2	0.05	0.5494	0.8645	0.4849	0.4274	0.6190	0.7308	0.5941	0.3269	0.4660	0.6657
CNN	EfficientNet B2	0.10	0.6972	0.8507	0.2254	0.7244	0.6955	0.2878	0.4285	0.7172	0.7331	0.6587
CNN	EfficientNet B2	0.15	0.6768	0.7524	0.5497	0.5571	0.5059	0.8802	0.5800	0.6390	0.7664	0.8207
CNN	EfficientNet B2	0.20	0.7000	0.9512	0.5908	0.6733	0.7593	0.4845	0.4921	0.8677	0.8573	0.8672
CNN	EfficientNet B2	0.25	0.8247	0.8569	0.6395	0.8326	0.7541	0.8678	0.9334	0.8938	0.8886	0.9552
CNN	EfficientNet B2	0.30	0.9087	0.9307	0.8724	0.7825	0.7919	0.7380	0.8092	0.7210	0.7333	0.9983
CNN	EfficientNet B2	0.35	0.8400	0.9739	0.8854	0.4540	0.6242	0.7235	0.7164	0.8154	0.6133	0.9949
CNN	EfficientNet B2	0.40	0.9409	0.8096	0.9500	0.8563	0.5479	0.8565	0.9166	0.9612	0.9285	0.9952
CNN	EfficientNet B2	0.45	0.9171	0.7505	0.8685	0.8290	0.8727	0.8345	0.8954	0.8971	0.8525	0.9395
CNN	EfficientNet B2	0.50	0.8460	0.9558	0.9719	0.9032	0.8626	0.9125	0.9767	0.8959	0.9131	0.9790
CNN	EfficientNet B2	0.55	0.9506	0.9811	0.8792	0.8253	0.6980	0.6320	0.9755	0.8728	0.8824	0.8770
CNN	EfficientNet B2	0.60	0.9523	0.9814	0.9394	0.8717	0.9036	0.9533	0.8732	0.9509	0.9620	0.9979
CNN	EfficientNet B2	0.65	0.9264	0.9867	0.9352	0.9053	0.9385	0.9689	0.7508	0.9794	0.9528	0.9891
CNN	EfficientNet B2	0.70	0.9186	0.9959	0.9359	0.8939	0.9303	0.9336	0.8237	0.9666	0.9405	0.9933
CNN	EfficientNet B2	0.75	0.9898	0.9950	0.9888	0.9636	0.9594	0.9898	0.9986	0.9904	0.9844	0.9960
CNN	EfficientNet B2	0.80	0.9934	0.9946	0.9908	0.9169	0.9308	0.9386	0.9733	0.9795	0.9724	0.9933
CNN	EfficientNet B2	0.85	0.9944	0.9976	0.9973	0.9973	0.9880	0.9878	0.9998	0.9955	0.9991	1.0000
CNN	EfficientNet B2	0.90	0.9983	0.9953	0.9998	0.9963	0.9780	0.9975	0.9977	0.9983	0.9961	1.0000
CNN	EfficientNet B2	0.95	0.9949	0.9926	0.9983	0.9992	0.9976	0.9915	1.0000	0.9993	0.9912	1.0000
CNN	EfficientNet B2	1.00	0.9977	0.9948	0.9965	0.9949	0.9953	0.9779	1.0000	0.9749	0.9909	0.9967
CNN	EfficientNet B3	0.05	0.4968	0.3798	0.5312	0.4083	0.5783	0.6218	0.7011	0.2815	0.4276	0.6525
CNN	EfficientNet B3	0.10	0.7254	0.8349	0.2088	0.5679	0.6688	0.5045	0.7333	0.6541	0.7243	0.8149
CNN	EfficientNet B3	0.15	0.7871	0.8449	0.4397	0.5949	0.5318	0.4287	0.4216	0.7417	0.6309	0.8602
CNN	EfficientNet B3	0.20	0.9196	0.8459	0.6051	0.6711	0.7909	0.8447	0.3569	0.7604	0.7678	0.9208
CNN	EfficientNet B3	0.25	0.9394	0.9038	0.8097	0.7203	0.6073	0.7834	0.8705	0.8188	0.6895	0.9765
CNN	EfficientNet B3	0.30	0.8524	0.8011	0.9392	0.7108	0.6294	0.8560	0.7441	0.8423	0.8565	0.9938
CNN	EfficientNet B3	0.35	0.7299	0.9699	0.6498	0.6786	0.8453	0.8155	0.7869	0.7109	0.7695	0.9580
CNN	EfficientNet B3	0.40	0.8691	0.9635	0.9478	0.8433	0.7355	0.8040	0.9587	0.9538	0.9450	0.9999
CNN	EfficientNet B3	0.45	0.9471	0.9412	0.9028	0.7484	0.7962	0.9177	0.8549	0.9222	0.9557	0.8275
CNN	EfficientNet B3	0.50	0.9626	0.9364	0.9367	0.7504	0.8178	0.9631	0.9093	0.8435	0.9188	0.9751
CNN	EfficientNet B3	0.55	0.9258	0.9188	0.8911	0.8467	0.7951	0.8606	0.9909	0.8180	0.8390	0.8614
CNN	EfficientNet B3	0.60	0.9726	0.9848	0.9134	0.8355	0.7802	0.9018	0.9919	0.9698	0.9690	0.9926
CNN	EfficientNet B3	0.65	0.9411	0.9953	0.9616	0.9063	0.7522	0.9654	0.9256	0.9773	0.9221	0.9326
CNN	EfficientNet B3	0.70	0.9711	0.9579	0.9819	0.7997	0.8927	0.9579	0.9642	0.9655	0.9634	0.9990
CNN	EfficientNet B3	0.75	0.9880	0.9748	0.9705	0.9333	0.9118	0.9430	0.9916	0.9831	0.9657	0.9656
CNN	EfficientNet B3	0.80	0.9933	0.9956	0.9943	0.9195	0.9667	0.9300	0.9938	0.9834	0.9731	0.9846
CNN	EfficientNet B3	0.85	0.9966	0.9919	0.9935	0.9656	0.9807	0.9694	0.9718	0.9930	0.9873	1.0000
CNN	EfficientNet B3	0.90	0.9944	0.9986	0.9980	0.9945	0.9855	0.9873	0.9963	0.9903	0.9901	1.0000
CNN	EfficientNet B3	0.95	0.9999	0.9986	0.9991	0.9966	0.9962	1.0000	1.0000	0.9985	0.9955	1.0000
CNN	EfficientNet B3	1.00	0.9982	0.9986	0.9996	0.9994	0.9999	0.9984	1.0000	0.9733	0.9968	1.0000
CNN	GAN Classifier	0.10	0.7651	0.9425	0.5380	0.7242	0.8234	0.6683	0.8015	0.9217	0.7988	0.9872
CNN	GAN Classifier	0.15	0.8616	0.7816	0.5147	0.6475	0.5478	0.5531	0.4636	0.7321	0.8074	0.7218
CNN	GAN Classifier	0.20	0.8412	0.8776	0.3709	0.8019	0.7156	0.7122	0.6487	0.6157	0.8263	0.8386
CNN	GAN Classifier	0.25	0.9300	0.9408	0.7329	0.7651	0.7195	0.9614	0.9189	0.6469	0.7851	0.9999
CNN	GAN Classifier	0.30	0.8488	0.8877	0.7506	0.7860	0.5231	0.9518	0.7367	0.7562	0.8158	0.9818
CNN	GAN Classifier	0.35	0.8117	0.9495	0.7226	0.5537	0.6264	0.8750	0.8817	0.8340	0.6901	0.9861
CNN	GAN Classifier	0.40	0.8548	0.9059	0.9365	0.8948	0.7913	0.7726	0.9548	0.8897	0.9440	0.9819
CNN	GAN Classifier	0.45	0.8528	0.8532	0.9275	0.9478	0.8640	0.9567	0.9532	0.8553	0.9284	0.7513
CNN	GAN Classifier	0.50	0.9787	0.9837	0.9133	0.9149	0.8054	0.9908	0.8915	0.8969	0.9191	0.9897
CNN	GAN Classifier	0.55	0.9473	0.9441	0.8870	0.8755	0.8416	0.9462	0.8428	0.7935	0.8596	0.8275
CNN	GAN Classifier	0.60	0.9698	0.9842	0.9649	0.9000	0.8302	0.8585	0.9043	0.9511	0.9575	0.9885
CNN	GAN Classifier	0.65	0.9429	0.9640	0.9213	0.9445	0.8431	0.9604	0.7882	0.9652	0.9837	0.9162
CNN	GAN Classifier	0.70	0.9321	0.9564	0.9082	0.9194	0.9155	0.9457	0.7466	0.9736	0.9776	0.9914
CNN	GAN Classifier	0.75	0.9920	0.9947	0.9888	0.9768	0.9076	0.9366	0.9986	0.9841	0.9888	0.9999
CNN	GAN Classifier	0.80	0.9844	0.9873	0.9733	0.9483	0.9632	0.9571	0.9758	0.9903	0.9774	0.9828
CNN	GAN Classifier	0.85	0.9910	0.9102	0.9695	0.9975	0.9767	0.9945	0.9910	0.9874	0.9982	0.9801
CNN	GAN Classifier	0.90	0.9934	0.9955	0.9973	0.9945	0.9696	0.9998	0.9989	0.9985	0.9973	0.9983
CNN	GAN Classifier	0.95	0.9965	1.0000	0.9996	0.9992	0.9971	0.9990	0.9988	0.9997	0.9992	1.0000
CNN	GAN Classifier	1.00	0.9997	0.9998	0.9975	0.9785	0.9999	0.9826	1.0000	0.9999	0.9998	0.9897
CNN	Gradient Boosting	0.05	0.5845	0.8453	0.7598	0.4249	0.5479	0.6471	0.5792	0.8238	0.4277	0.7450
CNN	Gradient Boosting	0.10	0.7914	0.9006	0.8271	0.5020	0.7584	0.7133	0.4511	0.7595	0.5627	0.8635
CNN	Gradient Boosting	0.15	0.8227	0.9620	0.8891	0.5743	0.8273	0.8345	0.9824	0.6842	0.7786	0.9589
CNN	Gradient Boosting	0.20	0.8219	0.9204	0.8036	0.8227	0.8703	0.8497	0.9839	0.6714	0.8485	0.8845
CNN	Gradient Boosting	0.25	0.8058	0.8887	0.8468	0.7657	0.7853	0.7341	0.7660	0.7880	0.7579	0.9859
CNN	Gradient Boosting	0.30	0.8485	0.8511	0.7048	0.9094	0.8071	0.7709	0.9114	0.8049	0.8222	0.9901
CNN	Gradient Boosting	0.35	0.8153	0.9375	0.7678	0.7328	0.8400	0.8217	0.9220	0.8625	0.7754	0.9645
CNN	Gradient Boosting	0.40	0.9354	0.9104	0.7300	0.8817	0.8548	0.7308	0.9791	0.8815	0.8831	0.9977
CNN	Gradient Boosting	0.45	0.9070	0.9581	0.6708	0.7045	0.8464	0.7959	0.8707	0.7604	0.8730	0.9494
CNN	Gradient Boosting	0.50	0.9530	0.9486	0.9304	0.9061	0.9087	0.9730	0.9995	0.8390	0.8809	0.9418
CNN	Gradient Boosting	0.55	0.9354	0.9799	0.8900	0.8917	0.9693	0.9280	0.9177	0.9642	0.8518	0.9681
CNN	Gradient Boosting	0.60	0.9074	0.9763	0.8691	0.8619	0.8783	0.8494	0.9862	0.8855	0.9296	0.9901
CNN	Gradient Boosting	0.65	0.9845	0.9885	0.9047	0.9176	0.9467	0.8461	0.8992	0.9969	0.9457	0.9972
CNN	Gradient Boosting	0.70	0.9865	0.9826	0.9089	0.9428	0.9698	0.6661	0.7770	0.9855	0.9481	0.9963
CNN	Gradient Boosting	0.75	0.9822	0.9965	0.9898	0.9867	0.9445	0.9892	0.9455	0.9899	0.9681	1.0000
CNN	Gradient Boosting	0.80	0.9740	0.9947	0.9897	0.9128	0.9708	0.9592	0.9719	0.9800	0.9773	0.9935
CNN	Gradient Boosting	0.85	0.9900	0.9872	0.9775	0.9492	0.9714	0.9521	0.9662	0.9890	0.9795	0.9958
CNN	Gradient Boosting	0.90	0.9970	0.9861	0.9958	0.9882	0.9800	0.9956	0.9908	1.0000	0.9927	0.9963
CNN	Gradient Boosting	0.95	0.9973	0.9945	0.9921	0.9988	0.9950	0.9984	0.9946	0.9998	0.9983	0.9998
CNN	Gradient Boosting	1.00	0.9873	0.9996	1.0000	0.9993	0.9973	0.9933	0.9979	0.9989	0.9806	0.9968
CNN	MobileNet v1	0.05	0.6588	0.8863	0.6830	0.5500	0.7535	0.8835	0.9710	0.8744	0.5601	0.7850
CNN	MobileNet v1	0.10	0.6957	0.8896	0.9073	0.7709	0.7791	0.8512	0.9875	0.8253	0.7940	0.8703
CNN	MobileNet v1	0.15	0.7993	0.9440	0.7727	0.6958	0.7255	0.8056	0.9040	0.9070	0.8326	0.8828
CNN	MobileNet v1	0.20	0.8813	0.9750	0.8735	0.7748	0.5579	0.9253	0.9608	0.7821	0.8973	0.9729
CNN	MobileNet v1	0.25	0.9221	0.9756	0.9001	0.8194	0.7273	0.8718	0.9083	0.9171	0.9232	0.9939
CNN	MobileNet v1	0.30	0.9575	0.9607	0.7806	0.8862	0.7734	0.9896	0.9287	0.9677	0.9354	0.9985
CNN	MobileNet v1	0.35	0.8765	0.9716	0.9512	0.8187	0.7600	0.9497	0.9321	0.8065	0.9235	0.9856
CNN	MobileNet v1	0.40	0.9661	0.9669	0.8564	0.8582	0.8302	0.8585	0.9592	0.9748	0.9378	0.9995
CNN	MobileNet v1	0.45	0.9475	0.9884	0.9343	0.9581	0.9499	0.8710	0.9667	0.9846	0.9664	1.0000
CNN	MobileNet v1	0.50	0.9790	0.9817	0.9304	0.9171	0.7087	0.9785	0.9047	0.9258	0.9424	0.9925
CNN	MobileNet v1	0.55	0.9631	0.9874	0.8837	0.9199	0.9736	0.9644	0.9527	0.9889	0.9568	0.9997
CNN	MobileNet v1	0.60	0.9966	0.9919	0.9900	0.8968	0.8790	0.9761	0.9974	0.9886	0.9505	0.9982
CNN	MobileNet v1	0.65	0.9808	0.9959	0.9820	0.9335	0.9628	0.9925	0.9939	0.9926	0.9724	0.9997
CNN	MobileNet v1	0.70	0.9882	0.9881	0.9683	0.9587	0.8732	0.9590	0.9932	0.9762	0.9799	0.9990
CNN	MobileNet v1	0.75	0.9933	0.9980	0.9981	0.9548	0.9624	0.9765	0.9949	0.9975	0.9929	1.0000
CNN	MobileNet v1	0.80	0.9925	0.9908	0.9888	0.9640	0.9172	0.9924	0.9825	0.9923	0.9853	1.0000
CNN	MobileNet v1	0.85	0.9995	0.9987	0.9986	0.9874	0.9817	0.9970	1.0000	0.9943	1.0000	1.0000
CNN	MobileNet v1	0.90	0.9956	0.9991	1.0000	0.9942	0.9887	0.9998	1.0000	0.9984	0.9964	1.0000
CNN	MobileNet v1	0.95	0.9990	0.9994	0.9993	0.9990	0.9923	1.0000	1.0000	0.9998	0.9991	1.0000
CNN	MobileNet v1	1.00	1.0000	0.9998	0.9995	0.9999	0.9999	1.0000	1.0000	0.9999	1.0000	1.0000
CNN	MobileNet v1 0.25	0.05	0.6822	0.8369	0.4534	0.6402	0.5505	0.6792	0.6425	0.9079	0.5451	0.8031
CNN	MobileNet v1 0.25	0.10	0.5638	0.8513	0.8973	0.4339	0.5417	0.6711	0.9426	0.8591	0.5301	0.8339
CNN	MobileNet v1 0.25	0.15	0.8715	0.9053	0.8875	0.9012	0.8104	0.7714	0.8494	0.9151	0.8082	0.7090
CNN	MobileNet v1 0.25	0.20	0.8771	0.9422	0.7228	0.7972	0.8926	0.8752	0.9177	0.9126	0.9196	0.9344
CNN	MobileNet v1 0.25	0.25	0.8565	0.9584	0.8692	0.7643	0.6809	0.8666	0.7225	0.9562	0.8849	0.9900
CNN	MobileNet v1 0.25	0.30	0.8649	0.9633	0.8316	0.7575	0.7408	0.9071	0.8965	0.8739	0.9036	0.9568
CNN	MobileNet v1 0.25	0.35	0.8892	0.9228	0.9149	0.6209	0.6511	0.8539	0.9880	0.8270	0.9254	0.9875
CNN	MobileNet v1 0.25	0.40	0.9102	0.9587	0.8969	0.9120	0.8379	0.9651	0.9829	0.9375	0.9143	0.9999
CNN	MobileNet v1 0.25	0.45	0.8821	0.9316	0.9407	0.8069	0.8837	0.9092	0.9588	0.9681	0.9631	0.9980
CNN	MobileNet v1 0.25	0.50	0.9482	0.9769	0.9751	0.8269	0.8379	0.9650	0.9924	0.9322	0.8806	0.9721
CNN	MobileNet v1 0.25	0.55	0.9681	0.9955	0.9267	0.9440	0.9710	0.9689	0.9479	0.9922	0.9668	0.9957
CNN	MobileNet v1 0.25	0.60	0.9685	0.9863	0.9293	0.9304	0.9277	0.9493	0.9625	0.9601	0.9585	0.9910
CNN	MobileNet v1 0.25	0.65	0.9666	0.9795	0.9751	0.9452	0.8871	0.9658	0.9338	0.9601	0.9619	0.9881
CNN	MobileNet v1 0.25	0.70	0.9516	0.9812	0.9743	0.9141	0.8667	0.9750	0.9724	0.9791	0.9530	0.9953
CNN	MobileNet v1 0.25	0.75	0.9833	0.9939	0.9951	0.9323	0.9700	0.9776	0.9889	0.9912	0.9760	1.0000
CNN	MobileNet v1 0.25	0.80	0.9770	0.9727	0.9616	0.8402	0.8249	0.9722	0.9939	0.9847	0.9569	1.0000
CNN	MobileNet v1 0.25	0.85	0.9804	0.9944	0.9912	0.9890	0.9677	0.9953	0.9924	0.9928	0.9898	1.0000
CNN	MobileNet v1 0.25	0.90	0.9743	0.9917	0.9969	0.9836	0.9666	0.9952	0.9917	0.9979	0.9959	0.9999
CNN	MobileNet v1 0.25	0.95	0.9987	0.9889	0.9898	0.9920	0.9712	0.9975	0.9983	0.9962	0.9912	1.0000
CNN	MobileNet v1 0.25	1.00	0.9945	0.9988	0.9975	0.9990	0.9890	0.9992	0.9998	0.9997	0.9907	1.0000
CNN	MobileNet v1 0.5	0.05	0.6828	0.6587	0.9785	0.5941	0.6481	0.7167	0.5775	0.8944	0.4616	0.8205
CNN	MobileNet v1 0.5	0.10	0.6554	0.9018	0.8493	0.6871	0.6138	0.9633	0.9631	0.8596	0.8008	0.6343
CNN	MobileNet v1 0.5	0.15	0.8620	0.8783	0.8464	0.6310	0.6553	0.7524	0.9115	0.8141	0.6963	0.8648
CNN	MobileNet v1 0.5	0.20	0.9123	0.9487	0.7737	0.7879	0.6214	0.9562	0.9374	0.8532	0.7994	0.9764
CNN	MobileNet v1 0.5	0.25	0.8640	0.9532	0.9352	0.7914	0.8625	0.9788	0.9654	0.9246	0.8626	0.9817
CNN	MobileNet v1 0.5	0.30	0.9466	0.9840	0.9683	0.8051	0.7859	0.9327	0.9925	0.9524	0.8999	0.9922
CNN	MobileNet v1 0.5	0.35	0.7375	0.9751	0.9116	0.6840	0.6380	0.9095	0.9849	0.8579	0.8966	0.9744
CNN	MobileNet v1 0.5	0.40	0.9419	0.9705	0.9149	0.8333	0.8136	0.8015	0.9720	0.9236	0.9373	0.9999
CNN	MobileNet v1 0.5	0.45	0.9105	0.9680	0.8743	0.8670	0.8324	0.9647	0.9735	0.9523	0.9380	0.9999
CNN	MobileNet v1 0.5	0.50	0.9538	0.9129	0.9728	0.7465	0.7378	0.9821	0.9939	0.9654	0.9437	0.9995
CNN	MobileNet v1 0.5	0.55	0.9735	0.9905	0.8356	0.8941	0.9047	0.9787	0.9876	0.9784	0.9612	0.9988
CNN	MobileNet v1 0.5	0.60	0.9834	0.9835	0.9112	0.9139	0.8899	0.9372	0.9933	0.9783	0.9684	0.9988
CNN	MobileNet v1 0.5	0.65	0.9857	0.9940	0.9856	0.9432	0.8934	0.9883	0.9559	0.9781	0.9607	0.9986
CNN	MobileNet v1 0.5	0.70	0.9728	0.9929	0.9883	0.9192	0.9158	0.9829	0.9912	0.9633	0.9666	0.9989
CNN	MobileNet v1 0.5	0.75	0.9897	0.9919	0.9923	0.9561	0.9109	0.9855	0.9991	0.9928	0.9794	1.0000
CNN	MobileNet v1 0.5	0.80	0.9938	0.9855	0.9977	0.9621	0.9286	0.9921	0.9911	0.9870	0.9931	1.0000
CNN	MobileNet v1 0.5	0.85	0.9956	0.9932	0.9948	0.9621	0.9650	0.9969	0.9955	0.9936	0.9911	1.0000
CNN	MobileNet v1 0.5	0.90	0.9949	0.9969	0.9992	0.9946	0.9710	0.9993	1.0000	0.9984	0.9979	1.0000
CNN	MobileNet v1 0.5	0.95	0.9974	0.9996	0.9985	0.9992	0.9959	1.0000	1.0000	0.9999	0.9982	1.0000
CNN	MobileNet v1 0.5	1.00	0.9986	0.9988	0.9992	0.9997	0.9989	0.9997	1.0000	0.9997	0.9960	1.0000
CNN	MobileNet v1 0.75	0.05	0.6442	0.7183	0.6814	0.5290	0.5898	0.9804	0.7736	0.8212	0.5958	0.7322
CNN	MobileNet v1 0.75	0.10	0.7612	0.9560	0.4703	0.6587	0.6431	0.7629	0.6502	0.9330	0.6697	0.8744
CNN	MobileNet v1 0.75	0.15	0.7246	0.9497	0.6554	0.7785	0.6702	0.8211	0.9580	0.8978	0.8775	0.5734
CNN	MobileNet v1 0.75	0.20	0.8427	0.9204	0.8880	0.7182	0.7761	0.4929	0.8916	0.8120	0.8774	0.9951
CNN	MobileNet v1 0.75	0.25	0.9185	0.9592	0.6037	0.8388	0.7526	0.7646	0.9206	0.9426	0.8878	0.9987
CNN	MobileNet v1 0.75	0.30	0.8458	0.9243	0.5931	0.8085	0.6824	0.9222	0.8939	0.9324	0.8314	0.9962
CNN	MobileNet v1 0.75	0.35	0.9073	0.9578	0.9569	0.6360	0.7216	0.9236	0.9837	0.7718	0.9016	0.9949
CNN	MobileNet v1 0.75	0.40	0.9521	0.9760	0.9182	0.8305	0.8329	0.9435	0.9822	0.9727	0.9454	1.0000
CNN	MobileNet v1 0.75	0.45	0.9338	0.9822	0.9627	0.8422	0.7984	0.9136	0.9854	0.9743	0.9388	0.9987
CNN	MobileNet v1 0.75	0.50	0.9589	0.9854	0.9800	0.8290	0.9232	0.9930	0.9448	0.9648	0.9557	0.9794
CNN	MobileNet v1 0.75	0.55	0.9778	0.9900	0.7901	0.8993	0.9789	0.9431	0.9195	0.9722	0.9637	0.9992
CNN	MobileNet v1 0.75	0.60	0.9586	0.9915	0.9783	0.9552	0.9176	0.9300	0.9892	0.9752	0.9641	0.9997
CNN	MobileNet v1 0.75	0.65	0.9806	0.9955	0.9843	0.9683	0.9555	0.9881	0.9679	0.9952	0.9798	1.0000
CNN	MobileNet v1 0.75	0.70	0.9834	0.9984	0.9852	0.8912	0.8960	0.9821	0.9397	0.9751	0.9682	0.9999
CNN	MobileNet v1 0.75	0.75	0.9908	0.9962	0.9937	0.9474	0.9525	0.9913	0.9986	0.9868	0.9888	1.0000
CNN	MobileNet v1 0.75	0.80	0.9955	0.9957	0.9973	0.9440	0.9497	0.9868	0.9924	0.9938	0.9914	1.0000
CNN	MobileNet v1 0.75	0.85	0.9999	0.9968	0.9998	0.9950	0.9626	0.9992	1.0000	0.9971	0.9997	1.0000
CNN	MobileNet v1 0.75	0.90	0.9954	0.9932	1.0000	0.9965	0.9570	0.9999	0.9999	0.9980	0.9952	1.0000
CNN	MobileNet v1 0.75	0.95	0.9927	0.9968	0.9998	0.9989	0.9914	1.0000	0.9998	0.9993	0.9980	1.0000
CNN	MobileNet v1 0.75	1.00	1.0000	0.9999	0.9995	0.9997	0.9997	1.0000	1.0000	1.0000	0.9999	1.0000
CNN	Random Forest	0.05	0.8264	0.8943	0.9116	0.5385	0.9329	0.8930	0.9191	0.9531	0.8089	0.9982
CNN	Random Forest	0.10	0.9166	0.9412	0.9728	0.6731	0.9397	0.8346	0.9853	0.8686	0.8416	0.9985
CNN	Random Forest	0.15	0.9466	0.9662	0.9706	0.7436	0.9660	0.8489	0.9840	0.8280	0.9174	0.9950
CNN	Random Forest	0.20	0.8871	0.9590	0.9473	0.6991	0.9467	0.8115	0.9852	0.8194	0.8922	0.9982
CNN	Random Forest	0.25	0.8867	0.9715	0.9869	0.9034	0.9423	0.9119	0.9937	0.9052	0.9365	0.9996
CNN	Random Forest	0.30	0.9351	0.9609	0.9694	0.8978	0.9346	0.9317	0.9938	0.9102	0.9122	0.9982
CNN	Random Forest	0.35	0.9585	0.9783	0.9698	0.7302	0.9590	0.9229	0.9812	0.9054	0.9088	0.9976
CNN	Random Forest	0.40	0.9615	0.9874	0.9637	0.8597	0.9323	0.8914	0.9925	0.9589	0.9527	0.9984
CNN	Random Forest	0.45	0.9691	0.9894	0.9589	0.8354	0.9402	0.9665	0.9960	0.9441	0.9665	0.9976
CNN	Random Forest	0.50	0.9813	0.9806	0.9818	0.8965	0.9588	0.9572	0.9982	0.9797	0.9559	1.0000
CNN	Random Forest	0.55	0.9959	0.9938	0.9814	0.9574	0.9699	0.9511	0.9896	0.9933	0.9802	0.9999
CNN	Random Forest	0.60	0.9937	0.9925	0.9902	0.9304	0.9746	0.9396	0.9992	0.9866	0.9729	1.0000
CNN	Random Forest	0.65	0.9965	0.9965	0.9814	0.9724	0.9732	0.9694	0.9940	0.9953	0.9838	0.9999
CNN	Random Forest	0.70	0.9982	0.9987	0.9944	0.9721	0.9793	0.9604	0.9938	0.9966	0.9737	1.0000
CNN	Random Forest	0.75	0.9953	0.9980	0.9988	0.9593	0.9557	0.9838	0.9990	0.9978	0.9773	1.0000
CNN	Random Forest	0.80	0.9963	0.9981	0.9986	0.9604	0.9615	0.9802	0.9974	0.9968	0.9955	1.0000
CNN	Random Forest	0.85	0.9987	0.9997	0.9994	0.9872	0.9696	0.9854	0.9989	0.9973	0.9930	1.0000
CNN	Random Forest	0.90	0.9984	0.9985	0.9999	0.9981	0.9701	0.9966	0.9999	0.9991	0.9956	1.0000
CNN	Random Forest	0.95	0.9997	0.9996	0.9998	1.0000	0.9946	1.0000	1.0000	0.9999	0.9991	1.0000
CNN	Random Forest	1.00	0.9998	0.9999	1.0000	0.9999	0.9990	1.0000	1.0000	0.9996	0.9996	1.0000
CNN	ResNet18	0.05	0.5810	0.7775	0.4431	0.5207	0.7148	0.8872	0.4696	0.5286	0.5994	0.5979
CNN	ResNet18	0.10	0.7598	0.9478	0.9374	0.7272	0.6878	0.8670	0.6357	0.9656	0.5510	0.7344
CNN	ResNet18	0.15	0.7426	0.8901	0.6279	0.7737	0.7556	0.5438	0.9620	0.7974	0.8773	0.7288
CNN	ResNet18	0.20	0.8714	0.9285	0.6146	0.4542	0.6398	0.9292	0.8920	0.5011	0.8070	0.9349
CNN	ResNet18	0.25	0.9141	0.9672	0.7292	0.8037	0.6324	0.9706	0.9526	0.8791	0.9384	0.9970
CNN	ResNet18	0.30	0.8964	0.8824	0.6125	0.6392	0.5780	0.9276	0.6961	0.8085	0.8216	0.9715
CNN	ResNet18	0.35	0.8771	0.9679	0.8812	0.7418	0.7271	0.8570	0.6845	0.5930	0.7400	0.9682
CNN	ResNet18	0.40	0.9458	0.9145	0.9593	0.7852	0.6274	0.8598	0.9953	0.9543	0.9330	1.0000
CNN	ResNet18	0.45	0.8111	0.8637	0.8568	0.7377	0.7543	0.9677	0.9467	0.8444	0.9585	0.9635
CNN	ResNet18	0.50	0.9447	0.9624	0.8670	0.7771	0.7037	0.9587	0.9992	0.7771	0.8410	0.9836
CNN	ResNet18	0.55	0.9852	0.9545	0.8227	0.8842	0.8464	0.9091	0.8841	0.9833	0.9025	0.8963
CNN	ResNet18	0.60	0.9794	0.9781	0.9818	0.9075	0.8179	0.8432	0.9934	0.9725	0.9488	0.9954
CNN	ResNet18	0.65	0.9664	0.9834	0.9476	0.8985	0.8891	0.9725	0.9392	0.9716	0.9251	0.9654
CNN	ResNet18	0.70	0.9542	0.9958	0.9940	0.7892	0.7892	0.9685	0.5848	0.9093	0.8860	0.9994
CNN	ResNet18	0.75	0.9908	0.9960	0.9909	0.9554	0.8797	0.9869	0.9999	0.9983	0.9960	1.0000
CNN	ResNet18	0.80	0.9953	0.9949	0.9956	0.9160	0.9497	0.9485	0.9894	0.9868	0.9900	0.9828
CNN	ResNet18	0.85	0.9995	0.9070	0.9977	0.9591	0.9696	0.9956	0.9958	0.9960	0.9984	1.0000
CNN	ResNet18	0.90	0.9981	0.9996	0.9998	0.9952	0.9851	0.9971	0.9825	0.9989	0.9984	0.9993
CNN	ResNet18	0.95	0.9978	0.9996	0.9974	0.9999	0.9935	0.9990	1.0000	0.9994	0.9936	1.0000
CNN	ResNet18	1.00	0.9980	0.9960	0.9994	1.0000	0.9999	0.9833	0.9998	0.9797	1.0000	1.0000
CNN	SVM	0.05	0.8200	0.9208	0.9622	0.7015	0.9415	0.9461	0.9970	0.9730	0.6998	0.9893
CNN	SVM	0.10	0.8535	0.9631	0.9750	0.7541	0.8977	0.8961	0.9250	0.8945	0.9150	0.9995
CNN	SVM	0.15	0.9228	0.9632	0.9216	0.8267	0.9342	0.9425	0.9766	0.8416	0.9427	0.9997
CNN	SVM	0.20	0.8955	0.9686	0.9150	0.8875	0.9554	0.9468	0.9821	0.7895	0.9299	0.9999
CNN	SVM	0.25	0.9326	0.9797	0.9789	0.9013	0.9396	0.9581	0.9998	0.8815	0.9312	1.0000
CNN	SVM	0.30	0.9229	0.9673	0.9662	0.8231	0.9298	0.9232	0.9548	0.9406	0.9420	1.0000
CNN	SVM	0.35	0.9836	0.9799	0.9505	0.9008	0.9351	0.9476	0.9988	0.9415	0.9402	1.0000
CNN	SVM	0.40	0.9761	0.9800	0.9593	0.8922	0.9222	0.8973	0.9983	0.9540	0.9624	1.0000
CNN	SVM	0.45	0.9804	0.9882	0.9670	0.9493	0.9350	0.9727	0.9988	0.9753	0.9828	0.9999
CNN	SVM	0.50	0.9898	0.9898	0.9796	0.8865	0.9009	0.9906	1.0000	0.9890	0.9722	1.0000
CNN	SVM	0.55	0.9935	0.9896	0.9766	0.9731	0.9853	0.9719	0.9966	0.9939	0.9932	0.9999
CNN	SVM	0.60	0.9969	0.9956	0.9991	0.9770	0.9780	0.9642	0.9996	0.9908	0.9918	1.0000
CNN	SVM	0.65	0.9918	0.9971	0.9919	0.9508	0.9542	0.9832	0.9934	0.9994	0.9852	0.9889
CNN	SVM	0.70	0.9948	0.9977	0.9964	0.9373	0.9757	0.9789	0.9934	0.9932	0.9756	1.0000
CNN	SVM	0.75	0.9994	0.9992	0.9992	0.9805	0.9716	0.9856	1.0000	0.9991	0.9968	1.0000
CNN	SVM	0.80	0.9996	0.9936	0.9980	0.9742	0.9871	0.9906	0.9997	0.9960	0.9964	1.0000
CNN	SVM	0.85	1.0000	0.9997	1.0000	0.9937	0.9923	0.9940	0.9999	0.9998	0.9995	1.0000
CNN	SVM	0.90	0.9999	0.9998	0.9999	0.9986	0.9951	0.9996	1.0000	0.9990	0.9995	1.0000
CNN	SVM	0.95	0.9999	1.0000	1.0000	1.0000	0.9995	1.0000	1.0000	1.0000	1.0000	1.0000
CNN	SVM	1.00	1.0000	1.0000	1.0000	1.0000	1.0000	1.0000	1.0000	1.0000	1.0000	1.0000
CNN	U-Net	0.05	0.5923	0.9137	0.7079	0.5364	0.6387	0.7461	0.6287	0.8124	0.5292	0.6926
CNN	U-Net	0.10	0.7884	0.9405	0.6341	0.7085	0.7539	0.6723	0.7625	0.8968	0.8447	0.6275
CNN	U-Net	0.15	0.7993	0.8906	0.4584	0.7267	0.7074	0.8790	0.9904	0.8515	0.8651	0.8097
CNN	U-Net	0.20	0.8945	0.9367	0.5590	0.7653	0.8174	0.9521	0.9472	0.8978	0.9077	0.9920
CNN	U-Net	0.25	0.8027	0.9379	0.9661	0.7551	0.7429	0.7145	0.8795	0.8691	0.8634	1.0000
CNN	U-Net	0.30	0.8920	0.8637	0.7344	0.7671	0.5710	0.8902	0.9568	0.8039	0.8285	0.9964
CNN	U-Net	0.35	0.9478	0.9837	0.9178	0.8659	0.5725	0.9449	0.9803	0.9722	0.9319	0.9957
CNN	U-Net	0.40	0.8274	0.9208	0.7992	0.7359	0.7750	0.7418	0.9135	0.9763	0.9304	0.9970
CNN	U-Net	0.45	0.9125	0.9647	0.8998	0.8022	0.5938	0.9336	0.9821	0.9664	0.9617	1.0000
CNN	U-Net	0.50	0.9816	0.9852	0.9917	0.9169	0.8519	0.9649	0.9473	0.9609	0.9365	0.9998
CNN	U-Net	0.55	0.9442	0.9869	0.9518	0.9696	0.8905	0.9223	0.9830	0.9240	0.9279	0.8919
CNN	U-Net	0.60	0.9831	0.9915	0.9940	0.9902	0.9073	0.9619	0.9934	0.9848	0.9700	1.0000
CNN	U-Net	0.65	0.9683	0.9545	0.9907	0.9173	0.9278	0.9797	0.9871	0.9932	0.9744	0.9993
CNN	U-Net	0.70	0.9782	0.9862	0.9892	0.8623	0.8951	0.9401	0.9489	0.9874	0.9673	0.9994
CNN	U-Net	0.75	0.9927	0.9746	0.9964	0.9003	0.9204	0.9760	0.9990	0.9852	0.9841	0.9999
CNN	U-Net	0.80	0.9849	0.9940	0.9895	0.9540	0.9513	0.9974	1.0000	0.9897	0.9743	0.9982
CNN	U-Net	0.85	0.9943	0.9949	0.9998	0.9812	0.9474	0.9909	0.9995	0.9919	0.9858	1.0000
CNN	U-Net	0.90	0.9919	0.9983	0.9968	0.9630	0.9444	0.9937	0.9995	0.9861	0.9594	1.0000
CNN	U-Net	0.95	0.9984	0.9991	0.9985	0.9953	0.9878	0.9986	0.9995	1.0000	0.9912	1.0000
CNN	U-Net	1.00	0.9712	0.9994	0.9995	0.9995	0.9778	0.9983	1.0000	0.9785	0.9649	0.9987
CNN	XGBoost	0.05	0.6425	0.7694	0.6423	0.4094	0.5916	0.8569	0.5413	0.8630	0.7382	0.8445
CNN	XGBoost	0.10	0.7829	0.6933	0.7915	0.5475	0.7101	0.8437	0.8613	0.7655	0.5267	0.8597
CNN	XGBoost	0.15	0.8841	0.8731	0.6880	0.5199	0.9119	0.8225	0.9565	0.6737	0.7246	0.9732
CNN	XGBoost	0.20	0.9230	0.8774	0.8477	0.8119	0.8748	0.7482	0.9924	0.7010	0.8930	0.9251
CNN	XGBoost	0.25	0.8681	0.9465	0.7143	0.8237	0.8275	0.9175	0.7259	0.9292	0.8823	0.9993
CNN	XGBoost	0.30	0.8913	0.8962	0.7303	0.8295	0.9378	0.8761	0.9498	0.8770	0.8707	0.9946
CNN	XGBoost	0.35	0.9202	0.9754	0.8200	0.8634	0.9488	0.8737	0.9713	0.8395	0.8788	0.9752
CNN	XGBoost	0.40	0.9662	0.9462	0.7717	0.9058	0.8913	0.9179	0.9943	0.9561	0.9586	0.9944
CNN	XGBoost	0.45	0.9073	0.9852	0.8486	0.7289	0.9238	0.8077	0.9447	0.7221	0.8645	0.9337
CNN	XGBoost	0.50	0.9893	0.9484	0.9528	0.9138	0.9757	0.9453	0.9975	0.9605	0.9619	0.9891
CNN	XGBoost	0.55	0.9597	0.9841	0.9534	0.9705	0.9743	0.9823	0.9860	0.9893	0.9370	0.9890
CNN	XGBoost	0.60	0.9867	0.9869	0.9291	0.9438	0.9383	0.9459	0.9986	0.9677	0.9567	1.0000
CNN	XGBoost	0.65	0.9775	0.9923	0.9298	0.9292	0.9552	0.9298	0.9351	0.9942	0.9693	0.9999
CNN	XGBoost	0.70	0.9772	0.9956	0.9566	0.9636	0.9554	0.8664	0.9727	0.9933	0.9497	0.9996
CNN	XGBoost	0.75	0.9952	0.9947	0.9984	0.9769	0.9595	0.9903	0.9978	0.9961	0.9840	1.0000
CNN	XGBoost	0.80	0.9915	0.9963	0.9994	0.9427	0.9776	0.9836	0.9997	0.9923	0.9879	1.0000
CNN	XGBoost	0.85	0.9962	0.9970	0.9962	0.9861	0.9844	0.9778	0.9981	0.9984	0.9896	1.0000
CNN	XGBoost	0.90	0.9951	0.9991	0.9938	0.9843	0.9886	0.9968	0.9976	0.9985	0.9970	0.9994
CNN	XGBoost	0.95	0.9995	0.9998	0.9995	0.9992	0.9964	1.0000	1.0000	0.9996	0.9983	1.0000
CNN	XGBoost	1.00	0.9983	0.9930	1.0000	0.9979	0.9974	0.9984	1.0000	0.9985	0.9951	1.0000

**Table A3:** Model Performance Metrics and Timing Results (Cross Validation Experiment).

Pretext Model	Downstream Model	Accuracy	Precision	Recall	F1 Score	ROC AUC	TPR@FPR = 0.01	TPR@FPR = 0.05	TPR@FPR = 0.10	TPR@FPR = 0.15	Test Feat. Ext. (ms/img)	Inference Time (ms/img)
CNN	XGBoost	0.9565	0.9592	0.9564	0.9567	0.9987	0.9761	0.9978	0.9989	0.9989	15.35	0.04
CNN	Random Forest	0.9696	0.9705	0.9694	0.9694	0.9986	0.9747	0.9957	0.9978	0.9989	15.35	0.05
CNN	SVM	0.9913	0.9917	0.9913	0.9914	0.9999	0.9989	1.0000	1.0000	1.0000	15.35	0.08
CNN	Gradient Boosting	0.9413	0.9467	0.9412	0.9413	0.9959	0.9543	0.9815	0.9902	0.9935	15.35	0.01
CNN	ResNet18	0.9652	0.9680	0.9654	0.9655	0.9994	0.9848	0.9978	1.0000	1.0000	15.35	0.13
CNN	CNN	0.9022	0.9069	0.9022	0.9026	0.9916	0.8946	0.9739	0.9870	0.9880	15.35	0.03
CNN	U-Net	0.8793	0.8897	0.8795	0.8788	0.9924	0.8511	0.9598	0.9848	0.9946	15.35	0.35
CNN	MobileNet v1	0.9554	0.9598	0.9552	0.9554	0.9984	0.9707	0.9967	0.9967	0.9978	15.35	0.05
CNN	MobileNet v1 0.75	0.9261	0.9317	0.9264	0.9267	0.9976	0.9337	0.9935	0.9967	0.9978	15.35	0.05
CNN	MobileNet v1 0.5	0.9359	0.9385	0.9359	0.9358	0.9972	0.9522	0.9902	0.9957	0.9978	15.35	0.11
CNN	MobileNet v1 0.25	0.8804	0.8882	0.8809	0.8788	0.9910	0.8326	0.9576	0.9826	0.9902	15.35	0.05
CNN	EfficientNet B0	0.9337	0.9383	0.9338	0.9335	0.9968	0.9402	0.9913	0.9946	0.9967	15.35	0.16
CNN	EfficientNet B1	0.9315	0.9353	0.9315	0.9311	0.9969	0.9402	0.9870	0.9924	0.9957	15.35	0.22
CNN	EfficientNet B2	0.9109	0.9206	0.9116	0.9115	0.9947	0.9109	0.9761	0.9891	0.9957	15.35	0.25
CNN	EfficientNet B3	0.9174	0.9262	0.9177	0.9178	0.9960	0.9217	0.9804	0.9946	0.9967	15.35	0.28
CNN	GAN Classifier	0.9533	0.9578	0.9536	0.9536	0.9978	0.9663	0.9957	0.9967	0.9989	15.35	0.04

**Table A4:** Class-wise ROC AUC Results (Cross Validation Experiment).

Pretext Model	Downstream Model	ROC AUC Class 0	ROC AUC Class 1	ROC AUC Class 2	ROC AUC Class 3	ROC AUC Class 4	ROC AUC Class 5	ROC AUC Class 6	ROC AUC Class 7	ROC AUC Class 8	ROC AUC Class 9
CNN	XGBoost	0.9941	0.9997	0.9996	0.9988	0.9984	0.9999	1.0000	0.9984	0.9976	0.9999
CNN	Random Forest	0.9960	0.9987	0.9997	0.9972	0.9981	0.9998	1.0000	0.9974	0.9981	0.9999
CNN	SVM	0.9999	0.9999	0.9999	1.0000	0.9999	1.0000	1.0000	0.9998	1.0000	0.9999
CNN	Gradient Boosting	0.9880	0.9946	0.9953	0.9986	0.9959	0.9986	0.9987	0.9986	0.9974	0.9996
CNN	ResNet18	0.9995	0.9995	0.9999	0.9999	0.9974	1.0000	1.0000	0.9991	0.9988	0.9999
CNN	CNN	0.9830	0.9869	0.9849	0.9858	0.9879	0.9948	0.9958	0.9976	0.9906	0.9991
CNN	U-Net	0.9805	0.9979	0.9993	0.9872	0.9892	0.9928	0.9982	0.9963	0.9845	0.9999
CNN	MobileNet v1	0.9966	0.9992	0.9999	0.9989	0.9975	0.9997	1.0000	0.9968	0.9959	0.9999
CNN	MobileNet v1 0.75	0.9946	0.9991	0.9989	0.9963	0.9958	0.9978	0.9999	0.9954	0.9968	1.0000
CNN	MobileNet v1 0.5	0.9985	0.9978	0.9988	0.9923	0.9917	0.9989	1.0000	0.9953	0.9931	1.0000
CNN	MobileNet v1 0.25	0.9886	0.9962	0.9990	0.9813	0.9719	0.9951	0.9984	0.9892	0.9846	1.0000
CNN	EfficientNet B0	0.9965	0.9998	0.9963	0.9947	0.9919	0.9994	0.9997	0.9943	0.9965	0.9947
CNN	EfficientNet B1	0.9919	0.9997	0.9987	0.9953	0.9930	0.9972	0.9992	0.9971	0.9951	1.0000
CNN	EfficientNet B2	0.9882	0.9971	0.9974	0.9895	0.9893	0.9966	0.9992	0.9932	0.9925	1.0000
CNN	EfficientNet B3	0.9966	0.9980	0.9989	0.9918	0.9922	0.9976	0.9994	0.9922	0.9940	0.9999
CNN	GAN Classifier	0.9995	0.9998	0.9997	0.9967	0.9982	0.9991	0.9998	0.9975	0.9965	0.9942

**Table A5:** SimCLR Performance Metrics.

*k*	Accuracy	Precision	Recall	F1 Score
0.05	0.1577	0.1233	0.1577	0.0863
0.10	0.3135	0.3633	0.3135	0.2360
0.15	0.3210	0.5349	0.3210	0.2633
0.20	0.4545	0.5505	0.4545	0.4222
0.25	0.4675	0.7500	0.4675	0.4763
0.30	0.5900	0.6891	0.5900	0.5763
0.35	0.6085	0.6602	0.6085	0.5994
0.40	0.6382	0.6655	0.6382	0.6192
0.45	0.5436	0.6348	0.5436	0.5211
0.50	0.6846	0.7175	0.6846	0.6719
0.55	0.7124	0.7541	0.7124	0.7120
0.60	0.7551	0.7975	0.7551	0.7524
0.65	0.7532	0.7792	0.7532	0.7490
0.70	0.7514	0.7698	0.7514	0.7449
0.75	0.6809	0.7821	0.6809	0.6779
0.80	0.7737	0.8043	0.7737	0.7690
0.85	0.8126	0.8278	0.8126	0.8060
0.90	0.8738	0.8783	0.8738	0.8719
0.95	0.8794	0.8863	0.8794	0.8779
1.00	0.9202	0.9215	0.9202	0.9199
